# Development of a new version of the Liverpool Malaria Model. II. Calibration and validation for West Africa

**DOI:** 10.1186/1475-2875-10-62

**Published:** 2011-03-16

**Authors:** Volker Ermert, Andreas H Fink, Anne E Jones, Andrew P Morse

**Affiliations:** 1 Institute of Geophysics and Meteorology, University of Cologne, Cologne, Germany; 2 School of Environmental Sciences, University of Liverpool, Liverpool, UK

## Abstract

**Background:**

In the first part of this study, an extensive literature survey led to the construction of a new version of the *Liverpool Malaria Model *(LMM). A new set of parameter settings was provided and a new development of the mathematical formulation of important processes related to the vector population was performed within the LMM. In this part of the study, so far undetermined model parameters are calibrated through the use of data from field studies. The latter are also used to validate the new LMM version, which is furthermore compared against the original LMM version.

**Methods:**

For the calibration and validation of the LMM, numerous entomological and parasitological field observations were gathered for West Africa. Continuous and quality-controlled temperature and precipitation time series were constructed using intermittent raw data from 34 weather stations across West Africa. The meteorological time series served as the LMM data input. The skill of LMM simulations was tested for 830 different sets of parameter settings of the undetermined LMM parameters. The model version with the highest skill score in terms of entomological malaria variables was taken as the final setting of the new LMM version.

**Results:**

Validation of the new LMM version in West Africa revealed that the simulations compare well with entomological field observations. The new version reproduces realistic transmission rates and simulated malaria seasons are comparable to field observations. Overall the new model version performs much better than the original model. The new model version enables the detection of the epidemic malaria potential at fringes of endemic areas and, more importantly, it is now applicable to the vast area of malaria endemicity in the humid African tropics.

**Conclusions:**

A review of entomological and parasitological data from West Africa enabled the construction of a new LMM version. This model version represents a significant step forward in the modelling of a weather-driven malaria transmission cycle. The LMM is now more suitable for the use in malaria early warning systems as well as for malaria projections based on climate change scenarios, both in epidemic and endemic malaria areas.

## Background

The World Health Organization (WHO) estimated that about two billion people, that is more than 40% of the total world population, are exposed to malaria [1]. Estimates in terms of 2009 revealed that this mosquito-borne disease causes about 225 million cases and 781,000 deaths annually. At least 90% of the worldwide malaria deaths occur in sub-Saharan Africa [2].

Malaria is a climate-sensitive tropical disease and hence climate exerts a strong impact upon the distribution of malaria transmission in space and time [3]. An assessment of current and future malaria risk is an important topic in the area of research relating climate to disease risk [4]. Reliable forecasts of epidemic malaria outbreaks on seasonal timescales [5] and assessments of disease vulnerability over decadal timescales are needed [6]. However, this requires the production of a weather-malaria modelling system. World-leading numerical weather forecast centres have already demonstrated useful skill in forecasts far beyond a month lead-time for some tropical regions [7], and climate projections are becoming more reliable [8]. Advances have also been achieved in weather- or climate-driven malaria modelling. For example, Hoshen and Morse [9] introduced the *Liverpool Malaria Model *(LMM), which is a mathematical-biological model of malaria parasite dynamics driven by daily temperature and precipitation data. However, further progress must be obtained in order to enable skilful malaria simulations for epidemic and endemic malaria regions based on meteorological data. For this reason, the present study introduces the *LMM version of 2010 *(henceforth called LMM_2010_), which simulates a more realistic spread of malaria in space and time and is hence a useful tool for a weather- or climate-disease modelling system.

In the first part of this study [10], an extensive literature review enabled the construction of a set of refined parameter settings (see Table 1) and an extended mathematical formulation of the LMM. Important sub-modules of the original LMM version were reviewed and updated. The oviposition, as well as the survival of immature mosquitoes, were adjusted to field conditions via the application of a fuzzy distribution model. In the second part of this study (this paper) previously undetermined model parameters are calibrated and the LMM_2010 _is validated by means of entomological and parasitological field observations from West Africa.

**Table 1:** LMM parameters and mathematical formulations

sym	parameter	**val**_**2004**_	**val**_**2010**_
*D*_*gH*_	humid degree days of the gonotrophic cycle	37.1 degree days	37.1 degree days
*D*_*gL*_	dry degree days of the gonotrophic cycle	65.4 degree days	65.4 degree days
*T*_*gH*_	humid gonotrophic temperature threshold	7.7°C	7.7°C
*T*_*gL*_	dry gonotrophic temperature threshold	4.5°C	4.5°C
*R*_*-*_	10-day accumulated precipitation threshold	10 mm	10 mm
*R*_•_	rainfall laying multiplier	1.0	NU
#*E*_*p*_	number of produced eggs per female mosquito	NU	*120 *eggs
#*E*_*o*_	number of oviposited eggs per female mosquito	NU	**Eq. Two **in [10]
*U*_*1*_	lower threshold of unsuitable rainfall conditions (fuzzy distribution model)	NU	**0 **mm
*S*	most suitable rainfall condition (fuzzy distribution model)	NU	*10 *mm
*U*_*2*_	upper threshold of unsuitable rainfall conditions (fuzzy distribution model)	NU	*500 *mm
*CAP*	cap on the number of fertile mosquitoes	10,000 mosquitoes	*400 *mosquitoes
*MMA*	mosquito mature age	15 days	**12 **days
η_*d*,¬*R*_	rainfall independent immature daily mosquito survival probability	NU	**82.5**%
η_*d*_	daily immature mosquito survival probability (in %)	Eq. Three in [10]	**Eq. Four **in [10]
*p*_*d*_	daily mosquito survival probability (in %)	Martens I (see [10])	**Martens II **(see [10])
*p*_*d*↓_	dry season mosquito survival probability shift	NU	-*10*%
*D*_*s*_	degree-days of the sporogonic cycle	111 degree days	111 degree days
*T*_*s*_	sporogonic temperature threshold	18°C	**16**°C
*a*	human blood index	50%	**80**%
*b*	mosquito-to-human transmission efficiency	50%	**30**%
*c*_*a→c*_	adult-child conversion rate	NU	**0.5**
*HIA*	human infectious age	14 days	**20 **days
*r*	daily human recovery rate	0.0284 day ^1^	**0.0050 **day^-1^
*GF*	fraction of gametocyte carriers	NU	**50**%
*c*	human-to-mosquito transmission efficiency	50%	**20**%
*tr*_*im*_	trickle of the number of added infectious mosquitoes	1.01 mosquitoes	1.01 mosquitoes

LMM model parameters and mathematical formulations with regard to their original (Hoshen and Morse 2004) and new settings. Columns: sym: symbol of the model parameter; parameter: name of the parameter; unit: unit; val_2004_: LMM_2004 _value; val_2010_: LMM_2010 _value. Abbreviations: NU: not used. Parameter values in *italics *refer to calibrated values and those values and mathematical formulations in **bold **were determined in the first part of this study [10].

## Methods

### Data sources

#### Time series of meteorological stations

**Figure 1:**
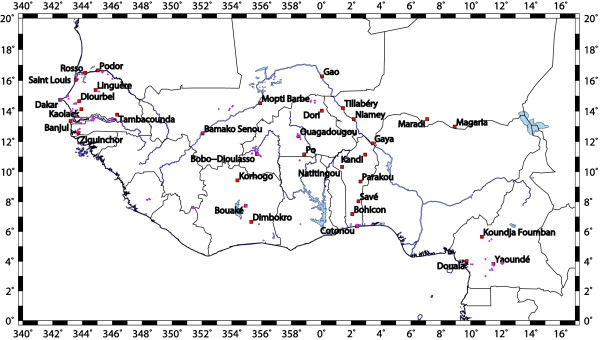
**Meteorological and malaria observations**. Map showing locations of the synoptic weather station used in this study (see Additional file 1) as well as the locations of entomological and parasitological field studies (purple dots; see Additional file 2).

In the present study, temperature and precipitation measurements from synoptic weather stations across West Africa (see Figure 1) were used as LMM data input. Weather station data were gathered from the archive of the *German Weather Service *(DWD; German Weather Service) as well as from the *Federal climate complex Global Surface Summary of Day version 7 *(GSOD; from the US National Climate Data Centre) data. The meteorological time series were quality-checked and missing values were filled according to a specific procedure, which is described by Ermert [11]. The resulting analysis provides continuous and quality-controlled *temperature *(*T*) and *rainfall *(*R*) time series between 1973 and 2006 for 34 synoptic weather stations in West Africa (see Figure 1 and Additional file 1).

**Figure 2:**
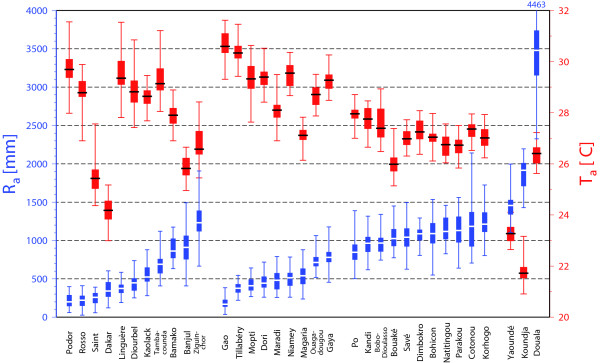
**Rainfall and temperatures in West Africa**. Box-and-whisker plot of annual rainfall (*R*_*a*_; blue box plots and left hand scale) and annual mean temperatures (*T*_*a*_; red box plots and right hand scale) between 1973 and 2006 grouped for the West Sahel, Central Sahel, Guinean coast, and Cameroon as well as plotted in an ascending rainfall order.

Altogether 830 different sets of parameter settings of the LMM were forced by temperature and rainfall conditions derived from intermittent time series from 34 weather stations. The model was, therefore, subject to different climate conditions (cf. the varying temperature and rainfall values in Figure 2). The climates covered by the meteorological data set range from arid hot desert to tropical monsoon climates and therefore lead to various observed transmission levels of epidemic and endemic malaria. Fairly dry conditions in the Sahel, for example, resulted in the field in no malaria transmission at Diomandou Dieri (Senegal; 16°31'N; 14°39'W) [12]. In contrast, nearly continuous rainfall caused year-round transmission in Cameroon at Etoa (3°46'N; 11°29'E) [13]. The temperature range of this meteorological data set lie, for most stations, well above 20°C (cf. Figure 2) inhibiting the model validation for the lower malaria temperature limit of about 16°C [14].

#### Regional climate simulations

Driving the LMM with gridded data is needed for the validation of the simulated malaria spread in Africa. Due to the lack of gridded daily temperature and rainfall data from ground observations, data from a so-called regional climate model are used. Such a model is able to more accurately represent spatial variations of the atmosphere than so-called global atmosphere-ocean general circulation models.

Gridded LMM simulations for Africa were based on meteorological data from three ensemble runs between 1960 and 2000 from the REgional MOdel (REMO) [15]. REMO is a hydrostatic, limited area model using primitive equations, which are solved on 20 hybrid atmospheric levels [16]. At the lateral and lower boundaries REMO was nested into ECHAM5/MPI-OM (European Centre HAmburg Model, 5th generation/Max-Planck-Institute-Ocean Model) global coupled climate model simulations that were forced with the observed greenhouse gas increase [17-19]. The space-time resolution of REMO data used in the present study was 0.5° and daily values, respectively.

Expected systematic model errors in annual precipitation and in simulated temperatures were considerably reduced using bias correction, ensuring realistic data input for the malaria simulations [11]. The bias-corrected data are later used to drive gridded malaria runs in order to compare the original and new LMM versions.

#### Entomological and parasitological observations

In terms of malaria modelling, the entomological and parasitological data are of particular interest since malaria models have to undergo validation procedures. The present study was restricted to West Africa since this area was one of the focus regions of the IMPETUS project (*Integrated Approach to the Efficient Management of Scarce Water Resources in West Africa*) [20]. Numerous published malaria observations were extracted from the literature.

It was shown that population density is an important factor of malaria transmission [21,22]. In addition, crop irrigation strongly impacts malaria and its seasonality [23-26]. Also malaria and vector control such as the usage of impregnated bed nets are able to reduce transmission [27-31]. In the present study, field observations were only included for which a rural environment can be assumed from the information given in the publication (see Additional file 2 which includes also data from non-rural sites). Urban and irrigated areas and those when permanent streams influenced transmission were excluded (except for the locations in Cameroon, where this is nearly always the case). The few sites subject to vector control or other local or regional interventions were not used. This procedure ensured that other factors than climate, which influence malaria transmission, were excluded to the extent possible.

Entomological malaria field studies frequently sample biting mosquitoes on humans [32]. Standard measures include the so-called *Human Biting Rate *(*HBR*), which is the number of mosquito bites per human per time. However, only female mosquitoes with sporozoites in their salivary glands are able to infect humans. This fraction of the biting females is called *CircumSporozoite Protein Rate *(*CSPR*). Multiplying *HBR *with *CSPR *results in the so-called *Entomological Inoculation Rate *(*EIR*), which is defined as the number of infectious mosquito bites per human per time [33]. Only months revealing infectious mosquito bites (*EIR *values above zero) are usually used to define the malaria season at a certain location [34]. By contrast, parasitological malaria studies usually measure the *asexual parasite ratio *(*PR*) representing the proportion of the survey population, which is positive for the malaria parasite [35]. The literature was, therefore, reviewed in terms of malaria field studies (see Additional file 2).

Wherever applicable, observations were taken into account with regard to eleven different entomological and parasitological variables (these are: the *annual Human Biting Rate *(*HBR*_*a*_), the *annual Entomological Inoculation Rate *(*EIR*_*a*_), the *annual mean CircumSporozoite Protein Rate *(*CSPR*_*a*_), the *length*, *start*, and *end of the malaria season *(*Seas*, *SSeas*, and *ESeas*, respectively), the *length of the main malaria season *(*MSeas*; i.e. the number of months in which 75% of *EIR*_*a *_is recorded [36]), the *month of maximum transmission *(*XSeas*; i.e. the month with the largest entomological inoculation rate), the *annual mean*, *maximum*, and *minimum of the asexual parasite ratio *(*PR*_*a*_, *PR*_*max; a*_, and *PR*_*min, a*_, respectively). Included are the literature references as well as some basic features such as the name, geographical position, land-use of the study site, and the time period. Note that all these malaria variables can be computed from the LMM output.

### Definition of malaria seasonality

The definition of the malaria season is based on the *monthly Entomological Inoculation Rate *(*EIR*_*m*_), which is observed from field studies. The introduction of the malaria parasite into the LMM is assured by a constant influx of new infectious mosquitoes resulting in *EIR *values that might exceed those of low transmission areas. In order to compare 'truly' modelled *EIR *values with field observations, the artificial *EIR *values were removed via two separate LMM runs. The standard run results in a mixture of bites from the added infectious mosquitoes and those which were infected when they bit infectious humans in the simulation. The artificial *EIR *values were produced in a second run when only the added infectious mosquitoes were biting in the model, achieved by setting the *number of produced eggs per female mosquito *(#*E*_*p*_) to zero. Here, the oviposition of *Anopheles *females is prohibited and the mosquito population is therefore not able to grow. The subtraction of the *EIR *values of the second run from that of the standard run results finally in the elimination of artificial *EIR *values. Note that the same procedure was used with regard to the computation of the human biting rate (*HBR*).

In field studies, the malaria season is mostly determined on a monthly scale and is usually referred to months with *EIR*_*m *_values above zero [34]. For this reason, in the model the malaria season starts (*SSeas*) by definition in the first month with an *EIR*_*m *_value of at least 0.01 infectious bites per human. According to the formulation of the model, the arbitrary *EIR *value of 0.01 means that during a 30-day month at least one out of the 100 modelled humans is bitten by an infectious mosquito. In West Africa, the malaria season usually ends when the number of mosquitoes decreases at the end of the rainy season. Therefore, mosquito biting is reduced to such low numbers that the *EIR *values are reduced to zero indicating that transmission has ceased. Consequently, the last month during the transmission period defines the end of the malaria season (*ESeas*). Some individual years also reveal year-round or even no malaria transmission for certain locations. The length of the malaria season (*Seas*) is therefore the number of months with *EIR*_*m *_reaching at least 0.01 infectious bites. For each site or grid box additionally the length of the main transmission season (*MSeas*) is defined as the number of months in which 75% of *EIR*_*a *_is transmitted, an index which was used by Hay *et al*. [37]. Where possible the month with the maximum malaria transmission (*XSeas*) is identified as the month with the highest *EIR*_*m *_value.

The use of the transmission threshold of 0.01 infectious mosquito bites per human per month ensures the attainment of a reasonable transmission level in the model that can be compared with observations from the literature. However, the definition of the malaria season in the model might not be directly comparable to field studies since observations are subject to a certain detection limit. That is due to the fact that field experiments do not continuously measure biting rates (at best two times in each week of the field campaign) and that these field studies do not account for every human of the population of the study site.

### Definition of the validation

For the model validation, the LMM runs are driven by weather observations from meteorological stations in West Africa. The malaria model simulations are compared against observations from more than 200 sites using up to eleven different entomological and parasitological variables. The skill of the malaria simulations is measured by a problem-adapted skill score (see below). The performance of every particular set of parameter settings of the model is measured for each of the eleven malaria variables by this skill score (*SC*(*x*)). In addition, the performance of a set of parameter settings with regard to all malaria variables is quantified by the sum of the eleven skill scores (*SC*(*all*)). This approach enables the production of a ranking of different sets of parameter settings. Finally, the most suitable set of parameter settings in terms of the skill score determines the new LMM version.

The LMM was run with meteorological time series covering 1973 to 2006 discussed above. Therefore, each simulation at a particular station produced 34 annual values for every considered variable (e.g. *EIR*_*a*_). Since malaria conditions are different between rural and urban areas [21], only data from rural field sites were used for the LMM validation. On the other hand, entomological data are never measured continuously because of the amount of work required in practice [38]. For this reason, no single long time series of malaria observations exist. However, field observations of a subset of the nine malaria variables were frequently found in the area of the considered weather stations. Unfortunately, such malaria data has not been detected for all the used stations. For example, in the vicinity of Parakou (Benin) no field observations were extracted from the literature.

A further data issue that must be taken into account arises from the fact that the malaria field measurements were not performed at the locations of the weather stations. However, observation sites are available in the vicinity of the weather stations meaning that they are located in same climatic zones (Figure 1). In this context, it must be noted that precipitation can differ greatly between locations just few kilometres apart, but meteorological stations are much more coarsely distributed [39,40]. Also different environmental conditions (e.g. state of land surface) complicate the comparison between simulations and field observations. Shaman and Day [39] allude to the mismatch between the scales at which disease vectors respond to hydrologic variability and the scales at which hydrologic variability is actually monitored. For all these reasons, a reproduction of the field values of particular years, for example, of transmission rates is not expected by the LMM. In addition, it implies that paired annual correlations at single stations are not useful. Therefore, the usage of standard skill scores, used to determine the skill in weather forecasting [41], is not possible with the data sets that are available. In addition, statistical moments of the model and observational malaria data cannot be statistically meaningful tested due to the low sample size of the field observations. In order to overcome these issues, a subjective problem-adapted skill score is defined in the following section.

#### Definition of a skill score

A special validation procedure must be defined. It must be considered that simulations will not be able to reproduce single field observations. However, it is possible that the LMM simulates about the correct mean and variability of malaria variables, as observed in the field, since the weather station and the assigned field sites encounter similar climate conditions. In the following, location names refer to the name of the weather station, even if malaria observations from a nearby field site are discussed. The assignment of the weather stations to the malaria study sites is given in Additional file 2.

The evaluation claims that the simulated LMM values are comparable to field observations in reproducing the malaria-climate relationship across a range of malaria variables for the locations investigated in this paper. The validation procedure controls if, in general, the values of the model simulation agree with the field observations. Also the quantity of available observations is taken into account. The following six criteria define a problem-adapted scoring system (see also Table 2):

**Table 2:** Criteria in terms of the evaluation of LMM simulations

#	name	description	n_obs_	points	var
1	overlap	Any observation is included in the simulated range	≥1	+1	all variables
2	enclosure	Every observation is included in the simulated range	≥2	+1	all variables

3	median enclosure	The observed median is included in the simulated range	≥3	+1	*HBR*_*a *_*EIR*_*a *_*CSPR*_*a *_*Seas MSeas PR*_*a *_*PR*_*min,a *_*PR*_*max,a*_
4	median quartile enclosure	The observed median is located within the lower and upper quartile of the simulations	≥5	+1	*HBR*_*a *_*EIR*_*a *_*CSPR*_*a *_*Seas MSeas PR*_*a *_*PR*_*min,a *_*PR*_*max,a*_
5	penalty	The simulations exceed the one and a half time maximum of all field observations	≥1	-5	*HBR*_*a *_*EIR*_*a*_
6	frequency	I: The observations as well as the simulations show the same month maximum occurrence of the monthly entomological inoculation rate (*EIR*_*m*_).	≥3	+1	*SSeas ESeas XSeas*
		II: The majority of the observations and simulations show no or yearround transmission, respectively.			
		III: The observations and simulations reveal mostly no transmission.			
		IV: The month showing the most field observations of multiple years of *EIR*_*m *_is identical to the particular month resulting from the simulation.			
		*SSeas/ESeas: *Criterion I or II			
		*XSeas*: Criterion III or IV			

Criteria in terms of the evaluation of LMM simulations which are based on field observations in the area of synoptic stations. The malaria runs are rated separately for each station. Every fulfilled criterion increases the score of such a model run by one point. The sum of the achieved points at all stations and from the eleven entomological and parasitological variables finally add to the skill score of a particular LMM set of parameter settings (*SC*(all)). Columns: #: criteria number; name: short term; description: criterion description; n_obs_: lowest number of available observations needed to fulfil the criterion; points: assigned number of points; var: malaria variables for which the particular criterion are applied.

The evaluation of the model runs is performed by means of a *skill score *(*SC*), which considers the number of available observations for each of the eleven malaria variables. Stations with at least five observations contribute at maximum three or four points to the calculated skill score. Weather stations with fewer field measurements add one to three points to the skill score (cf. Table 2). No score is added when stations reveal no observations. The validation method also makes allowances for the uncertainty of the year-to-year variability. A proper analysis would only be possible for numerous available observations per station. In fact, an estimate of the frequency distribution of observed malaria parameters is not feasible from the few records available.

The computed skill score is based on the probability that observations, assigned to particular weather stations, fall into certain ranges of simulations (Table 2). It is expected that any observation is included in the range of the model simulations with regard to this station data (criterion 1: overlap). Of course, the model version performs better when every observation is enclosed in the simulated range (criterion 2: enclosure). Where there are at least three available records the observed median is also calculated. The confidence in the run increases when the observed median is contained in the range of the model simulations (criterion 3: median enclosure). The reliability of the median estimate increases for sites where five observations or more field measurements exist. Only for at least five available records the condition has to be met that the observed median falls within the range of the lower (25th percentile) and upper quartile (75th percentile) of the 34 simulated annual values (criterion 4: median quartile enclosure). For model integrations with different parameter settings, every fulfilled criterion at a weather station adds one point to the skill score of a particular malaria variable.

This rating system might favour model versions generating unrealistic high values and a strong interannual variation. This fact is countered by another criterion that eliminates unrealistically high entomological values (criterion 5: penalty). Five penalty points are applied to the skill score of *HBR*_*a *_and *EIR*_*a*_, when any simulated value exceeds one and a half times the maximum of all available field measurements (see Table 2). This threshold seems to be a reasonable measure for the restriction of simulated values. Sets of parameter settings leading to unrealistic high biting rates (*HBR*_*a *_and *EIR*_*a *_values) are rejected.

The third and fourth criteria are not calculable for three of the variables under certain circumstances (cf. Table 2): the start (*SSeas*) and end (*ESeas*) of the malaria season (for which the criteria are undefined in the cases of no transmission or year-round transmission), and the month of maximum transmission (*XSeas*) (for which they are undefined in the case of no transmission). For these variables, the maximum of the frequency distribution is compared instead (criterion 6: frequency). Regarding *SSeas *and *ESeas*, a model version is given an additional score of one point when both the observations as well as the simulations show the same month of maximum occurrence. A point is also gained when the majority of the observations and simulations show no or year-round transmission, respectively. For *XSeas*, a model version can achieve one additional point for a weather station either if both the observations and simulations reveal mostly no transmission or if the month showing the most field observations of multiple years of maximum *EIR*_*m *_values is identical to the particular month resulting from the simulation (e.g. if for a single station the strongest transmission values are both mostly observed and simulated for August).

**Figure 3:**
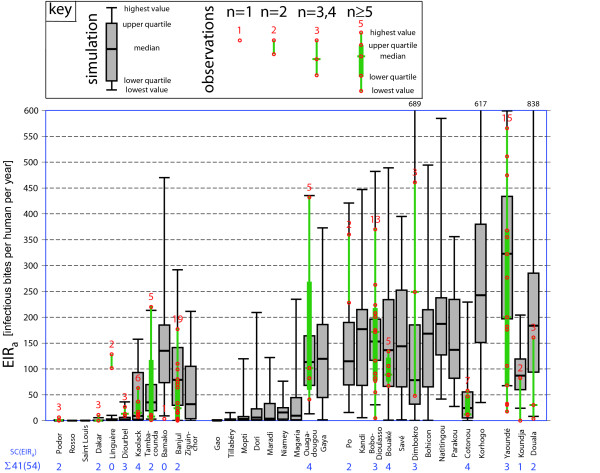
**Observed and simulated annual entomological inoculation rates**. Validation of LMM_2010 _simulations in terms of the annual entomological inoculation rate (*EIR*_*a*_) in the area of the 34 synoptic stations in West Africa used in this study. The simulated 34 annual *EIR*_*a *_values between 1973 and 2006 are illustrated as grey box-and-whisker plots (the numeric values of maxima beyond the scale of the ordinate are plotted on the upper abscissa). Field observations of *EIR*_*a *_(green lines and box plots) are either displayed as a vertical line (two available measurements), a vertical line with the median (three or four values), or as a box-and-whisker plot (≥ five data points). Each observation is furthermore inserted as a red circle and the number of observations is given above the entered observations (red digits). The skill score in terms of *EIRa *(*SC*(*EIR*_*a*_)), a measure of the performance of the simulations with regard to observed data, is denoted for every station as a blue digit. Stations are grouped as in Figure 2.

For each weather station, a skill score is determined for every set of parameter settings and for every one of the eleven entomological and parasitological variables (e.g. for Ouagadougou the skill score in terms of *EIR*_*a*_, i.e. *SC*(*EIR*_*a*_), is 4 for the LMM_2010_; see Figure 3). The skill scores from the weather stations add up to the skill score of a particular variable (*SC*(*x*); e.g. *SC*(*EIR*_*a*_) is 41 for the LMM_2010_; see Figure 3). Finally, the sum of all skill scores of a particular set of parameter settings is calculated (i.e. *SC*(*all*), which is 279 for the LMM_2010_).

**Figure 4:**
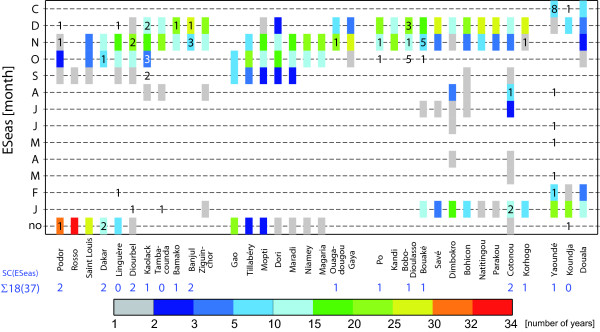
**Observed and Simulated values of the end of the malaria season**. Validation of LMM_2010 _simulations in terms of the end of the malaria season (*ESeas*) in the area of the 34 synoptic stations in West Africa as ordered for Figure 2. Each month is given a colour-coded rectangle representing the occasions for years between 1973 and 2006, when the malaria season finished in the model simulations. The simulated data (colour-filled rectangles) are compared to observed values (inserted as a digit). The frequency distribution (in numbers) regarding the simulated 34 values for 1973-2006 is given for each month. The frequencies of years with no ('no') and year-around ('C') transmission are also illustrated in the lowermost and topmost rows, respectively. The skill score in terms of *ESeas *(*SC*(*ESeas*)) is denoted for every station as a blue digit above the colour bar.

**Figure 5:**
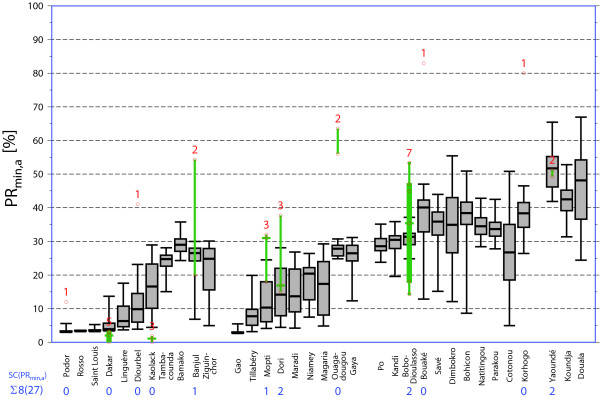
**Observed and simulated values of the annual minimum of the asexual parasite ratio**. Same as Figure 3 but for the annual minimum of the asexual parasite ratio (*PR*_*min, a*_).

For each particular station and each variable a certain number of points are achievable (e.g. 54 points can be reached in terms of *SC*(*EIRa*)). As stated above this optimum depends on the availability of field observations (see Table 2), which differs for each weather station and each malaria variable. The minimum number of possible points is zero for stations, with no available field observations (e.g. see Parakou in Figures 3, 4, and 5). At maximum four points can be achieved, when at least five observations are available. For example, numerous field observations are available for Bobo-Dioulasso (see Figure 3, 4, and 5). In case of *SSeas*, *ESeas*, and *XSeas *at maximum only three points can be achieved since only three criteria are applied for these variables (see Table 2).

## Results

In the following, undefined parameters of the LMM are calibrated by means of West African field observations. A two step approach is used in order to reduce the degrees of freedom of the model within each step. The performance of the model runs is measured by the problem-adapted scoring system. At the end, the set of parameter settings revealing the highest skill score is selected. Finally, the simulations of the LMM_2004 _and LMM_2010 _are compared to field observations of the eleven entomological and parasitological malaria variables.

### Calibration of the LMM

The majority of the model parameters were determined in the first part of this study [10]. However, some parameters were not allocatable due to a large spread in their published values or due to the lack of data. These undetermined values were two of the three parameters (*S *and *U*_2_) of the fuzzy distribution model. Here, *S *defines the most suitable 10-day accumulated rainfall conditions for mosquito breeding, meaning rainfall provides open water surfaces, which tend not to be flushed out by heavy rainfall. *U*_2 _defines the upper limit of suitable rainfall conditions above which all breeding habitats are flushed out. Note that *U*_1_, which is the lower limit of suitable rainfall conditions, was simply set to 0 mm since under prolonged dry conditions ephemeral water bodies will dry up [10].

In addition, the *number of produced eggs per female mosquito *(#*E*_*p*_), the *cap on the number of fertile mosquitoes *(*CAP*), as well as the *shift off relative to the dry season mosquito survival probability *(*p*_*d*↓_) are undetermined. Here, *p*_*d*↓ _is used to reduce vector survival during dry atmospheric conditions [10]. In the first part of this study [10], some parameter values were estimated from the range of published data and few were based on educated guesses, this might have resulted in some false estimation of these predefined parameters. However, the following calibration of the remaining parameters will largely compensate such inaccurate assessments.

As outlined above, the LMM calibration was undertaken for weather conditions at meteorological stations and included two general steps: (i) The initial experiment enabled a rough estimate of realistic parameter values and allowed the setting of two undetermined parameters. (ii) The second set of model runs permitted a final adjustment of the remaining model parameter settings. In order to simplify the calibration procedure, *p*_*d*↓ _was initially set to zero. Altogether three different settings were used for *U*_2 _(i.e. the upper limit of suitable rainfall conditions in terms of larval breeding of the fuzzy distribution model). Ahumada *et al*. [42] defined extreme rainfall as more than 255 mm of cumulative rainfall throughout a period of three days. Their model markedly reduces the mosquito population under excessive rainfall. According to a 10-day period, which is used by the fuzzy distribution model, this suggests a *U*_2 _value of about 500-1000 mm. In fact, *U*_2 _was either set to 500, 750, or 1000 mm.

#### First step

For the isolation of particular sets of parameter settings the remaining parameters (*S*, *U*_2_, #*E*_*p*_, and *CAP*) were simultaneously varied (*S*: 5, 10, 15, 20, and 30 mm; *U*_2_: 500, 750, and 1000 mm; #*E*_*p*_: 50, 75, 100, 125, and 150 eggs; *CAP*: 250, 500, 750, 1000, and 2000 fertile females). The various LMM simulation runs were compared to data from the eleven entomological and parasitological variables (see Additional file 2), these are: *HBR*_*a*_, *EIR*_*a*_, *CSPRa*, *SSeas*, *ESeas*, *Seas*, *PR*_*a*_, *PR*_*max, a*_, and *PR*_*min, a*_. The first 375 (originating from the combination of 5·3·5·5 settings) different LMM settings were ranked with regard to the skill score of all variables (*SC*(*all*)) and in terms of the two entomological variables *HBRa *and *EIRa *(i.e. *SC*(*HBR*_*a*_, *EIR*_*a*_) = *SC*(*HBR*_*a*_)+*SC*(*EIR*_*a*_)). Here, *SC*(*HBR*_*a*_, *EIR*_*a*_) is measuring the skill of the model simulation in terms of both *HBR*_*a *_and *EIR*_*a*_.

The ranking of sets of parameter settings with regard to *SC*(*HBR*_*a*_, *EIR*_*a*_) showed that *S *(i.e. the most suitable rainfall condition in terms of larval breeding of the fuzzy distribution model) affects mainly the spread of malaria in the northern part of the Sahel, for example, at various stations in Senegal [11]. In these dry areas the growth opportunity of the mosquito population is strongly suppressed by the fuzzy distribution model when *S *is set to high values. In this case, fewer suitable breeding sites are assumed in the model reducing the number of eggs entering and of larvae surviving the aquatic stages [10]. Obviously, *S *has to be set to relatively low values in order to keep malaria going in the northern Sahelian zone. However, too low *S *values seem to be unrealistic since the potential evaporation in tropical Africa usually exceeds several millimetres per day. The optimal 10-day rainfall of *S *is finally fixed to 10 mm since this value still enables the simulation of malaria in the northern part of the Sahel (see step 1.1 in Table 3). This analysis shows the clear need for the validation of the model under different rainfall conditions.

**Table 3:** Overview of calibration experiments

step	Parameter	area	*SC*	result
step 1	*p*_*d*↓_= 0	-	-	-
step 1.1	*S *∈ [5, 30]	northern Sahel	*SC(HBR_a_, EIR _a_)*	⇒ *S *: = 10 mm
step 1.2	*U*_*2 *_∈ [500, 1000]	West Africa	*SC(all)*	⇒ *U*_*2 *_: = 500 *mm*
step 1.3	#*E*_*p *_∈ [50, 150]	Sahel	*SC(HBR_a_, EIR_a_)*	⇒ #*E*_*p *_∈ [75, 125]
step 1.4	*CAP *∈ [250, 2000]	West Africa	*SC(all)*	⇒ *CAP *∈ [300, 900]

step 2	*CAP *∈ [300, 900]	West Africa	*SC(HBR_a_, EIR_a_)*	⇒ *CAP*: 400 fertile
	#*E*_*p *_∈ [70, 130]			females, #E_p _: = 120 eggs,
	*p*_*d*↓ _∈ [0, 10]			*p*_*d*↓ _: = -10%

Overview in terms of the evaluation of the performed calibration steps. Columns: step: step number; parameter: particular settings of the model parameter; area: area of interest; *SC*: applied skill score; result: result of the calibration step (for more details see text).

The evaluation of the performance of the 375 model versions also enabled a final setting of *U*_2 _since highest skill scores were exclusively generated by a value of 500 mm [11]. The lowest skill scores were attained by settings with high values of *CAP*, #*E*_*p*_, and *U*_2_, as well as low values of *S*, which apparently evoke large mosquito populations (see Additional file 3 and step 1.2 in Table 3).

A closer analysis of the data revealed that malaria transmission rates in the Sahel are fairly sensitive to the setting of #*E*_*p *_(i.e. the number of produced eggs per female mosquito) [11]. The median *HBRa *value rises, for example, at Linguère, Mopti, and Diourbel from less than 100 to several thousand bites per year, when #*E*_*p *_increases from 50 to 150 eggs. Unfortunately, only nine field observations of *HRB*_*a *_and *EIRa *are available north of 14°30'N. This fact impeded a proper determination or a further confinement of #*E*_*p*_. However, the LMM underestimates (overestimates) *EIR*_*a*_, when #*E*_*p *_is set to 50 (150) eggs (see Additional file 3 and step 1.3 in Table 3).

After confirming the parameter range of #*E*_*p *_(75-125 eggs) also *CAP *(i.e. the cap on the number of fertile mosquitoes) was more precisely determined (step 1.4 in Table 3). *CAP *is only of importance for comparatively large annual rainfall amounts. *CAP *markedly reduces the number of deposited eggs and hence biting rates under wet conditions. In fact, the reduction is markedly pronounced in the Sudanian zone, along the Guinean coast, and in Cameroon for low *CAP *values (≤750 fertile females). In contrast, large values of *CAP *(≥ 1000) cause fairly high numbers of non-infectious and infectious mosquito bites [11]. The ranking relative to *SC*(all) shows that LMM versions reveal a small skill when *CAP *is set to 250 fertile females [11].

#### Second step

The basis of the second iteration of the LMM calibration are the conclusions taken from the first step. For this reason, only the setting of *CAP*, #*E*_*p*_, and *p*_*d*↓ _needed to be varied. For the second set of runs, the #*E*_*p *_values were varied between 70 and 130 eggs (7 steps at increments of 10 eggs) and *CAP *(i.e. the cap on the number of fertile females) was set between 300 and 900 fertile females (13 steps at 50 fertile females). Five different values for *p*_*d*↓ _(i.e. the reduction of dry season mosquito survival) were in addition utilised (0.0, -2.5, -5.0, -7.5, and -10.0%). The second set of model runs included altogether 455 (originating from the combination of 7·13·5 settings) different model sets of parameter settings.

The second set of runs shows that low (high) #*E*_*p *_and high (low) *CAP *values produce the highest skill scores in terms of the eleven variables (*SC*(*all*)). As stated above this is because #*E*_*p *_and *CAP *tend to compensate each other at the more humid locations (see Additional file 3). Particularly notable is the fact that various model versions exhibited comparatively high skill scores. This fact makes a final objective setting of the remaining parameters difficult. For simplicity, the model version with the highest skill score in terms of *HBR*_*a *_and *EIR*_*a *_(*SC*(*HBR*_*a*_, *EIR*_*a*_)) was chosen.

The set of parameter settings with #*E*_*p *_= 120 eggs, *CAP *= 400 fertile females, and *p*_*d*↓ _= -10% produced the highest skill score in terms of *HBR*_*a *_and *EIR*_*a*_. A total of 78 from 106 possible points (73.6%) was reached by this model version. With regard to *SC*(*all*) altogether 279 from the 440 possible points were gained (Additional file 3).

The #*E*_*p *_value of 120 eggs is in the middle of observations (see Additional file One of Ermert *et al*. [10]). The *p*_*d*↓ _value of -10% enables the simulation of a realistic end of the malaria season. Malaria transmission stops one to two months earlier in the model when this *p*_*d*↓ _value is applied (cf. Figure 4). The correspondence with MARA (mapping Malaria Risk in Afrika project) maps [43] is improved by the earlier break of malaria transmission.

### Validation of the LMM_2010_

Based on weather conditions observed at meteorological stations, results of the LMM simulations using the final set of parameter settings (see Table 1) compare well to observed values of eight entomological variables. The new model version leads to the simulation of about the same *EIR*_*a *_values as observed (Figure 3; *SC*(*EIR*_*a*_) = 41(54)) in both epidemic and endemic malaria areas of West Africa. For example, the LMM achieves 15 from 17 achieveable points with regard to criterion 1 (enclosure), all points in terms of criterion 3 (median enclosure), and 6 out of 8 points regarding criterion 4 (median quartile enclosure). The performance of the model is lower in terms of criterion 2 (enclosure), which appears with criterion 4 (median quartile enclosure) to be the most rigorous criterion.

Low *EIR*_*a *_values are modelled under dry conditions in the Sahel under epidemic malaria conditions. The *EIR*_*a *_values are much higher for annual rainfall of about 1000 mm and again decrease as observed in the field data when the model is subject to higher annual rainfall (Figure 3). Within endemic malaria areas the LMM_2010 _simulation encompasses in most cases the observed values of *EIR*_*a*_. For some stations with numerous observations even the median values of *EIR*_*a *_are comparable, for example, in vicinity of Bobo-Dioulasso in Burkina Faso (13 observations) or in the area of Kaolack in Senegal (six observations). However, there are also some exceptions, for example, in vicinity of Barkedji in Senegal the simulated *EIR*_*a *_is much lower than two observations [44]. The high biting rates in this area are probably a result of special local environmental conditions. LeMasson *et al*. [44] and Molez *et al*. [45] conjectured that the presence of clay hollows, which collect water as soon as the rains start, caused a long persistence of temporary ponds and hence of malaria transmission. With regard to the annual human biting rate (*HBR*_*a*_) and consequently also for the annual mean of the circumsporozoite protein rate (*CSPR*_*a*_), similar statements are valid. There is a correspondence between the LMM_2010 _simulations of *HBR*_*a *_as well as *CSPR*_*a *_and observations from entomological studies (*SC*(*HBR*_*a*_) = 37(52); *SC*(*CSPR*_*a*_) = 33(55); cf. Additional file 4). At nearly every station at least one observation is enclosed in the simulation (criterion 1; see Table 4). In only two cases the observed median is not enclosed in the LMM simulations (criterion 3; Table 4).

**Table 4:** LMM_2004 _and LMM_2010 _performance

	criteria number (see Table 2)							criteria number (see Table 2)						
***SC(x)***	**1**	**2**	**3**	**4**	**5**	**6**	**LMM**_**2004**_	**1**	**2**	**3**	**4**	**5**	**6**	**LMM**_**2010**_

*SC(HBR_a_)*	7(16)	5(16)	5(13)	0(7)	-60	-	-43(52)	**14(16)**	**8(16)**	**11(13)**	**4(7)**	**0**	-	**37**(52)
*SC(CSPR_a_)*	6(18)	0(16)	2(14)	1(7)	-	-	9(55)	**14(18)**	**4(16)**	**12(14)**	**3(7)**	-	-	**33**(55)
*SC(EIR_a_)*	12(17)	7(*16*)	10(13)	1(8)	-50	-	-20(54)	**15(17)**	7*(16)*	**13(13)**	**6(8)**	**0**	-	**41**(54)

Σ	25(51)	12(48)	17(40)	2(22)	-110	-	-54(161)	**43(51)**	**19(48)**	**36(40)**	**13(22)**	**0**	-	**111(161)**

*SC(Seas)*	11(16)	**5(12)**	7(9)	4(5)	-	-	27(42)	**15(16)**	4(12)	**8(9)**	*4(5)*	-	-	**31**(42)
*SC(MSeas)*	**13(14)**	5(12)	4(7)	0*(4)*	-	-	22(37)	12(14)	**9(12)**	**7(7)**	*0(4)*	-	-	**28**(37)
*SC(XSeas)*	12(17)	4(14)	-	-	-	2*(10)*	18(41)	**15(17)**	**6(14)**	-	-	-	2*(10)*	**23**(41)
*SC(SSeas)*	11(17)	2(14)	-	-	-	3(10)	16(41)	**16(17)**	**7(14)**	-	-	-	**5(10)**	**28**(41)
*SC(ESeas)*	**15(16)**	3(*12*)	-	-	-	**4(9)**	**22**(37)	13(16)	*3(12)*	-	-	-	2(9)	18(37)

Σ	62(80)	19(64)	11(16)	4*(9)*	-	9*(29)*	105(198)	**71(80)**	**29(64)**	**15(16)**	*4(9)*	-	*9(29)*	**128(198)**

*SC(PR_a_)*	4(13)	1(9)	2(5)	*1(2)*	-	-	8(29)	**9(13)**	**3(9)**	**3(5)**	*1(2)*	-	-	**16**(29)
*SC(PR_max, a_)*	7(11)	2(7)	3(5)	*0(2)*	-	-	12(25)	**8(11)**	**4(7)**	**4(5)**	*0(2)*	-	-	**16**(25)
*SC(PR_min, a_)*	3(12)	*1*(*8*)	0(5)	*0(2)*	-	-	4(27)	**5(12)**	*1(8)*	**2(5)**	*0(2)*	-	-	**8**(27)

Σ	14(36)	4(24)	5(15)	*1(6)*	-	-	24(81)	**22(36)**	**8(24)**	**9(15)**	*1(6)*	-	-	40(81)

*SC(all)*	101(167)	35(136)	33(71)	7(37)	-110	9*(29)*	75(440)	**136(167)**	**56(136)**	**60(71)**	**18(37)**	**0**	*9(29)*	**279**(440)

Performance of LMM_2004 _and LMM_2010 _relative to entomological and parasitological field studies in West Africa. Digits are taken in **bold**, when either the LMM_2004 _or the LMM_2010 _performs better. Values in *italics *indicate no difference between the two model versions. The numbers in brackets refer to points that could be theoretically achieved (see text). Inserted are the scores of the LMM_2004 _and LMM_2010 _in terms of single criteria (see Table 2) as well as to their sum (columns: LMM_2004 _and LMM_2010_). Columns: *SC*(*x*): skill score with regard to variable *x*; 1: score regarding the first criterion (overlap); 2: enclosure; 3: median enclosure; 4: median quartile enclosure; 5: penalty; 6: frequency. Subtotals (Σ) are furthermore inserted in terms of the skill scores of entomological (*HBR*_*a*_, *CSPR*_*a*_, and *EIR*_*a*_), malaria seasonality (*Seas*, *MSeas*, *XSeas*, *SSeas*, and *ESeas*), as well as parasitological variables (*PR*_*a*_, *PR*_*max, a*_, and *PR*_*min, a*_). The comparison of the skill scores of the eleven malaria variables with regard to the performance of the LMM_2004 _and LMM_2010 _(including only values from columns: LMM_2004 _and LMM_2010_) reveals a *P *value of 0.0064 in terms of the Wilcoxon signed rank test.

Another result of the calibration is that the simulations capture the variability of malaria transmission. The interannual variability of *EIR*_*a*_, for example, is fairly large. For most stations the number of infectious mosquito bites fluctuates between values of less than 100 and several hundred. Unfortunately, long-term studies are rare and continuous observations from rural sites are only available from Ndiop for four years (Senegal; 13°41'N, 16°23'W). In this Sahelian village, malaria transmission varied in the mid of the 1990 s between seven and 63 infective bites per human per year [34]. For this area, simulated *EIR*_*a *_values range from almost zero to about 158 infectious bites per human per year. Note that the LMM simulation refers to the meteorological data from Kaolack, which is located about 55 km to the northeast of Ndiop.

The simulation of the malaria seasonality by the LMM_2010 _is consistent with epidemic and endemic field observations, despite the fact that a part of the field observations reveals considerably heterogeneous values. The simulated length of the season agrees well with the observations (*SC*(*Seas*) = 31(42)). Only in one out of 16 cases is there no overlap (criterion 1) with observations. In three out of the five cases with at least five field observations, the optimum scoring of four points is achieved (see Additional file 4). In general, the season length shortens with decreasing annual rainfall. Short malaria seasons and no malaria transmission are simulated for the Sahel and year-round transmission is found, for instance, in Cameroon. Also the length of the main transmission season corresponds roughly to the observations (*SC*(*MSeas*) = 28(37)). At only two of the 14 weather stations no overlappings (criterion 1) are found. Criterion 3 (median enclosure) is fulfilled for each of the seven cases (Table 4).

The skill score in terms of the maximum transmission month (*XSeaS*) gains 23 from the 41 achieveable points (*SC*(*XSeas*) = 23(41); cf. Additional file 4). The month of maximum transmission overlaps (criterion 1) between simulations and observations in 15 out of 17 cases and all observations are enclosed in the simulation range (criterion 2) in six out of 16 cases. The simulated month with the maximum transmission therefore corresponds frequently with that of the observations, especially in the Sahel. However, in terms of *XSeas*, the model simulations disagree in Cameroon, at the weather stations of Yaoundé and Koundja for areas with year-round endemic malaria transmission.

Both the start (*SSeas*) and end (*ESeas*) of the malaria season are realistically reproduced by the LMM_2010 _version (Figure 4; *SC*(*SSeas*) = 28(41); *SC*(*ESeas*) = 18(37)). With regard to *SSeas *the observations and simulations do not overlap only in one case (criterion 1; Table 4). In terms of *ESeas*, in 5 out of 10 cases the maximum number of observations and simulations are found in the same month (criterion 6; Table 4). For weather stations that observed no malaria transmission in most years, the model also simulates most frequently no transmission. The same is true for Yaoundé; in terms of year-round transmission (see Additional file 4). Observations that fall outside of simulations hardly differ more than one month.

The performance of the LMM_2010 _with regard to the annual mean (*PR*_*a*_), annual minimum (*PR*_*min, a*_) and annual maximum of the asexual parasite ratio (*PR*_*max, a*_) is somewhat mixed. The main reason for the relatively low skill scores is the heterogeneous parasitological observations that cannot be reproduced by the LMM (Figure 5 and Additional file 4). Measurements of the *PR*_*min, a*_, for example, exhibit a remarkable spread. Nine observations of *PR*_*min, a *_reveal higher values than 50% whereas 23 values are lower than this threshold. These differences suggest that some special factors are strongly affecting *PR *values. As a consequence, the LMM_2010 _reaches only eight from 27 possible points (Figure 5; Table 4). Due to the fact that there is a stronger year-to-year variability in the LMM_2010 _in terms of the simulation of *PR*_*a *_and *PR*_*max, a *_the model performs better with regard to these two parasitological variables (*SC*(*PR*_*a*_) = 16 (29); *SC*(*PRmax*;*a*) = 16 (25); Table 4; Additional file 4).

### LMM_2010 _versus LMM_2004_

**Figure 6:**
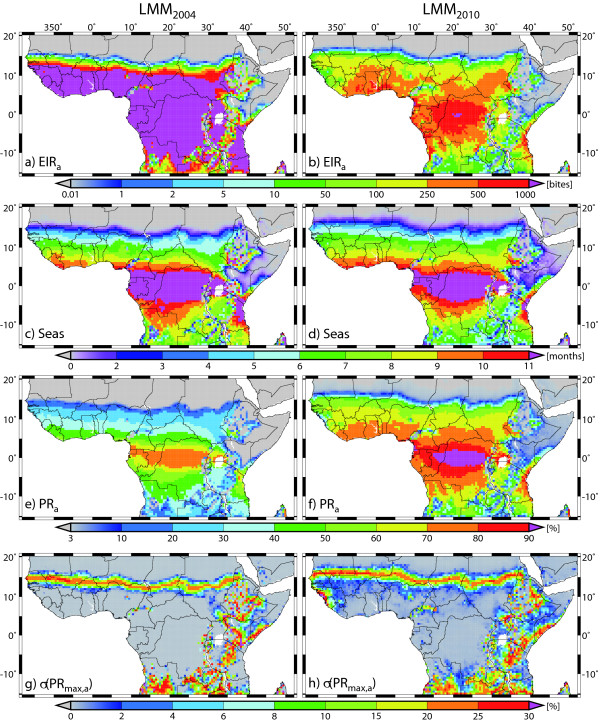
**Simulated malaria distribution**. LMM_2004 _and LMM_2010 _simulated present-day (1960-2000) malaria distribution and season length based on bias-corrected daily precipitation and temperature data from the regional climate model REMO. Displayed are (a & b) the annual entomological inoculation rate (*EIR*_*a*_), (c & d) the start month of the malaria season (*SSeas*), (e & f) the annual mean of the asexual parasite ratio (*PR*_*a*_), and (g & h) the standard deviation of the annual maximum of the parasite ratio (σ(*PR*_*max, a*_)).

In this section, the performance of the *LMM version of 2004 *(henceforth called LMM_2004_) is compared to that of the LMM_2010_. The LMM is validated using simulations that are based on time series driven by weather station observations (see Table 4). In addition, gridded LMM_2004 _and LMM_2010 _runs driven by the bias-corrected REMO data (see data sources) are compared (Figure 6).

The validation of the LMM_2004 _and LMM_2010 _runs based on weather station data from West Africa clearly demonstrates a significant improvement of the original model version (the *P *value is 0.0064; see Table 4). Except for *ESeas*, all skill scores are higher for the LMM_2010 _than for the LMM_2004_. Comparison of the LMM_2004 _simulations with field observations reveal two general features: (i) The LMM_2004 _fails to simulate malaria transmission in various malarious semi-arid regions and significantly overestimates the transmission in humid areas especially regions with endemic malaria territories. Note here that the LMM_2004 _was not designed to be used in endemic areas. (ii) Values of the parasite ratio, expressed in the three parasitological variables, are not optimal in the original model version. In terms of *ESeas*, the improvement of the model, especially in the Sahel, seems not to show up due to the lack of numerous observations of *ESeas *in this area. Nevertheless, the LMM_2010 _represents a significant step forward in the modelling of a weather-driven malaria transmission cycle.

The first feature (i) is a result of the simplified linear relationship between rainfall and the deposition of eggs by female mosquitoes in the LMM_2004_. In predominantly dry areas the model is not able to produce a reasonable size of the mosquito population. The LMM_2004 _hence fails to simulate malaria transmission in the northern part of the Sahel (Figure 6a &6c). By contrast, in the LMM_2010 _runs, malaria transmission is found as far north as about 18°N (Figure 6b &6d). This seems to be realistic since, for example, a definite malaria season was observed south of Agadez [46], which is located at about 17°N. Further the MARA project detected epidemic-prone areas up to 20°N [47].

In the second step, LMM runs were driven by gridded and bias-corrected REMO data for 1960-2000. Based on this input data the LMM_2010 _seems to reproduce a realistic picture with regard to transmission rates and the malaria seasonality for the whole of Africa including epidemic and endemic malaria areas (cf. Figure 6b &6d). This is in contrast to the LMM_2004 _which was originally only constructed for epidemic malaria territories [9]. This former model version simulates a tremendous number of mosquitoes and generates significantly too high *EIR*_*a *_values for humid areas such as the equatorial tropics (Figure 6a). *HBR*_*a *_partly exceeds 1,000,000 bites per annum in these endemic malaria areas [11]. Note that *HBR*_*a *_field values are usually much lower than 50,000 bites (see Additional file 2). However, the original model version also reveals deficiencies in terms of the spread of malaria within epidemic regions. The LMM_2004 _fails to simulate malarious areas north of about 16°N.

The infectiousness of mosquitoes is underestimated by the LMM_2004 _since values of the circumsporozoite protein rate (here *CSPR*_*a*_) are in general lower than 1% [11] resulting from the comparatively low mosquito survival of the Martens I scheme (see Figure 3 in [10]). In addition, the start of the malaria season is notably delayed in the LMM_2004 _simulations. The start of the season occurs about one to two months later under the original than under the new LMM version [11]. The strong growth of the mosquito population causes shorter main transmission seasons (*MSeas *values) in the LMM_2004 _runs than in that of the LMM_2010 _[11]. Moreover, a later occurrence of *XSeas *is found for the LMM_2004 _simulations.

Deficiencies of the LMM_2004 _are also found for the three parasitological variables. Almost the whole population clears the malaria parasite during the dry season due to the high *recovery rate *(*r*) of the LMM_2004 _(LMM_2004 _(LMM_2010_): *r *= 0.0284(0.005) day^-1^; see [10]). Such a characteristic is, however, not found in parasitological surveys (see Figure 5) and results in a very low skill in terms of *PR*_*min, a *_(see Table 4). The strong recovery from infection, moreover, underestimates the values of *PR*_*a *_and *PR*_*max, a *_[11]. In comparison to LMM_2010 _runs, parasite ratios are hence much lower in the LMM_2004 _simulations (see Figure 6e &6f).

One characteristic of malaria epidemic areas is the sudden and unexpected increase of the parasite ratio in certain years. Epidemic regions therefore should reveal a strong interannual variability of the annual maximum of the asexual parasite ratio (*PR*_*max, a*_; Figure 6g &6h) and in general low annual mean asexual parasite ratios (*PR*_*a*_; Figure 6e &6f). According to LMM simulations forced with gridded REMO data, epidemic areas are found along a strip within the Sahelian zone as well as for highland territories and fairly dry areas in East Africa, Zambia, and Angola (Figure 6g &6h). Most notable is the fact that in the LMM_2004 _runs the strip of high epidemic risk is detected about 2-4° to the south in comparison to that of the LMM_2010_. Note that the LMM_2010 _simulations seem to be more realistic in comparison to previous assessments of epidemic territories in the Sahel [47]. Differences between LMM_2004 _and LMM_2010 _simulations are also found outside of the Sahelian zone. Overall the LMM_2010 _seems to result in a stronger interannual variability of *PR*_*max, a*_.

## Discussion

The aim of the present study was the development of an improved weather-driven malaria model, which is able to simulate malaria transmission in both epidemic as well as endemic malaria areas. This section provides a detailed discussion with regard to various aspects of the present study. The present-day climate performance of the new model version are discussed relative to results of former studies and the model calibration is evaluated.

Comparison of LMM_2010 _runs with those performed by the original formulation of the model reveals a significant improvement (see Table 4) of the model performance both for epidemic as well as endemic malaria areas. In contrast to the LMM_2004_, the LMM_2010 _is able to reproduce the low transmission rates in the northern part of the Sahel. This enables, therefore, an improved detection of epidemic areas, in particular, in the Sahel. The LMM_2010 _validation also shows that the model can now also be applied for endemic malaria areas. The usage of the fuzzy distribution model enables the simulation of realistic sizes of the mosquito population under humid rainfall conditions resulting in reasonable transmission rates. Moreover, the lag in the malaria seasonality has disappeared in the new version of the LMM.

Various published malaria distribution maps [43,48] correspond well with the simulated spread of malaria by the LMM_2010_. However, in certain parts the simulated intensity of malaria transmission differs considerably from published malaria maps. The LMM_2010 _in general seems to predict higher transmission rates than satellite-derived predictions of *EIR*_*a *_from Rogers *et al*. [48]. The maps of transmission intensity provided by Gemperli *et al*. [49] are fairly patchy. In fact, the prediction from Gemperli *et al*. significantly suffers from the neglected interannual variability of malaria. Based on the few available *EIR*_*a *_observations it is difficult to judge which estimates are closer to reality. However, the validation of the LMM_2010 _under different climatic conditions provides evidence that the present study generated realistic biting rates and a reasonable interannual variability.

The calibration of the LMM was performed in West Africa for different atmospheric conditions of epidemic and endemic malaria regions. Realistic temperature and precipitation time series were reconstructed from various synoptic weather stations. The comparison with observations from eleven entomological and parasitological variables finally enabled the setting of undetermined model parameters.

The databases (including meteorological, entomological, and parasitological observations) for the LMM calibration are not optimal. There is a mismatch between the scales at which a disease vector responds to hydrologic variability and the scales at which hydrologic variability is actually observed. Systems should be developed that monitor hydrologic variability at scales corresponding to disease system ecologies [50]. In this study, the generation of realistic meteorological station time series enabled the comparison with atmospheric conditions from malaria field studies, which were not conducted directly at the weather stations. These sites therefore in any case exhibit a different temporal variability of rainfall and temperatures. This might be one reason, amongst other factors such as environmental conditions, why year-to-year comparisons between observation and simulation were weakly correlated at single locations. In order to circumvent this problem, the present study refrained from looking at paired annual correlations at single stations but applied a problem-adapted scoring system.

The required historical entomological and parasitological data are rarely available with sufficient coverage. Most locations show only one, two, or even no field measurements. It is therefore likely that a larger set of observations would have an impact on the result of the model calibration. Ideally, model simulations and malaria observations should be compared from year-to-year. However, this would require the simultaneous monitoring of long-term malaria data and meteorological measurements. Such long time series are available for the area of Ndiop/Senegal (S. Louvet, personal communication, 2007), but these data sets are not publicly available.

The close ranking of diverse model runs as well as the lack of sufficient validation data further restricted an objective formal fitting of the model. In fact, various steps of the calibration procedure were subjective. Due to high computational costs it was furthermore not possible to fit all remaining model parameters simultaneously. However, because various settings compensate each other it is likely that the final model formulation conforms as much as possible to reality.

The calibration and validation of the model should also be ideally not only restricted to West Africa. However, such an extension to, for example, East Africa was beyond the scope of this study. The Malaria Atlas Project intends to provide access to various malaria studies [51]. This might provide an efficient access to malaria data beyond that of West Africa. Such an extension would ideally include East African highlands and an estimation of the sporogonic temperature threshold.

This study was naturally not able to account for all processes involved in the spread of malaria. Some factors might be included in a future extension of the LMM. The simulation of the parasitological malaria variables by the LMM_2010 _is a simplification of real processes. The validation of the LMM_2010 _by means of parasitological measurements in West Africa revealed shortcomings of the new model version. Lower skill scores were achieved by the three parasitological variables when compared to the results from the eight entomological variables.

In addition to the lack of immunity, the LMM_2010 _does not account for other malaria factors such as chemoprophylaxis and human activities. However, this could be implemented by means of a variable parameter setting. Observations suggest a greater variability of the parasite ratio. At Bobo-Dioulasso, for example, the ten observed annual mean asexual parasite ratios (*PR*_*a*_) range between 29.1 and 77.5%. In contrast, the 34 annual values of the LMM_2010 _only span values between 50 and 70% [11].

Due to the lack of long-term observations, Kleinschmidt *et al*. [52] and Gemperli *et al*. [49] were forced to neglect the interannual variability of *PR*. This fact might again partly be responsible for their projected irregular *PR *pattern in West Africa. Their maps also show a sharp decrease of *PR*_*a *_north of about 15°N. In contrast to the LMM_2010 _runs and the Garki model simulations from Gemperli *et al*. [49], *PR*_*a *_is frequently lower than 50% south of 15°N. Only few regions exhibit higher *PR*_*a *_values than 70%, which are simulated by the LMM_2010_.

It should be pointed out here that climate is rarely the only important driver of malaria. Numerous other studies showed [21,22,53,54] that in particular human activities are crucial for the transmission and prevention of malaria across Africa. For example, the modification of the landscape by irrigation [55], forest clearing [56], or urbanization [57] can significantly alter malaria transmission. The present study assessed only the malaria risk for rural areas without the influence of permanent breeding places. The applicability of this analysis is therefore limited when permanent water bodies or urban centres are present. In principle, the calibration of the LMM_2010 _could also be performed for urban areas. However, such an undertaking seems to be hampered by the lower number of available observations (see Additional file 2).

## Conclusions

One of the most comprehensive studies to date in terms of gathering validation data and information from the literature for the development of a new version of an existing malaria model was conducted for West Africa. The first part of this study [10] provided new parameter settings of the LMM and changed some key processes in the model. The performance of numerous model versions were compared to malaria field observations from rural sites with no malaria measures and irrigation in order to maximise the climate-driven malaria impact. The comparison with observations from eleven entomological and parasitological variables finally enabled the specification of a final set of parameter settings of the model. Validation of the new LMM version in West Africa reveals that the simulations and malaria seasonality compare well with entomological field observations of epidemic and endemic malaria areas. The LMM_2010 _also demonstrates a fairly realistic simulation of the malaria spread as well as an improved detection of epidemic risk in Africa. Due to model limitations, the performance of the LMM_2010 _is somewhat weaker with regard to parasitological variables.

It is concluded that the LMM_2010 _represents a significant step forward in the modelling of a weather-driven malaria transmission cycle. In contrast to the original model, the application of the new model version is not only restricted to epidemic malaria regions but the usability is now extended to endemic malaria areas. Ermert [11] hence used the LMM_2010 _for the assessment of malaria risk under the influence of observed and projected climate change using regionalized climate projections for Africa. The LMM_2010 _simulated transmission rates were passed to the Garki model to form a hybrid malaria model, which is able to consider further aspects of the malaria disease such as age-dependent parasite ratios as well as the immune status of the population. In the near future, this hybrid model will be applied in a health early warning system, which is implemented by the QWeCI (Quantifiying Weather and Climate Impacts on Health in Developing Countries) project from the European Community's Seventh Framework Research Programme. Further work might enable the inclusion of some other processes involved in the spread of malaria into the LMM.

## List of abbreviations

DWD: German Weather Service; ECHAM5/MPI-OM: European Centre HAmburg Model, 5th generation/Max-Planck-Institute-Ocean Model; GSOD: Federal climate complex Global Surface Summary of Day version 7; LMM: Liverpool Malaria Model; LMM_2004_: Liverpool Malaria Model version of 2004; LMM_2010_: Liverpool Malaria Model version of 2010; MARA: Mapping Malaria Risk in Africa;

### List of symbols

#*E*_*p*_: number of produced eggs per female mosquito; *CAP*: cap on the number of fertile mosquitoes; *CSPR*: CircumSporozoite Protein Rate; *CSPR*_*a*_: annual mean CircumSporozoite Protein Rate; *EIR*: Entomological Inoculation Rate; *EIR*_*m*_: monthly Entomological Inoculation Rate; *EIR*_*a*_: annual Entomological Inoculation Rate; *ESeas*: End month of the malaria Season; *HBR*: human Biting Rate; *HBR*_*a*_: annual Human Biting Rate; *MSeas*: length of the Main malaria Season; *p*_*d*↓_: dry season mosquito survival probability shift off; *PR*: asexual Parasite Ratio; *PR*_*a*_: annual mean asexual Parasite Ratio; *PR*_*max, a*_: annual maximum of the asexual Parasite Ratio; *PR*_*min, a*_: annual minimum of the asexual Parasite Ratio; *r*: recovery rate; *R*: rainfall; *R*_*a*_: annual rainfall amount; *R*_Σ10*d*_: 10-day accumulated precipitation; *S*: most suitable rainfall condition according to the fuzzy distribution model; *SC*(*all*): skill score with regard to the used eleven malaria variables; *SC*(*x*): skill score with regard to malaria variable *x*; *Seas*: length of the malaria Season; *SSeas*: Start month of the malaria Season; *T*: temperature; *T*_*a*_: annual mean temperature; *U*_1_: lower threshold of unsuitable rainfall conditions with regard to the fuzzy distribution model; *U*_2_: upper threshold of unsuitable rainfall conditions regarding the fuzzy distribution model; *XSeas*: month of maXimum transmission, i.e. the month with the largest *EIR *value

## Competing interests

The authors declare that they have no competing interests.

## Authors' contributions

VE designed the study, undertook the literature review, proposed changes in the mathematical formulation of the LMM, ran the model, analysed the data from the model runs, and wrote the manuscript. AHF and APM contributed to the concept of the study. AHF supervised the PhD study of VE whose results formed the basis of the manuscript. APM was furthermore originally involved in the formulation of the LMM. AEJ contributed to the new design of LMM and provided the new model code. All authors read, suggested changes, and approved the final manuscript.

## Supplementary Material

Additional file 1 **Synoptic weather stations**. Information relative to synoptic weather stations from West Africa. The country, name, identifier, latitude and longitude positions, as well as the elevation of the meteorological stations are given. The LMM was driven by reconstructed temperature and precipitation time series (1973-2006) from these meteorological stations. Click here for file

Additional file 2 **Entomological and parasitological data**. Data with regard to entomological and parasitological observations from malaria field studies. Click here for file

Additional file 3 **Skill scores in terms of the LMM validation**. Ranks in terms of skill scores as computed for simulations of different LMM sets of parameter settings: (1) Top 10 and last 5 of 300 malaria runs from the first calibration step according to the skill score of the annual human biting and entomological inoculation rates (*SC*(*HBR*_*a*_;*EIR*_*a*_)). (2) Top 10 of 300 malaria runs from the first calibration step in terms of all eleven entomological and parasitological malaria variables (*SC*(*all*)). (3) Top 10 of 455 malaria runs from the second calibration step relative to *SC*(*HBR*_*a*_,*EIR*_*a*_). (4) Top 10 of 455 malaria runs from the second calibration step regarding *SC*(*all*). Click here for file

Additional file 4 **Observed and simulated entomological and parasitological values of the LMM_2010_**. Validation of LMM_2010 _simulations in terms of *HBR*_*a*_, *CSPR*_*a*_, *Seas*, *MSeas*, *XSeas*, *SSeas*, *PR*_*a*_, and *PR*_*max, a *_in the area of the 34 synoptic stations in West Africa as ordered in Figure 2. Click here for file
