# Supplementary material for: Development of a new version of the Liverpool Malaria Model. II. Calibration and validation for West Africa
Source: Malar J. 2011 Mar 16;10:62. doi: 10.1186/1475-2875-10-62 (PMC3070689; doi:10.1186/1475-2875-10-62)
Supplement: Additional file 1 — Synoptic weather stations. Information relative to synoptic weather stations from West Africa. The country, name, identifier, latitude and longitude positions, as well as the elevation of the meteorological stations are given. The LMM was driven by reconstructed temperature and precipitation time series (1973-2006) from these meteorological stations. [file 1475-2875-10-62-S1.PDF]

1 Synoptic weather stations

Information relative to synoptic weather stations from West Africa and Cameroon. The country, name, identifier, latitude and longitude positions, as well as the elevation of the meteorological stations are given. The LMM was driven by reconstructed temperature and precipitation time series (1973-2006) from these meteorological stations.

country	name	identifier	longitude	latitude	elevation [m]
Niger	Tillabéry	61036	1°27'E	14°12'N	210
Niger	Niamey	61052	2°10'E	13°29'N	227
Niger	Maradi	61080	7°05'E	13°28'N	373
Niger	Magaria	61091	8°56'E	12°59'N	403
Niger	Gaya	61099	3°27'E	11°53'N	203
Mali	Gao	61226	0°03'W	16°16'N	260
Mali	Mopti Barbe	61265	4°06'W	14°31'N	272
Mali	Bamako Senou	61291	7°57'W	12°32'N	381
Mauritania	Rosso	61489	15°49'W	16°30'N	6
Senegal	Saint-Louis	61600	16°27'W	16°03'N	4
Senegal	Podor	61612	14°58'W	16°39'N	7
Senegal	Linguère	61627	15°07'W	15°23'N	21
Senegal	Dakar Yoff	61641	17°30'W	14°44'N	24
Senegal	Diourbel	61666	16°14'W	14°39'N	9
Senegal	Kaolack	61679	16°04'W	14°08'N	7
Senegal	Tambacounda	61687	13°41'W	13°46'N	50
Senegal	Ziguinchor	61695	16°16'W	12°33'N	23
Gambia, The	Banjul Yundum	61701	16°48'W	13°21'N	33
Cameroon	Koundja Foumban	64893	10°45'E	5°39'N	1210
Cameroon	Douala	64910	9°44'E	4°00'N	9
Cameroon	Yaoundé	64950	11°31'E	3°50'N	760
Benin	Kandi	65306	2°56'E	11°08'N	292
Benin	Natitingou	65319	1°23'E	10°19'N	461
Benin	Parakou	65330	2°37'E	9°21'N	393
Benin	Savé	65335	2°29'E	8°02'N	200
Benin	Bohicon	65338	2°04'E	7°10'N	166
Benin	Cotonou	65344	2°23'E	6°21'N	9
Burkina Faso	Dori	65501	0°02'W	14°02'N	277
Burkina Faso	Ouagadougou	65503	1°31'W	12°21'N	306
Burkina Faso	Bobo-Dioulasso	65510	4°19'W	11°10'N	460
Burkina Faso	Po	65518	1°09'W	11°09'N	322
Burkina Faso	Korhogo	65536	5°37'W	9°25'N	381
Côte d'Ivoire	Bouaké	65555	5°04'W	7°44'N	376
Côte d'Ivoire	Dimbokro	65562	4°42'W	6°39'N	92
