# Supplementary material for: Development of a new version of the Liverpool Malaria Model. II. Calibration and validation for West Africa
Source: Malar J. 2011 Mar 16;10:62. doi: 10.1186/1475-2875-10-62 (PMC3070689; doi:10.1186/1475-2875-10-62)
Supplement: Additional file 2 — Entomological and parasitological data. Data with regard to entomological and parasitological observations from malaria field studies. [file 1475-2875-10-62-S2.PDF]

2 Entomological and parasitological observations

Data with regard to entomological and parasitological observations from malaria field studies.

Columns: country: country where the study was undertaken; place: location of the study site; long: longitude of the study site (-999.00: position is either unknown or was not seeked out); lat: latitude of the study site (-99.00: position is either unknown or was not seeked out); M1: month, when the study started; YYY1: year of the start of the study; M2: month, when the study ended; YYY2: year of the end of the study; $CSPR_a$: annual mean circumsporozoite protein rate; HBR_a: annual human biting rate ; EIR_a: annual entomological inoculation rate ; PR_a: annual mean asexual parasite ratio; $PR_{min,a}$: annual minimum asexual parasite ratio; $PR_{max,a}$: annual maximum asexual parasite ratio; *Seas*: length of the malaria season (in months); *MSeas*: length of the main malaria season (in months), that is the number of months in which 75% of EIR_a is recorded; *XSeas*: month of maximum transmission; *SSeas*: start month of the malaria season; *ESeas*: end month of the malaria season; WMO#: WMO station number indicating the assignment of the weather station to the malaria field site (see Additional file 1; -9999: no WMO station has been allocated to the data); U_b: land use classification as defined by Hay *et al.* [1]: R=rural, I=irrigated rice, U= urban area; $U_{\mathbf{a}}$: as U_b but after Hay *et al.* [2]: PU=peri-urban (population densities of 250-1,000 persons per km^2), R1=rural 1 (population densities of 100-250 persons per km^2), R2=rural 2 (population densities of $<$100 persons per km^2), U=urban (population densities of $>$1,000 persons per km^2); U_p: as U_b, but as taken from the particular reference: R=rural area, I=irrigation/permanent stream or river, U=urban area; notes: notes; ref: reference. The '-8' and '-9' denote data that were not available in the literature and that could not be checked due to limited access, respectively. Indices: [a]: the position of the study site was taken from Hay *et al.* [2]; [b]: the position of the study site was extracted from Hay *et al.* [1]; [c]: This value was calculated by using the relationship $EIR = HBR \cdot CSPR$; [d]: this value either was already considered or was observed before 1973; [e]: time to adult emergence; [ẽ]: *CSPR* was determined by the dissection of salivary glands of caught mosquitoes by microscope; [f]: *CSPR* was determined by the enzyme-linked immunosorbent assey (ELISA) technique; [g]: *HBR* was measured by human bait catches; [h]: *HBR* was measured by pyrethrum spray collections; [i]: the position of the study site was taken from http://www.heavens-above.com/countries.aspx; [ĩ]: *HBR* was measured by using light traps; [i]: the position of the study site was taken from http://www.heavens-above.com/countries.aspx; [j]: *HBR* was measured by using exit traps; [k]: this value was converted by using a multiplication factor of 3.57 [3]; [m]: the position of the study site was derived from a published map; [n]: in addition, no malaria transmission was observed during at least one malaria season; [r]: the position of the study site was found in the reference; [r̃]: this value is probably not representative (not all field sites could be considered); [s]: this value is similar to another value from a different reference regarding

the same field site and at about the same period; ^{y}: in addition, a year-round malaria transmission was observed during at least one malaria season;

Symbols: ⋆: children (the following numbers indicate age classes in years); ★: adults; ⧫: all ages.

country	place	long [°E]	lat [°N]	M1	YYY1	M2	YYY2	$CSPR_a$	HBR_a	EIR_a	PR_a	$PR_{min,a}$	$PR_{max,a}$	*Seas*	*MSeas*	*XSeas*	*SSeas*	*ESeas*	U_b [1]	U_a [2]	U_p	notes	ref	WMO#
Benin	Cotonou-Agabalilamè, Djègbadji, Kétonou	2.43	6.35^{i}	-8	-8	-8	-8	$0.27^{\tilde{e}f}$	4502^{gh}	12.10	-8.0	-8.0	-8.0	-8	-8	-8	-8	-8	-8	-8	R	rural area in vicinity of a lagoon	[4]	65344
Benin	Cotonou-Centre	2.43	6.35^{a}	-8	-8	-8	-8	$1.05^{\tilde{e}f}$	2768^{gh}	29.06	-8.0	-8.0	-8.0	-8	-8	-8	-8	-8	-8	U	U	becomes swampy during the rainy season	[4]	65344
Benin	Cotonou-Fiyégnon Donaten	2.43	6.35^{i}	-8	-8	-8	-8	$0.48^{\tilde{e}f}$	1064^{gh}	5.10	-8.0	-8.0	-8.0	-8	-8	-8	-8	-8	-8	-8	U	at the Atlantic coast	[4]	65344
Benin	Cotonou, Gbégamé quarter	2.45	6.36^{b}	1	1987	12	1987	$2.80^{\tilde{e}}$	1179^{gc}	33.00	-8.0	-8.0	-8.0	2	1	11	11	12	U	U	U	in the heart of Cotonou	[5]	65344
Benin	Cotonou-Ladji, Abomey-Calavi	2.35	6.45^{i}	-8	-8	-8	-8	$0.80^{\tilde{e}f}$	5870^{gh}	47.00	-8.0	-8.0	-8.0	-8	-8	-8	-8	-8	-8	-8	R	peri-urban area in vicinity of a lagoon	[4]	65344
Benin	Cotonou, Ladji quarter	2.43	6.38^{b}	1	1987	12	1987	$1.60^{\tilde{e}}$	3625^{gc}	58.00	-8.0	-8.0	-8.0	8	4	10	06	13	R	U	R	in the north of Cotonou quarter with lagoons	[5]	65344
Benin	Cotonou, Sainte-Rita Norde quarter	2.42	6.37^{b}	1	1987	12	1987	$1.40^{\tilde{e}}$	3357^{gc}	47.00	-8.0	-8.0	-8.0	6	3	08	07	13	R	U	R	-	[5]	65344
Benin	Ganvié	2.42	6.47^{b}	1	1993	12	1993	$0.43^{\tilde{e}}$	2555^{h}	11.00	-8.0	-8.0	-8.0	6	-8	-8	03	08	R	PU	R	built on a salt lake	[6,7]	65344
Benin	Ganvié, near lake Nokoué	2.42	6.42^{b}	1	1994	12	1994	0.36^{f}	2656^{g}	10.60	-8.0	-8.0	-8.0	7	-8	-8	-8	-8	R	U	R	built on a salt lake; reduced *EIR* after bed net usage	[7]	65344
Benin	Ganvié, near lake Nokoué	2.42	6.42^{b}	1	1995	12	1995	0.33^{f}	3540^{g}	11.70	-8.0	-8.0	-8.0	7	-8	-8	-8	-8	R	U	R	Ganvié is built on a salt lake; malaria transmission is reduced in the area with treated mosquito nets	[7]	65344
Burkina Faso	Bama	-4.42	11.38^{b}	1	1981	12	1981	-9.00	-9	175.20	-9.0	-9.0	-9.0	-9	3	-9	-9	-9	R	R2	R	-	[8]	65510
Burkina Faso	Baré	-4.10	11.08^{b}	1	1981	12	1981	-9.00	-9	91.24	-9.0	-9.0	-9.0	-9	3	-9	-9	-9	R	R1	R	-	[8]	65510
Burkina Faso	Baré	-4.10	11.08^{b}	-8	-8	-8	-8	-8.00	-8	-8.00	77.5	-8.0	-8.0	-8	-8	-8	-8	-8	-8	-8	R	*PR*: ⋆(5-14)	[9]	65510
Burkina Faso	Barkoumbilen and Barkoundouba	-1.23	12.67^{b}	3	1995	10	1995	-8.00^{f}	-8^{h}	>41.00	70.0	56.0	85.0	-8	3	09	-8	-8	R	R2	R	*PR*: ⋆(0-20); graphically derived	[10]	65503
Burkina Faso	Barkoumbilen and Barkoundouba	-1.23	12.67^{b}	8	1994	11	1994	-8.00^{f}	-8^{h}	>102.00	-8.0	-8.0	86.0	-8	3	09	-8	-8	R	R2	R	*PR*: ⋆(0-20); graphically derived	[10]	65503
Burkina Faso	Bella and Peul Djelgobé camps	-999.00	-99.00	6	1985	3	1986	$0.64^{\tilde{e}}$	-8^{g}	-8.00	50.7	37.7	71.5	-8	-8	-8	-8	-8	-8	-8	R	*PR*: ⋆(0.5-15)	[11]	65501
Burkina Faso	Bobo-Dioulasso	-4.30	11.20^{b}	11	1991	1	1993	$0.11^{\tilde{e}}$	2150^{g}	2.37	-8.0	-8.0	-8.0	-8	-8	-8	-8	-8	U	R1	U	-	[12]	65510
Burkina Faso	Bobo-Dioulasso, Colma-Nord quarter	-4.30	11.21^{b}	1	1985	12	1985	$0.19^{\tilde{e}}$	2421^{g}	4.60	29.1	18.0	47.6	2	2	-8	09	10	U/R	R1	R	*PR*: ⋆(0-15)	[13–15]	65510
Burkina Faso	Bobo-Dioulasso, Diaradougou quarter	-4.29	11.18^{b}	1	1985	12	1985	$0.19^{\tilde{e}d}$	74^{g}	0.14	6.8	4.6	10.3	1	1	09	09	09	U	R1	U	*PR*: ⋆(0-15)	[13–15]	65510
Burkina Faso	Bobo-Dioulasso, Dioulassoba quarter	-4.30	11.19^{b}	1	1985	12	1985	$0.19^{\tilde{e}d}$	289^{g}	0.55	10.9	5.4	13.9	1	1	09	09	09	U	R1	U	*PR*: ⋆(0-15)	[13–15]	65510
Burkina Faso	Bobo-Dioulasso, Sarfalao quarter	-4.30	11.20^{b}	6	1993	9	1993	$0.46^{\tilde{e}}$	870^{gc}	4.00	-8.0	-8.0	-8.0	-8	-8	-8	-8	-8	U	-8	U	-	[16]	65510
Burkina Faso	Bouloy	-0.42	14.70^{i}	6	1985	3	1986	$0.64^{\tilde{e}d}$	-8^{g}	-8.00	50.7^{d}	37.7^{d}	71.5^{d}	-8	-8	-8	-8	-8	-8	-8	R	*PR*: ⋆(0.5-15)	[11]	65501
Burkina Faso	Dandé	-4.57	11.58^{i}	1	1983	12	1983	-8.00	-8	-8.00	-8.0	-8.0	-8.0	4	3	09	07	10	R	R2	R	-	[17]	65510
Burkina Faso	Dandé	-4.57	11.58^{i}	1	1984	12	1984	-8.00	-8	-8.00	-8.0	-8.0	-8.0	4	2	10	07	10	R	R2	R	-	[17]	65510
Burkina Faso	Dandé and Tago	-4.55	11.59^{b}	1	1983	12	1984	$4.78^{\tilde{e}c}$	1380^{g}	55.00	-8.0	-8.0	-8.0	-8	-8	-8	-8	-8	R	R2	R	-	[17]	65510
Burkina Faso	Déou	-0.72	14.60^{i}	6	1985	3	1986	$0.64^{\tilde{e}d}$	-8^{g}	-8.00	26.6	16.9	45.5	-8	-8	-8	-8	-8	-8	-8	R	*PR*: ⋆(6-15)	[11]	65501
Burkina Faso	Desso	-4.28	11.35^{b}	1	1981	12	1981	-9.00	-9	208.04	-9.0	-9.0	-9.0	-9	3	-9	-9	-9	R	R2	R	-	[8]	65510
Burkina Faso	Gounghin Nord	-1.55	12.37^{b}	3	1984	12	1984	$0.00^{\tilde{e}}$	-8^{h}	0.00	-8.0	-8.0	11.8	0	0	-5	-5	-5	U	U	U	*PR*: ⋆(2-5)	[18,19]	65503

to be continued

Table 1 – continued

country	place	long [°E]	lat [°N]	M1	YYY1	M2	YYY2	$CSPR_a$	HBR_a	EIR_a	PR_a	$PR_{min,a}$	$PR_{max,a}$	*Seas*	*MSeas*	*XSeas*	*SSeas*	*ESeas*	U_b [1]	U_a [2]	U_p	notes	ref	WMO#
Burkina Faso	Karangasso	-4.63	11.22^{b}	1	1985	2	1986	$4.08^{\tilde{e}}$	6441^{gc}	262.80	63.0	35.4	82.5	6	3	09	06	11	R	R2	R	*PR*: $\star$(0-4)	[20,21]	65510
Burkina Faso	Karangasso	-4.63	11.22^{b}	1	1985	2	1986	$4.08^{\tilde{e}d}$	6441^{gdc}	262.80^{d}	59.5	47.0	75.0	6^{d}	3^{d}	09^{d}	06^{d}	11^{d}	R	R2	R	*PR*: $\star$(6-9)	[20,21]	65510
Burkina Faso	Karangasso	-4.63	11.22^{b}	1	1985	2	1986	$4.08^{\tilde{e}d}$	6441^{gdc}	262.80^{d}	65.3	53.3	78.9	6^{d}	3^{d}	09^{d}	06^{d}	11^{d}	R	R2	R	*PR*: $\star$(10-14)	[20,21]	65510
Burkina Faso	Karangasso, Koko suburb	-4.65	11.22^{b}	2	1985	2	1986	$2.41^{\tilde{e}}$	4805^{gc}	116.00	62.0	45.0	75.0	7	4	09	06	12	R	R2	R	*PR*: $\star$; graphically derived	[22,23]	65510
Burkina Faso	Karangasso, Koko suburb	-4.65	11.22^{b}	5	1985	4	1986	$2.55^{\tilde{e}s}$	4548^{ghs}	116.07^{s}	54.1	-8.0	-8.0	5^{s}	3^{s}	10	06^{d}	10^{s}	R	R2	R	-	[24]	65510
Burkina Faso	Karangasso, Koko suburb	-4.65	11.22^{b}	5	1986	4	1987	$4.56^{\tilde{e}}$	4913^{gh}	223.75	39.6	-8.0	-8.0	7	5	10	06	12	R	R2	R	-	[24]	65510
Burkina Faso	Karangasso, Massasso suburb	-4.64	11.21^{b}	2	1985	2	1986	$5.19^{\tilde{e}}$	7123^{gc}	370.00	62.0^{d}	45.0^{d}	75.0^{d}	7	4	09	06	12	R	R2	R	*PR*: graphically derived	[22,23]	65510
Burkina Faso	Karangasso, Massasso suburb	-4.64	11.21^{b}	5	1985	4	1986	$5.75^{\tilde{e}s}$	7012^{ghs}	403.33^{s}	54.1^{d}	-8.0	-8.0	8^{s}	3^{s}	10^{s}	05^{s}	12^{d}	R	R2	R	reduced *EIR* after impregnated bed net usage	[24]	65510
Burkina Faso	Kolel	-0.43	14.55^{i}	6	1985	3	1986	$0.64^{\tilde{e}d}$	-8^{g}	-8.00	50.7^{d}	37.7^{d}	71.5^{d}	-8	-8	-8	-8	-8	-8	-8	R	*PR*: $\star$(0.5-15)	[11]	65501
Burkina Faso	Kongodjan	-4.45	11.58^{b}	1	1983	12	1983	-8.00	-8	-8.00	59.9	40.5	69.2	7^{d}	3^{d}	08^{d}	06^{d}	12^{d}	R	R2	S	*PR*: $\star$(0.5-15); area: clay hollows/permanent pond	[25]	65510
Burkina Faso	Kongodjan	-4.45	11.58^{b}	1	1983	12	1983	-8.00	-8	-8.00	-8.0	-8.0	-8.0	7	3	08	06	12	R	R2	S	area: clay hollows/permanent pond	[17]	65510
Burkina Faso	Kongodjan	-4.45	11.58^{b}	1	1983	12	1984	$1.78^{\tilde{e}c}$	7480^{g}	133.00	-8.0	-8.0	-8.0	-8	-8	-8	-8	-8	R	R2	S	area: clay hollows/permanent pond	[17]	65510
Burkina Faso	Kongodjan	-4.45	11.58^{b}	1	1984	12	1984	-8.00	-8	-8.00	-8.0	-8.0	-8.0	7	5	10	06	12	R	R2	S	area: clay hollows/permanent	[17]	65510
Burkina Faso	Kongodjan	-4.45	11.58^{b}	1	1984	12	1984	-8.00	-8	-8.00	-8.0	9.1	-8.0	7^{d}	5^{d}	10^{d}	06^{d}	12^{d}	R	R2	S	*PR*: $\star$(0.5-15); area: clay hollows/permanent pond	[25]	65510
Burkina Faso	Koro	-4.20	11.15^{b}	1	1981	12	1981	-9.00	-9	171.60	-9.0	-9.0	-9.0	-9	3	-9	-9	-9	R	R1	R	-	[8]	65510
Burkina Faso	Koubri	-1.38	12.15^{b}	7	1984	12	1984	$7.10^{\tilde{e}}$	6082^{hc}	431.83	-8.0	-8.0	94.9	-8	3	08	-8	-8	R	R2	R	*PR*: $\star$(2-5)	[18,19]	65503
Burkina Faso	Ouagadougou, Kologh Naba suburb	-1.54	12.39^{b}	3	1984	12	1984	$1.47^{\tilde{e}}$	1359^{hc}	19.98	-8.0	-8.0	22.1	6	3	10	05	10	U	U	U	*PR*: $\star$(2-5)	[18,19]	65503
Burkina Faso	Ouagadougou, Nongremassm suburb	-1.51	12.40^{b}	3	1984	12	1984	$0.83^{\tilde{e}}$	920^{hc}	7.64	-8.0	-8.0	31.5	5	3	10	05	12	U	U	U	*PR*: $\star$(2-5)	[18,19]	65503
Burkina Faso	Ouagadougou, Saint Camille suburb	-1.52	12.36^{b}	3	1984	12	1984	$1.14^{\tilde{e}}$	399^{hc}	5.58	-8.0	-8.0	19.7	2	1	08	08	10	U	U	U	*PR*: $\star$(2-5)	[18,19]	65503
Burkina Faso	Ouagadougou, Saint Léon suburb	-1.52	12.37^{b}	6	1984	12	1984	$0.00^{\tilde{e}}$	-8^{h}	0.00	-8.0	-8.0	2.9	0	0	-5	-5	-5	U	U	U	*PR*: $\star$(2-5)	[18,19]	65503
Burkina Faso	Ouagadougou, Tanghin suburb	-1.53	12.40^{m}	8	1984	9	1984	-8.00	-8	-8.00	-8.0	-8.0	23.6	-8	-8	-8	-8	-8	-8	-8	U	*PR*: $\star$(2-5)	[19]	65503
Burkina Faso	Oursi	-0.45	14.68^{i}	6	1985	3	1986	$0.64^{\tilde{e}d}$	-8^{g}	-8.00	26.6^{d}	16.9^{d}	45.5^{d}	-8	-8	-8	-8	-8	-8	-8	R	*PR*: $\star$(6-15)	[11]	65501
Burkina Faso	Pabré	-1.58	12.50^{b}	7	1984	12	1984	$6.07^{\tilde{e}}$	1673^{hc}	101.58	-8.0	-8.0	79.6	-8	3	10	-8	-8	R	PU	R	*PR*: $\star$(2-5)	[18,19]	65503
Burkina Faso	six villages, 30 km N/NW of Ouagadougou	-999.00	-99.00	4	2003	10	2003	-8.00	-8	-8.00	-8.0	-8.0	-8.0	-8	-8	-8	04	-8	-8	-8	R	*PR*: $\star$(1-14)	[26]	65503
Burkina Faso	six villages, 30 km N/NW of Ouagadougou	-999.00	-99.00	6	2002	11	2002	-8.00	-8	-8.00	73.4	63.5	85.0	6	3	09	06	11	-8	-8	R	*PR*: $\star$(1-14)	[26]	65503
Burkina Faso	Soumosso	-4.05	11.02^{b}	1	1981	12	1981	-9.00	-9	200.75	-9.0	-9.0	-9.0	-9	3	-9	-9	-9	R	R2	R	-	[8]	65510
Burkina Faso	Tago	-4.38	11.67^{b}	1	1983	12	1983	-8.00	-8	-8.00	-8.0	-8.0	-8.0	6	3	08^{d}	06	10	R	R2	R	-	[17]	65510
Burkina Faso	Tago	-4.38	11.67^{b}	1	1983	12	1983	$-8.00^{\tilde{e}}$	-8^{g}	82.00	50.7	30.4	71.1	6^{d}	3^{d}	08	06^{d}	10^{d}	R	R2	R	*PR*: $\star$(0.5-15)	[25]	65510
Burkina Faso	Tago	-4.38	11.67^{b}	1	1984	12	1984	-8.00	-8	-8.00	47.6	14.3	77.6	6	3	08	06	10	R	R2	R	*PR*: $\star$(0.5-15)	[25]	65510
Burkina Faso	Tin Edjar	-0.68	14.69^{m}	6	1985	3	1986	$0.64^{\tilde{e}d}$	-8^{g}	-8.00	33.2	15.0	50.0	-8	-8	-8	-8	-8	-8	-8	R	*PR*: $\star$(0.5-15)	[11]	65501
Burkina Faso	Toukoro	-4.25	11.43^{b}	1	1981	12	1981	-9.00	-9	76.65	-9.0	-9.0	-9.0	-9	3	-9	-9	-9	R	R2	R	-	[8]	65510
Burkina Faso	VK1	-4.41	11.35^{b}	1	1981	12	1981	-9.00	-9	0.00	-9.0	-9.0	-9.0	-9	3	-9	-9	-9	I	R2	I	-	[8]	65510

to be continued

Table 1 – continued

country	place	long [°E]	lat [°N]	M1	YYY1	M2	YYY2	$CSPR_a$	HBR_a	EIR_a	PR_a	$PR_{min,a}$	$PR_{max,a}$	*Seas*	*MSeas*	*XSeas*	*SSeas*	*ESeas*	U_b [1]	U_a [2]	U_p	notes	ref	WMO#
Burkina Faso	VK2	-4.41	11.37[b]	1	1981	12	1981	-9.00	-9	21.90	-9.0	-9.0	-9.0	-9	3	-9	-9	-9	I	R2	I	-	[8]	65510
Burkina Faso	VK3	-4.41	11.38[b]	1	1981	12	1981	-9.00	-9	62.10	-9.0	-9.0	-9.0	-9	3	-9	-9	-9	I	R2	I	-	[8]	65510
Burkina Faso	VK4	-4.42	11.37[b]	1	1981	12	1981	-9.00	-9	20.10	-9.0	-9.0	-9.0	-9	3	-9	-9	-9	I	R2	I	-	[8]	65510
Burkina Faso	VK4	-4.42	11.37[b]	1	1985	2	1986	-8.00	-8	-8.00	27.4	16.0	35.7	6[d]	4	11	05[d]	12[d]	I	R2	I	*PR*: ⋆(0-4)	[20,21]	65510
Burkina Faso	VK4	-4.42	11.37[b]	1	1985	2	1986	-8.00	-8	-8.00	33.7	23.9	46.1	6[d]	4[d]	11[d]	05[d]	12[d]	I	R2	I	*PR*: ⋆(10-14)	[20,21]	65510
Burkina Faso	VK4	-4.42	11.37[b]	1	1985	2	1986	-8.00	-8	-8.00	41.1	29.4	58.0	6[d]	4[d]	11[d]	05[d]	12[d]	I	R2	I	*PR*: ⋆(5-9)	[20,21]	65510
Burkina Faso	VK4	-4.42	11.37[b]	2	1984	12	1984	0.39[c]	14000	50.00	34.0	25.0	45.0	6	4	10	06	11	I	R2	I	*PR*: ⋆, graphically derived	[17,23]	65510
Burkina Faso	VK4	-4.42	11.37[b]	5	1985	4	1986	0.22[ẽc]	25490[g]	54.90	-8.0	-8.0	-8.0	6	-8	-8	05	12	I	R2	I	reduced *EIR* after bed net usage	[27]	65510
Burkina Faso	VK5	-4.43	11.38[b]	1	1981	12	1981	-9.00	-9	36.50	-9.0	-9.0	-9.0	-9	3	-9	-9	-9	I	R2	I	-	[8]	65510
Burkina Faso	VK6	-4.44	11.37[b]	1	1981	12	1981	-9.00	-9	54.80	-9.0	-9.0	-9.0	-9	3	-9	-9	-9	I	R2	I	-	[8]	65510
Burkina Faso	VK6	-4.44	11.37[b]	1	1983	12	1983	-8.00	-8	-8.00	-8.0	-8.0	-8.0	4	3	06	06	09	I	R2	I	-	[17]	65510
Burkina Faso	VK6	-4.44	11.37[b]	1	1983	12	1984	0.43[ẽc]	13900[g]	60.00	-8.0	-8.0	-8.0	-8	-8	-8	-8	-8	I	R2	I	-	[17]	65510
Burkina Faso	VK6	-4.44	11.37[b]	1	1984	12	1984	-8.00	-8	-8.00	-8.0	-8.0	-8.0	7	2	07	05	11	I	R2	I	-	[17]	65510
Burkina Faso	Zagtouli	-1.63	12.33[b]	7	1984	12	1984	8.14[ẽ]	1009[hc]	82.11	-8.0	-8.0	57.6	-8	2	08	-8	-8	R/I	U	R	*PR*: ⋆(2-5)	[18,19]	65503
Cameroon	Bondi	12.19	3.86[m]	-8	1998	-8	2000	-8.00	-8	-8.00	50.0	49.3	50.7	-8	-8	-8	-8	-8	-8	-8	R	area: degraded forest; *PR*: ◆(0.8-77); position from [28]	[29]	64950
Cameroon	Ebogo	11.47	3.40[b]	4	1991	3	1992	0.84[ẽ]	38189[g]	355.00	-8.0	-8.0	-8.0	12	8	03	-1	-1	R	R2	R/S	area: water drainage	[30]	64950
Cameroon	Ebolakounou	12.13	3.93[a]	6	1997	5	1998	4.40[ẽc]	402[g]	17.70	61.8	-8.0	-8.0	2	2	05	05	06	-8	R2	R	area: forested; Seas: perennial transmission, but partly below the detection limit; *PR*: ⋆(0-15)	[28,31]	64950
Cameroon	Edea, Bilalang suburb	10.13	3.80[b]	1	1990	12	1990	1.20[ẽ]	317[gc]	3.80	-8.0	-8.0	-8.0	-8	-8	-8	-8	-8	U	R2	R	Seas: few mosquito catches	[32]	64910
Cameroon	Edea, Pongo suburb	10.13	3.80[b]	1	1990	12	1990	8.24[ẽc]	368[g]	30.20	-8.0	-8.0	-8.0	-8	-8	-8	-8	-8	U	R2	R	area: river presence; *HBR*: large spatial differences; Seas: few mosquito catches	[32,33]	64910
Cameroon	Etoa	11.48	3.77[b]	2	1996	5	1996	4.10[ẽc]	11571[g]	474.50	-8.0	-8.0	-8.0	-8	-8	-8	-8	-8	R	R2	R/S	area: river irrigation; too short study	[34]	64950
Cameroon	Etoa	11.48	3.77[b]	9	1994	2	1995	4.00[ẽ]	12775[gc]	511.00	54.7	-8.0	-8.0	12	-8	-8	-1	-1	R	R2	R/S	area: river irrigation, rainforest belt; too short study; *PR*: ⋆(1-15)	[35]	64950
Cameroon	Koundou	12.12	3.90[a]	6	1997	5	1998	3.80[ẽc]	4636[g]	176.10	69.5	-8.0	-8.0	6	3	05	03	08	-8	R2	R	area: degraded forest; Seas: perennial transmission, but partly below the detection limit; *PR*: ⋆(0-15)	[28,31]	64950
Cameroon	Mbalmayo	11.43	3.50[r]	2	2000	6	2001	3.60[f]	3588[ghc]	129.00	-8.0	-8.0	-8.0	12	-8	-8	-1	-1	-8	-8	U	area: river presence	[36]	64950
Cameroon	Mbébé	11.00	4.15[b]	4	1989	3	1990	1.94[c]	10330[g]	200.00	72.0	-8.0	-8.0	12	4	02	-1	-1	R	R2	R/S	area: river breeding; reduced *EIR* after impregnated bed net usage	[37]	64950
Cameroon	Mbébé	11.00	4.15[b]	4	1989	12	1989	1.23[s]	¿5041gs	¿62.00s	-8.0	-8.0	-8.0	-8	-8	-8	-8	-8	R	R2	R/S	area: river breeding	[37]	64950
Cameroon	Mengang	12.05	3.88[i]	-8	1998	-8	2000	-8.00	-8	-8.00	70.0	51.2	81.8	-8	-8	-8	-8	-8	-8	-8	R	area: degraded forest; *PR*: ◆(0.8-77)	[29]	64950
Cameroon	Mutengene, Molyko, Likoko, Vasingi	9.30	4.08[a]	06/11	1998/ 1999	09/02	1998/ 2000	6.97[ẽf]	2310[ghc]	161.00	71.4	-8.0	-8.0	-8	-8	-8	-8	-8	-8	R1	R	area: Mount Cameroon, RR_a: up to 11,000 mm; no transmission at 1200 m	[38]	64910
Cameroon	Nditam	11.26	5.36[b]	5	1995	3	1996	9.40[ẽ]	876[g]	82.34[c]	-8.0	-8.0	-8.0	12	2	09	-1	-1	R	R2	R	area: in secondary forest/near savannah	[39]	64893
Cameroon	Ngoumé	11.40	5.48[i]	5	1995	3	1996	-8.00[ẽ]	0[g]	0.00	-8.0	-8.0	-8.0	0	0	-5	-5	-5	-8	-8	R	area: in ancient forest	[39]	64893
Cameroon	Nsimalen, Ekoko	12.12	3.82[b]	4	1991	3	1992	1.16[ẽ]	8724[g]	106.00	-8.0	-8.0	-8.0	11	6	15	07	17	R	R2	R/S	area: river breeding, deforested due to airport construction	[40]	64950
Cameroon	Nsimalen, Nkol Mefou	11.57	3.70[b]	4	1991	3	1992	2.35[ẽ]	2920[g]	68.00	-8.0	-8.0	-8.0	8	6	13	08	15	R	R2	R/S	area: river breeding, forest	[40]	64950
Cameroon	Olama	11.30	3.40[r]	2	2000	6	2001	2.18[f]	14773[ghc]	322.00	-8.0	-8.0	-8.0	12	-8	-8	-1	-1	-8	-8	R/S	area: river presence	[36]	64950
Cameroon	Sanaga river villages	11.00	4.15[b]	4	1989	3	1990	1.86[ẽ]	10303[g]	182.10	-8.0	-8.0	-8.0	12	6	03	-1	-1	R	-8	R/S	area: forested, river breeding (December-June)	[41]	64950
Cameroon	Simbock	11.48	3.82[a]	2	1999	4	1999	4.80[ẽf]	-8	-8.00	-8.0	-8.0	-8.0	-8	-8	-8	-8	-8	-8	-8	R/S	area: forested	[42]	64950
Cameroon	Simbock, block 6	11.50	3.83[a]	10	1998	9	1999	2.71[f]	10214[gc]	276.80	-8.0	-8.0	-8.0	12	6	01	-1	-1	-8	R1	R/S	area: permanent swamp	[43]	64950
Cameroon	Simbock, block 6	11.50	3.83[a]	10	1999	9	2000	2.71[fd]	13576[gc]	367.90	-8.0	-8.0	-8.0	12	7	05	-1	-1	-8	R1	R/S	area: permanent swamp	[43]	64950
Cameroon	Simbok	11.47	3.82[a]	9	1994	2	1995	2.92[ẽc]	19345[g]	565.75	60.6	-8.0	-8.0	12	-8	-8	-1	-1	-8	R1	R/S	area: irrigated fields, rainforest; too short study; *PR*: ⋆(1-15)	[35]	64950

to be continued

Table 1 – continued

country	place	long [°E]	lat [°N]	M1	YYY1	M2	YYY2	$CSPR_a$	HBR_a	EIR_a	PR_a	$PR_{min,a}$	$PR_{max,a}$	*Seas*	*MSeas*	*XSeas*	*SSeas*	*ESeas*	U_b [1]	U_a [2]	U_p	notes	ref	WMO#
Cameroon	Yaoundé, Dakar quarter	11.52	3.87[i]	1	2000	12	2000	2.50[ẽ]	1360[gc]	34.00	-8.0	-8.0	-8.0	-8	-8	-8	-8	-8	-8	-8	U	area: breeding along river	[44]	64950
Cameroon	Yaoundé, Dakar quarter	11.52	3.87[i]	7	1999	5	2000	-8.00	-8	-8.00	34.0	25.0	40.0	-8	-8	-8	-8	-8	-8	-8	U	area: breeding along river	[44]	64950
Cameroon	Yaoundé, Essos	11.00	3.00[b]	6	1989	2	1990	-8.00	-8	-8.00	37.5	14.3	50.5	-8	-8	-8	-8	-8	-9	-9	U	area: near marshy shallows; *PR*: ⋆(0-15)	[45]	64950
Cameroon	Yaoundé, Essos suburb	11.00	3.00[b]	3	1989	2	1990	20.31[ẽc]	64[g]	13.00	-8.0	-8.0	-8.0	-8	-8	-8	-8	-8	U	R2	U	area: marsh breeding; *HBR* value in the text is different to that given in Tab. 1	[33]	64950
Cameroon	Yaoundé, Messa quarter	11.52	3.87[i]	10	1990	1	1993	-8.00	-8	-8.00	37.1	-8.0	-8.0	8	-8	-8	-8	-8	-8	-8	U	-	[46]	64950
Cameroon	Yaoundé, Nkol Bikok quarter	11.52	3.87[b]	3	1989	2	1990	5.00[ẽ]	285[g]	14.24	-8.0	-8.0	-8.0	1	1	05	05	05	U	-8	U	few mosquito catches	[47]	64950
Cameroon	Yaoundé, Nkol Bisson	11.52	3.87[i]	3	1989	2	1990	6.78[ẽc]	484[g]	32.80	-8.0	-8.0	-8.0	-8	-8	01	-8	-8	-8	-8	R/S	area: lake breeding; *HBR* in text differs from Tab. 1	[33]	64950
Cameroon	Yaoundé, Nkol Bisson	11.52	3.87[i]	3	1989	3	1990	1.67[ẽ]	1814[g]	30.30	-8.0	-8.0	-8.0	4	3	-8	01/06	02/08	R	-8	R/S	area: near pool; few mosquito catches	[47]	64950
Cameroon	Yaoundé, Obili district	11.52	3.87[i]	10	1989	7	1990	-8.00	-8	-8.00	27.8	21.1	45.5	-8	-8	-8	-8	-8	-8	U	U	area: fish breeding ponds; *PR*: ⋆(0-15)	[45]	64950
Cameroon	Yaoundé, Obili district	11.52	3.87[i]	-9	-9	-9	-9	-9.00	-9	3.00	-9.0	-9.0	-9.0	-9	-9	-9	-9	-9	-8	U	U	-	[48]	64950
Congo	Linzolo	15.11	-4.41[b]	10	1981	1	1984	2.20[ẽ]	11204[gc]	246.10	-8.0	-8.0	-8.0	12	-8	-8	-1	-1	R	R1	R	-	[49]	-9999
Congo	Linzolo	15.11	-4.41[b]	11	1981	5	1985	-8.00	-8	-8.00	79.1	78.8	80.9	-8	-8	-8	-8	-8	R	R1	R	-	[50]	-9999
Côte d'Ivoire	Alloukoukro	-5.08	7.80[b]	1	1991	12	1991	3.15[ẽ]	8469[gc]	266.50	-8.0	-8.0	-8.0	12	5	09	-1	-1	R	PU	S	-	[51]	65555
Côte d'Ivoire	Alloukoukro	-5.08	7.80[b]	1	1992	12	1992	3.50[ẽ]	5612[gc]	196.50	-8.0	-8.0	-8.0	12	5	09	-1	-1	R	PU	S	-	[51]	65555
Côte d'Ivoire	Bouaké, Dar-és-Salam	-5.03	7.68[i]	1	1991	12	1991	0.90[ẽ]	4889[gc]	44.00	-8.0	-8.0	-8.0	7	5	05	04	11	-8	-8	I	no transmission in August	[52]	65555
Côte d'Ivoire	Bouaké, Dar-és-Salam	-5.03	7.68[i]	1	1992	12	1992	1.10[ẽ]	4727[gc]	52.00	-8.0	-8.0	-8.0	8	4	05	04	13	-8	-8	I	no transmission in August and November	[52]	65555
Côte d'Ivoire	Bouaké, Kennedy	-5.03	7.68[i]	1	1991	12	1991	2.00[ẽ]	6700[gc]	134.00	-8.0	-8.0	-8.0	8	4	07	03	11	-8	-8	R	-	[52]	65555
Côte d'Ivoire	Bouaké, Kennedy	-5.03	7.68[i]	1	1992	12	1992	1.80[ẽ]	5878[gc]	105.80	-8.0	-8.0	-8.0	7	5	09	04	11	-8	-8	R	-	[52]	65555
Côte d'Ivoire	Bouaké, market garden districts	-5.03	7.68[i]	1	1992	12	1992	2.70[ẽ]	4380[g]	88.00	-8.0	-8.0	-8.0	8	-8	-8	04	11	R	-8	R	-	[53]	65555
Côte d'Ivoire	Bouaké, rice field district	-5.03	7.68[i]	1	1992	12	1992	0.90[ẽ]	19467[g]	126.00	-8.0	-8.0	-8.0	9	-8	-8	04	12	I	-8	I	-	[53]	65555
Côte d'Ivoire	Bouaké, Sokoura	-5.03	7.68[i]	1	1991	12	1991	1.80[ẽ]	3739[gc]	67.30	-8.0	-8.0	-8.0	8	3	05	03	10	-8	-8	R	-	[52]	65555
Côte d'Ivoire	Bouaké, Sokoura	-5.03	7.68[i]	1	1992	12	1992	2.40[ẽ]	3708[gc]	89.00	-8.0	-8.0	-8.0	8	5	09	04	11	-8	-8	R	-	[52]	65555
Côte d'Ivoire	Bouaké, Tolakouadiokro	-5.03	7.68[i]	1	1991	12	1991	0.80[ẽ]	13000[gc]	104.00	-8.0	-8.0	-8.0	11	5	08	03	13	-8	-8	I	no transmission in December	[52]	65555
Côte d'Ivoire	Bouaké, Tolakouadiokro	-5.03	7.68[i]	1	1992	12	1992	0.70[ẽ]	22143[gc]	155.00	-8.0	-8.0	-8.0	-8	8	08	02	-8	-8	-8	I	-	[52]	65555
Côte d'Ivoire	Bouaké, Zone	-5.03	7.68[i]	1	1991	12	1991	1.10[ẽ]	9364[gc]	103.00	-8.0	-8.0	-8.0	11	5	10	03	13	-8	-8	I	no transmission in December	[52]	65555
Côte d'Ivoire	Bouaké, Zone	-5.03	7.68[i]	1	1992	12	1992	0.90[ẽ]	7000[gc]	63.00	-8.0	-8.0	-8.0	8	5	08	04	11	-8	-8	I	-	[52]	65555
Côte d'Ivoire	Katiola district, 8 villages (no rice cultivation)	-999.00	-99.00	3	1997	1	1998	-8.00	-8	-8.00	87.0	83.0	90.0	8	5	05	04	11	-8	-8	R	*PR*: ⋆(0-9); villages are Angolokaha, Doussoulokaha, Folofonkaha, Kabolo, Ounadiékaha, Petionara, Sérigobokaha, and Timorokaha	[54]	65555
Côte d'Ivoire	Korhogo district, 8 villages (rice cultivation during the dry season)	-999.00	-99.00	3	1997	1	1998	-8.00	-8	-8.00	79.0	75.0	81.0	7	-8	05	06	12	-8	-8	I	*PR*: ⋆(0-9); villages are Gbahaouakaha, Kohotieri, Koumbolikaha, Lamékaha, Nambékaha, Nombolo, Nongotchénékaha, and Zémongokaha	[54]	65536
Côte d'Ivoire	Korhogo district, 8 villages (rice cultivation during the rainy season)	-999.00	-99.00	3	1997	1	1998	-8.00	-8	-8.00	84.0	80.0	86.0	8	-8	08	04	11	-8	-8	R	*PR*: ⋆(0-9); villages are Binguebougou, Fapaha, Kombolokoura, Kaforo, Karakpo, Kassoumbarga, Katiorkpo, and Tioro	[54]	65536
Côte d'Ivoire	Tiémélékro	-4.17	6.50[r]	2	2002	8	2002	3.25[f]	7662[gc]	249.00	-8.0	-8.0	-8.0	-8	-8	07	04	-8	-8	-8	R	-	[55]	65562
Côte d'Ivoire	Tiémélékro	-4.17	6.50[r]	2	2003	8	2003	7.36[f]	6264[gc]	461.00	-8.0	-8.0	-8.0	-8	-8	08	04	-8	-8	-8	R	-	[55]	65562
Côte d'Ivoire	Zatta	-5.39	6.88[r]	2	2002	8	2002	4.58[f]	17227[gc]	789.00	-8.0	-8.0	-8.0	12	-8	02	-1	-1	-8	-8	I	-	[55]	65562

to be continued

Table 1 – continued

country	place	long [°E]	lat [°N]	M1	YYY1	M2	YYY2	$CSPR_a$	HBR_a	EIR_a	PR_a	$PR_{min,a}$	$PR_{max,a}$	*Seas*	*MSeas*	*XSeas*	*SSeas*	*ESeas*	U_b [1]	U_a [2]	U_p	notes	ref	WMO#
Côte d'Ivoire	Zatta	-5.39	6.88[r]	2	2003	8	2003	1.39[f]	3453[gc]	48.00	-8.0	-8.0	-8.0	-8	-8	07	06	-8	-8	-8	R	interruption of rice irrigation in 2003	[55]	65562
Dominican Republic	Calle Duarte, Colonia Japonesa, La Bomba	-999.00	-99.00	7	1987	10	1988	0.03	5731	1.72	-8.0	-8.0	-8.0	-8	-8	-8	-8	-8	-8	-8	-8	-	[56]	-9999
Gambia, The	5 villages around Farafenni	-16.57	13.47[r]	4	2003	5	2003	-8.00[f]	-8	-8.00	-8.0	19.9	-8.0	-8	-8	-8	-8	-8	-8	-8	R	950 subjects from 5-45	[57]	61701
Gambia, The	Bakau	-16.68	13.48[b]	6	1988	5	1989	0.98[f]	104[ghc]	1.02[k]	2.0	-8.0	-8.0	-8	-8	-8	-8	-8	U	U	U	*PR*: ⋆(0-9)	[58]	61701
Gambia, The	Barokanda	-15.32	13.65[b]	8	1988	11	1988	2.97[fc]	3701[ĩ]	110.00	-8.0	-8.0	-8.0	5[d]	-8	09[d]	08[d]	12[d]	R	R2	R	reduced *EIR* after impregnated bed net usage	[59, 60]	61701
Gambia, The	Biran Giddo Ya	-999.00	-99.00	6	1986	12	1986	4.10[ẽ]	412[ĩ]	16.87[c]	-8.0	-8.0	-8.0	4	2	09	08	11	-8	-8	R	-	[61]	61701
Gambia, The	Bwiam	-16.09	13.23[b]	7	1991	1	1992	2.47[f]	37[hj]	0.92	39.5	-8.0	-8.0	-8	-8	-8	-8	-8	R	R2	R	*PR*: ⋆(1-4)	[62, 63]	61701
Gambia, The	Dasilami	-15.23	13.48[b]	8	1988	11	1988	1.29[fc]	1160[ĩ]	15.00	-8.0	-8.0	-8.0	5[d]	-8	09[d]	08[d]	12[d]	R	R2	R	reduced *EIR* after impregnated bed net usage	[59, 60]	61701
Gambia, The	Dasilami	-14.27	13.41[b]	7	1991	1	1992	2.23[f]	54[hj]	1.21	57.3[d]	-8.0	-8.0	-8	-8	-8	-8	-8	R	R2	R	*PR*: ⋆(1-4)	[62]	61687
Gambia, The	Dongoro Ba	-15.28	13.38[b]	8	1988	11	1988	17.86[fc]	448[ĩ]	80.00	-8.0	-8.0	-8.0	5[d]	-8	09[d]	08[d]	12[d]	R	R2	R	reduced *EIR* after impregnated bed net usage	[59, 60]	61701
Gambia, The	4 villages west of Farafenni	-999.00	-99.00	5	2001	6	2001	-8.00	-8	-8.00	-8.0	54.3	-8.0	3	-8	-8	09	11	-8	-8	R	*PR*: ⋆(0.5-15)	[64]	61701
Gambia, The	Jahally	-14.97	13.55[b]	7	1991	1	1992	0.95[f]	443[hj]	4.17	34.2[d]	-8.0	-8.0	-8	-8	-8	-8	-8	R	R2	R	*PR*: ⋆(1-4)	[62, 63]	-9999
Gambia, The	Jalangbereh	-15.40	13.38[b]	8	1988	11	1988	9.10[fc]	769[ĩ]	70.00	-8.0	-8.0	-8.0	5[d]	-8	09[d]	08[d]	12[d]	R	R2	R	reduced *EIR* after impregnated bed net usage	[59, 60]	61701
Gambia, The	Jessadi	-15.30	13.63[b]	8	1988	11	1988	2.68[fc]	2380[ĩ]	64.00	-8.0	-8.0	-8.0	5	-8	09	08	12	R	R2	R	reduced *EIR* after impregnated bed net usage	[59, 60]	61701
Gambia, The	Katamina	-15.28	13.55[b]	8	1988	11	1988	0.37[fc]	1076[ĩ]	4.00	-8.0	-8.0	-8.0	5[d]	-8	09[d]	08[d]	12[d]	R	R2	R	reduced *EIR* after impregnated bed net usage	[59, 60]	61701
Gambia, The	Kerewan	-16.09	13.49[b]	6	1992	11	1992	0.36	217[c]	0.78	-8.0	-8.0	-8.0	-8	-8	-8	-8	-8	R	R1	R	-	[63]	61701
Gambia, The	Kerewan	-16.09	13.49[b]	7	1991	1	1992	0.23[f]	211[hj]	0.44	28.7[d]	-8.0	-8.0	-8	-8	-8	-8	-8	R	R1	R	*PR*: ⋆(1-4)	[62, 63]	61701
Gambia, The	Kulari	-14.08	13.40[b]	7	1991	1	1992	7.65[f]	102[hj]	7.75	71.2	-8.0	-8.0	-8	-8	-8	-8	-8	R	R1	R	*PR*: ⋆(1-4)	[62, 63]	61687
Gambia, The	Madina	-15.25	13.52[b]	8	1988	11	1988	7.45[fc]	2376[ĩ]	177.00	-8.0	-8.0	-8.0	5[d]	-8	09[d]	08[d]	12[d]	R	R2	R	reduced *EIR* after impregnated bed net usage	[59, 60]	61701
Gambia, The	Male Kunda	-15.30	13.55[b]	8	1988	11	1988	2.60[fc]	2692[ĩ]	70.00	-8.0	-8.0	-8.0	5[d]	-8	09[d]	08[d]	12[d]	R	R2	R	reduced *EIR* after impregnated bed net usage	[59, 60]	61701
Gambia, The	Niawodurulung	-15.22	13.46[b]	8	1988	11	1988	0.00[fc]	1481[ĩ]	0.00	-8.0	-8.0	-8.0	5[d]	-8	09[d]	08[d]	12[d]	R	R2	R	reduced *EIR* after impregnated bed net usage	[59, 60]	61701
Gambia, The	Nyrimba Koyo Ya	-999.00	-99.00	6	1986	12	1986	1.61[ẽ]	155[ĩ]	2.49[c]	-8.0	-8.0	-8.0	4	2	09[d]	08	11	-8	-8	R	untreated bed net usage	[61]	-9999
Gambia, The	Pakali Ba	-15.25	13.50[b]	8	1988	11	1988	3.04[fc]	3253[ĩ]	99.00	-8.0	-8.0	-8.0	5[d]	-8	09[d]	08[d]	12[d]	R	R2	R	reduced *EIR* after impregnated bed net usage	[59, 60]	61701
Gambia, The	Salikene	-15.97	13.48[b]	7	1991	1	1992	0.55[f]	360[hj]	1.94	28.7	-8.0	-8.0	-8	-8	-8	-8	-8	R	R2	R	*PR*: ⋆(1-4)	[62, 63]	61701
Gambia, The	Sare Alpha	-13.98	13.37[b]	6	1992	11	1992	4.25[f]	-8[hj]	-8.00	-8.0	-8.0	-8.0	-8	-8	-8	-8	-8	R	R1	R	-	[63]	61687
Gambia, The	Sare Alpha	-13.98	13.37[b]	7	1991	1	1992	6.07[f]	187[hj]	11.15	71.2[d]	-8.0	-8.0	-8	-8	-8	-8	-8	R	R1	R	*PR*: ⋆(1-4)	[62, 63]	61687
Gambia, The	Saruja	-14.90	13.55[b]	6	1992	11	1992	0.73[f]	38[hjc]	2.79	-8.0	-8.0	-8.0	-8	-8	-8	-8	-8	I	R2	R	-	[63]	-9999
Gambia, The	Saruja	-14.90	13.55[b]	7	1991	1	1992	2.17[f]	231[hj]	5.00	34.2	-8.0	-8.0	-8	-8	-8	-8	-8	I	R2	R	*PR*: ⋆(1-4)	[62, 63]	-9999
Gambia, The	Sibanor	-16.20	13.21[b]	7	1991	1	1992	2.88[f]	113[hj]	3.24	39.5[d]	-8.0	-8.0	-8	-8	-8	-8	-8	R	R2	R	*PR*: ⋆(1-4)	[62, 63]	61701
Gambia, The	Sitahuma	-15.40	13.43[b]	8	1988	11	1988	2.23[fc]	3366[ĩ]	75.00	-8.0	-8.0	-8.0	5[d]	-8	09[d]	08[d]	12[d]	R	R2	R	reduced *EIR* after impregnated bed net usage	[59, 60]	61701
Gambia, The	Sutukoba	-14.02	13.50[b]	7	1991	1	1992	2.94[f]	34[hj]	0.99	57.3	-8.0	-8.0	-8	-8	-8	-8	-8	R	R2	R	*PR*: ⋆(1-4)	[62, 63]	61687
Gambia, The	Tally Ya	-15.72	13.58[i]	6	1986	12	1986	2.31[ẽ]	682[ĩ]	15.75[c]	-8.0	-8.0	-8.0	5	3	09[d]	07	11	-8	-8	R	untreated bed net usage	[61]	-9999
Gambia, The	Turan	-15.72	13.58[i]	6	1986	12	1986	2.70[ẽ]	890[ĩ]	24.04[c]	-8.0	-8.0	-8.0	5	3	09	07	11	-8	-8	R	-	[61]	61701

to be continued

Table 1 – continued

country	place	long [°E]	lat [°N]	M1	YYY1	M2	YYY2	$CSPR_a$	HBR_a	EIR_a	PR_a	$PR_{min,a}$	$PR_{max,a}$	*Seas*	*MSeas*	*XSeas*	*SSeas*	*ESeas*	U_b [1]	U_a [2]	U_p	notes	ref	WMO#
Gambia, The	Wellingara Ba	-15.26	13.41[b]	8	1988	11	1988	2.19[fc]	1553[i]	34.00	-8.0	-8.0	-8.0	5[d]	-8	09[d]	08[d]	12[d]	R	R2	R	reduced *EIR* after impregnated bed net usage	[59,60]	61701
Ghana	Kassena Nankana district	-1.44	10.76[r]	5	2001	11	2001	-8.00	-8	-8.00	59.2[c]	43.6	76.4	-8	-8	-8	-8	-8	-8	R2	I	*PR*: $\star$(0.5-15)	[65]	65518
Ghana	Kassena Nankana district (irrigated)	-1.44	10.76[r]	6	2001	5	2002	4.70[f]	13404[gc]	630.00	-8.0	-8.0	-8.0	8	3	09	06	13	-8	R2	I	-	[66]	65518
Ghana	Kassena Nankana district (lowland)	-1.44	10.76[r]	6	2001	5	2002	19.00[f]	1895[gc]	360.00	-8.0	-8.0	-8.0	5	2	09	07	11	-8	R2	R	-	[66]	65518
Ghana	Kassena Nankana district (morbidity study)	-1.44	10.76[r]	10	1990	9	1991	-8.00	-8	-8.00	74.2	53.3	84.5	-8	-8	-8	-8	-8	-8	R2	I	*PR*: $\star$(0 to 7)	[67]	65518
Ghana	Kassena Nankana district (mortality study)	-1.44	10.76[r]	10	1990	9	1991	-8.00	-8	-8.00	87.1	76.5	94.2	-8	-8	-8	-8	-8	-8	R2	I	*PR*: $\star$(0 to 7)	[67]	65518
Ghana	Kassena Nankana district (rocky highland)	-1.44	10.76[r]	6	2001	5	2002	10.60[f]	2151[gc]	228.00	-8.0	-8.0	-8.0	4	3	08	07	10	-8	R2	R	-	[66]	65518
Kenya	Mumias	0.18	34.49[b]	5	1995	3	1996	6.79[f]	700[g]	47.50	52.3	43.8	60.4	11	7	-8	-8	-8	R	PU	R	*PR*: $\star$	[68]	-9999
Liberia	Yekepa	-8.53	7.58[a]	11	-8	11	-8	-8.00	-8	-8.00	-8.0	-8.0	13.0	-8	-8	-8	-8	-8	-8	R2	R	*PR*: $\star$(2-9)	[69]	61849
Liberia	Yekepa, close (<3 km)	-8.55	7.56[a]	11	-8	11	-8	-8.00	-8	-8.00	-8.0	-8.0	22.0	-8	-8	-8	-8	-8	-8	R2	R	*PR*: $\star$(2-9); vector control & drug usage	[69]	61849
Liberia	Yekepa, middle (5-15 km)	-8.63	7.58[a]	11	-8	11	-8	-8.00	-8	-8.00	-8.0	-8.0	68.0	-8	-8	-8	-8	-8	-8	R2	R	*PR*: $\star$(2-9); vector control & sporadic treatment	[69]	61849
Liberia	Yekepa, far (>15 km)	-8.54	7.58[i]	11	-8	11	-8	-8.00	-8	-8.00	-8.0	-8.0	92.0	-8	-8	-8	-8	-8	-8	R2	R	*PR*: $\star$(2-9); vector control & drug usage	[69]	61849
Mali	Bamako, Sotuba suburb	-7.93	12.65[a]	6	1998	12	1998	-8.00[f]	-8[g]	3.49	-8.0	-8.0	-8.0	7	3	10	06	12	-8	R2	R	-	[70]	61291
Mali	Dokobougou	-6.13	14.17[i]	8	1995	3	1998	0.59[f]	-8[gh]	-8.00	48.0	18.0	77.0	-8	-8	-8	-8	-8	-8	-8	R	*PR*: $\star$(1-4), partially graphically derived	[71,72]	61265
Mali	Kalanampala	-6.87	14.15[m]	8	1995	3	1998	1.49[f]	-8[gh]	-8.00	46.0	32.0	57.0	-8	-8	-8	-8	-8	-8	-8	R	*PR*: $\star$(1-4), partially graphically derived	[71,72]	61265
Mali	Niessoumana	-5.97	14.31[m]	8	1995	3	1998	0.28[f]	-8[gh]	-8.00	51.1	24.0	78.0	-8	-8	-8	-8	-8	-8	-8	I	*PR*: $\star$(1-4), partially graphically derived	[71,72]	61265
Mali	Ténégué	-5.95	14.33[m]	8	1995	3	1998	0.15[f]	-8[gh]	-8.00	32.7	18.0	49.0	-8	-8	-8	-8	-8	-8	-8	I	*PR*: $\star$(1-4), partially graphically derived	[71,72]	61265
Mali	Tissana	-5.92	14.35[m]	8	1995	3	1998	0.29[f]	-8[gh]	-8.00	36.7	12.0	52.0	-8	-8	-8	-8	-8	-8	-8	I	*PR*: $\star$(1-4), partially graphically derived	[71,72]	61265
Mali	Toumakoro	-6.18	14.07[i]	8	1995	3	1998	0.69[f]	-8[gh]	-8.00	52.3	31.0	71.0	-8	-8	-8	-8	-8	-8	-8	R	*PR*: $\star$(1-4), partially graphically derived	[71,72]	61265
Nigeria	Garki district, 16 villages	-999.00	-99.00	2	1971	5	1972	-8.00	-8	-8.00	49.0	59.0	42.0	-8	-8	-8	-8	-8	-8	-8	R	graphically derived; ♦	[73]	-9999
Nigeria	Lagos, Lemu suburb	3.37	6.47[a]	1	2000	12	2000	2.54[f]	1891[ghc]	48.00	-8.0	-8.0	-8.0	12	-8	-8	-1	-1	-8	U	U	*Anopheles melas* and *Anopheles moucheti* maintain transmission in dry season	[74]	65344
Sao and Tomé and Príncipe	Príncipe	7.42	1.53[r]	5	1999	8	1999	-8.00	-8	-8.00	19.8	-8.0	-8.0	-8	-8	-8	-8	-8	-8	-8	R	*PR*: ♦	[75]	61934
Senegal	Aéré Lao	-14.30	16.40[b]	5	1982	8	1983	0.24[ẽ]	1600[g]	6.40	-8.0	12.0[d]	17.0[d]	4	2	11	09	12	R	R2	R	*PR*: unknown age classes; presence of Senegal river	[76]	61612
Senegal	Affiniam	-16.37	12.65[i]	1	1985	11	1985	0.03[ẽ]	66667[gc]	20.00	-8.0	-8.0	-8.0	5	3	09	07	11	R	R2	I	-	[77]	61695
Senegal	Affiniam	-16.37	12.65[i]	1	1986	11	1986	0.30[ẽ]	13000[gc]	39.00	-8.0	-8.0	-8.0	4	3	09	08[d]	11[d]	R	R2	I	-	[77]	61695
Senegal	Barkedji	-14.88	15.28[r]	6	1994	12	1994	2.19[f]	5870[ghc]	128.55	-8.0	-8.0	-8.0	4	2	10	09	12	R	R2	R	clay hollows → high HBR_a & EIR_a	[78]	61627
Senegal	Barkedji	-14.88	15.28[r]	7	1995	3	1996	1.52[f]	6684[ghc]	101.60	-8.0	-8.0	-8.0	6	2	09	08	14	R	R2	R	clay hollows → high HBR_a & EIR_a	[78]	61627
Senegal	Boké Diallobé	-14.00	16.10[b]	5	1982	8	1983	1.20[ẽ]	200[g]	0.80	-8.0	12.0	17.0	3	2	09	09	11	R	R2	R	*PR*: unknown age classes	[76]	61612
Senegal	Boundoum	-16.47	16.38[m]	7	1994	11	1994	-8.00	-8	-8.00	0.0	0.0	0.0	0	0	-5	-5	-5	-8	-8	I	*PR*: $\star$(0-9)	[79]	61489
Senegal	Dakar, district Centre	-17.44	14.70[a]	3	1996	2	1997	0.00[ẽ]	110[h]	0.00	1.4	0.4	1.9	0	0	-5	-5	-5	-8	U	U	*PR*: PR_a: $\star$(0-14) ♦	[80]	61641
Senegal	Dakar, Grande Niaye Mrash	-17.42	14.75[r]	5	1987	9	1988	0.55[ẽ]	22[ghc]	0.12	3.7	3.4	4.0	-8	-8	-8	-8	-8	-8	U	U	*PR*: ♦	[81]	61641
Senegal	Dakar, Grande Niaye Mrash	-17.42	14.75[r]	6	1987	6	1988	-8.00	-8	-8.00	5.6	3.6	7.5	-8	-8	-8	-8	-8	-8	U	U	*PR*: $\star$	[82]	61641
Senegal	Diagobel	-16.33	12.70[i]	1	1985	11	1985	0.00[ẽ]	-8[g]	0.00	-8.0	-8.0	-8.0	0	0	-5	-5	-5	R	R2	I	-	[77]	61695
Senegal	Diagobel	-16.33	12.70[i]	1	1986	11	1986	0.30[ẽ]	41000[gc]	123.00	-8.0	-8.0	-8.0	4	3	09[d]	08[d]	11[d]	R	R2	I	-	[77]	61695
Senegal	Diakhanor	-16.77	13.98[i]	6	1995	12	1997	-8.00	-8	-8.00	8.5[d]	1.9[d]	15.3[d]	2	-8	09	08	09	-8	-8	R	*PR*: unknown age classes	[83]	61679
Senegal	Diakhanor	-16.77	13.98[i]	6	1996	11	1996	-8.00	-8	-8.00	6.5	0.0	14.4	-8	-8	-8	-8	-8	-8	-8	R	*PR*: $\star$(0-9)	[84]	61679
Senegal	Diamballo	-16.95	15.02[i]	-8	1967	-8	1968	-8.00	-8	-8.00	-8.0	32.0[d]	50.0[d]	8[d]	-8	-8	07[d]	16[d]	R	-8	R	-	[85]	61641
Senegal	Dielmo	-16.42	13.72[b]	1	1990	12	1990	-8.00	-8	-8.00	85.0	-8.0	-8.0	-8	-8	-8	-8	-8	R	R2	S	*PR*: $\star$(2-9)	[86]	61679
Senegal	Dielmo	-16.42	13.72[b]	4	1990	3	1991	1.24[f]	9533[gc]	118.00	-8.0	-8.0	-8.0	12	5	07	-1	-1	R	R2	S	-	[87]	61679
Senegal	Dielmo	-16.42	13.72[b]	4	1991	3	1992	1.59[f]	18462[gc]	294.00	-8.0	-8.0	-8.0	12	5	07	-1	-1	R	R2	S	-	[87]	61679

to be continued

Table 1 – continued

country	place	long [°E]	lat [°N]	M1	YYY1	M2	YYY2	$CSPR_a$	HBR_a	EIR_a	PR_a	$PR_{min,a}$	$PR_{max,a}$	*Seas*	*MSeas*	*XSeas*	*SSeas*	*ESeas*	U_b [1]	U_a [2]	U_p	notes	ref	WMO#
Senegal	Dielmo	-16.42	13.72[b]	4	1992	3	1993	2.77[f]	8581[ghc]	237.80	-8.0	-8.0	-8.0	12	6	07	-1	-1	R	R2	S	-	[88]	61679
Senegal	Dielmo	-16.42	13.72[b]	4	1993	3	1994	0.91[f]	9795[ghc]	88.70	-8.0	-8.0	-8.0	11	4	10	-1	-1	R	R2	S	-	[88]	61679
Senegal	Dielmo	-16.42	13.72[b]	4	1994	3	1995	0.87[f]	17322[ghc]	150.10	-8.0	-8.0	-8.0	10	4	14	06	-8	R	R2	S	-	[88]	61679
Senegal	Dielmo	-16.42	13.72[b]	6	1990	5	1991	1.04[ẽ]	12500[gc]	130.00	89.3	-8.0	-8.0	12	4	07	-1	-1	R	R2	S	*PR*: $\star$(0-14)	[89]	61679
Senegal	Dielmo	-16.42	13.72[b]	6	1991	5	1992	1.57[ẽ]	22299[gc]	350.10	-8.0	-8.0	-8.0	12	6	09	-1	-1	R	R2	S	-	[89]	61679
Senegal	Diohine	-16.52	14.50[b]	1	1995	12	1995	1.95[f]	680[gh]	13.26[c]	56.7[d]	41.0[d]	82.0[d]	5	-8	09	07	11	R	R1	R	*PR*: $\star$(0-9)	[90]	61666
Senegal	Diomandou Dieri	-14.65	16.52[r]	6	1990	11	1991	0.00[ẽ]	3139[g]	0.00	-8.0	-8.0	-8.0	0	0	-5	-5	-5	-8	-8	I	-	[91]	61612
Senegal	Diomandou Toulde Galle	-14.71	16.56[m]	6	1990	11	1991	0.00[ẽ]	438[g]	0.00	-8.0	-8.0	-8.0	0	0	-5	-5	-5	-8	-8	R	-	[91]	61612
Senegal	Diomandou Walo	-14.62	16.56[m]	6	1990	11	1991	0.05[ẽ]	7483[g]	1.00	-8.0	-8.0	-8.0	1	1	08	08	08	-8	-8	I	-	[91]	61612
Senegal	Djifère	-16.77	13.93[i]	6	1995	12	1997	2.08[f]	789[ghc]	16.40	8.5	1.9	15.3	5	-8	11	11	15	-8	-8	S	*PR*: unknown age classes	[83]	61679
Senegal	Djifère	-16.77	13.93[i]	6	1996	11	1996	-8.00	-8	-8.00	4.9	0.0	14.4	-8	-8	-8	-8	-8	-8	-8	S	*PR*: $\star$(0-9)	[84]	61679
Senegal	Djilor	-16.33	14.07[i]	6	1995	12	1997	0.39[f]	2615[ghc]	10.20	12.9	1.1	31.4	6	-8	07	07	12	-8	-8	R	*PR*: unknown age classes	[83]	61679
Senegal	Kassack-Nord	-16.03	16.40[b]	9	1992	11	1994	0.00[f]	-8[g]	0.00	0.2	0.0	0.4	0	0	-5	-5	-5	R	R2	I	*PR*: $\star$(0-9)	[79]	61489
Senegal	Kotiokh	-16.58	14.48[b]	1	1995	12	1995	1.75[f]	1558[gh]	26.50	56.7	41.0	82.0	8	-8	09	06	13	R	R1	R	*PR*: $\star$(0-9); near permanent breeding	[90]	61666
Senegal	Maka-Diama	-16.40	16.20[b]	9	1992	11	1994	0.00[f]	-8[g]	0.00	0.5	0.0	1.0	0	0	-5	-5	-5	R	R2	S	*PR*: $\star$(0-9)	[79]	61600
Senegal	Ndiop	-16.42	13.75[b]	1	1993	12	1993	4.47[f]	1411[gc]	63.00	-8.0	-8.0	-8.0	4	2	09	07	10	R	R2	R	-	[92]	61679
Senegal	Ndiop	-16.42	13.75[b]	1	1993	12	1993	-8.00	-8	-8.00	18.0	-8.0	-8.0	-8	-8	-8	-8	-8	R	R2	R	*PR*: $\star$(2-9)	[86]	61679
Senegal	Ndiop	-16.42	13.75[b]	1	1994	12	1994	3.61[f]	471[gc]	17.00	-8.0	-8.0	-8.0	3	2	09	08	10	R	R2	R	-	[92]	61679
Senegal	Ndiop	-16.42	13.75[b]	1	1995	12	1995	4.05[f]	914[gc]	37.00	-8.0	-8.0	-8.0	3	2	09	08	10	R	R2	R	-	[92]	61679
Senegal	Ndiop	-16.42	13.75[b]	1	1996	12	1996	4.71[f]	149[gc]	7.00	-8.0	-8.0	-8.0	1	1	09	09	09	R	R2	R	-	[92]	61679
Senegal	Ngadiaga	-16.95	15.02[m]	3	1991	11	1991	-8.00	-8	-8.00	6.2	2.0	10.6	-8	-8	-8	-8	-8	R	-8	R	*PR*: $\star$(0-10)	[85]	61641
Senegal	Ngadiaga	-16.95	15.02[m]	8	1993	8	1993	-8.00	-8	-8.00	-8.0	0.0	-8.0	-8	-8	-8	-8	-8	R	-8	R	*PR*: $\star$(0-10)	[85]	61641
Senegal	Ngayokhème	-16.43	14.53[b]	1	1995	12	1995	1.80[f]	512[gh]	8.96[c]	56.7[d]	41.0[d]	82.0[d]	5	-8	09	07	11	R	R1	R	*PR*: $\star$(0-9)	[90]	61666
Senegal	Niakhar	-16.40	14.47[r]	2	1995	11	1995	-8.00	-8	-8.00	57.0[d]	41.0[d]	83.0[d]	-8	-8	-8	-8	-8	-8	-8	R	*PR*: $\star$(0-9)	[93]	61666
Senegal	Ousseuk	-16.18	12.84[m]	1	1985	11	1985	0.36[ẽ]	3611[gc]	13.00	-8.0	-8.0	-8.0	4	3	09[d]	07[d]	10[d]	-8	R2	I	-	[77]	61695
Senegal	Ousseuk	-16.18	12.84[m]	1	1986	11	1986	0.46[ẽ]	3487[gc]	16.00	-8.0	-8.0	-8.0	3	2	09[d]	08[d]	10[d]	-8	R2	I	-	[77]	61695
Senegal	Pikine	-17.40	14.75[b]	11	1979	1	1981	-8.00	-8	-8.00	8.8[d]	2.2[d]	13.5[d]	-8	-8	-8	-8	-8	U	-8	U	-	[94]	61641
Senegal	Pikine	-17.40	14.75[b]	12	1979	12	1980	0.55[de]	9145[sgc]	50.30[sk]	-8.0	-8.0	-8.0	12	4	10	-1	-1	U	-8	U	-	[94]	61641
Senegal	Pikine	-17.40	14.75[b]	12	1979	12	1980	0.55[ẽ]	7818[gc]	43.00	8.8	2.2	13.5	-9	4	-9	-9	-9	U	-8	U	-	[95]	61641
Senegal	Simal	-16.65	14.15[i]	6	1995	12	1997	2.20[f]	596[ghc]	13.10	12.9[d]	1.1[d]	31.4[d]	7[d]	-8	10	06[d]	12[d]	-8	-8	R	*PR*: unknown age classes	[83]	61679
Senegal	Takème	-16.20	12.82[i]	1	1985	11	1985	0.36[ẽ]	3056[gc]	11.00	-8.0	-8.0	-8.0	4	3	09	07	10	-8	R2	I	-	[77]	61695
Senegal	Takème	-16.20	12.82[i]	1	1986	11	1986	0.46[ẽ]	4783[gc]	22.00	-8.0	-8.0	-8.0	3	2	09	08	10	-8	R2	I	-	[77]	61695
Senegal	Tendimane	-16.30	12.77[i]	1	1985	11	1985	0.00[ẽ]	-8[g]	0.00	-8.0	-8.0	-8.0	0	0	-5	-5	-5	R	R2	I	-	[77]	61695
Senegal	Tendimane	-16.30	12.77[i]	1	1986	11	1986	0.30[ẽ]	11667[gc]	35.00	-8.0	-8.0	-8.0	4	3	09	08	11	R	R2	I	-	[77]	61695
Senegal	Thiaye	-16.96	14.99[m]	1	1992	12	1992	0.00[f]	4540[gh]	0.00	-8.0	3.2	-8.0	0	0	10	-5	-5	R	-8	R	*PR*: $\star$(0-10); wet depressions $\rightarrow$ high HBR_a	[85]	61641
Senegal	Thiaye	-16.96	14.99[m]	3	1991	12	1991	0.48[f]	2290[gh]	11.00	-8.0	2.9	5.7	2	2	-5	09	10	R	-8	R	*PR*: $\star$(0-10); wet depressions $\rightarrow$ high HBR_a	[85]	61641
Senegal	Thiaye	-16.96	14.99[m]	8	1993	8	1993	-8.00	-8	-8.00	-8.0	0.0	-8.0	-8	-8	-8	-8	-8	R	-8	R	*PR*: $\star$(0-10)	[85]	61641
Senegal	Thiaye	-17.07	14.92[r]	9	1992	10	1993	0.00[f]	1241[gh]	0.00	-8.0	-8.0	-8.0	0	0	-5	-5	-5	-8	-8	R	*PR*: $\star$(0-10); wet depressions $\rightarrow$ high HBR_a	[85,96]	61641
Senegal	Thies	-16.93	14.80[i]	-8	-8	-8	-8	-8.00	-8	-8.00	17.5	-8.0	-8.0	-8	-8	-8	-8	-8	-8	-8	R	*PR*: $\star$(0-14)	[97]	61641
Senegal	Wassdou	-13.33	13.35[r]	9	1992	11	1993	2.75[f]	7884[gh]	220.00	-8.0	-8.0	-8.0	7	3	09	07	13	-8	-8	R	-	[96,98]	61687
Sierra Leone	8 villages near Bo	-999.00	-99.00	3	1990	12	1990	-8.00	-8	-8.00	61.0	-8.0	-8.0	-8	-8	-8	-8	-8	-8	-8	R	*PR*: $\star$(0-7); data exist for single villages	[99]	-9999
Sierra Leone	Mendewa	-11.48	8.16[b]	1	1990	4	1991	9.60[f]	228[hc]	21.90	-8.0	-8.0	-8.0	11[d]	4[d]	06[d]	01[d]	11[d]	R	R2	R	-	[100]	-9999
Sierra Leone	Nengbema	-11.68	8.13[b]	1	1990	4	1991	5.25[f]	410[hc]	21.54	-8.0	-8.0	-8.0	11	4	06	01	11	R	R2	R	-	[100]	-9999
Sierra Leone	Njala-Komboya	-11.54	8.20[b]	1	1990	4	1991	7.63[f]	349[hc]	26.64	-8.0	-8.0	-8.0	11[d]	4[d]	06[d]	01[d]	11[d]	R	R2	R	-	[100]	-9999
Sierra Leone	Nyandeyama	-11.66	8.12[b]	1	1990	4	1991	8.11[f]	450[hc]	36.50	-8.0	-8.0	-8.0	11[d]	4[d]	06[d]	01[d]	11[d]	R	R2	R	-	[100]	-9999
Sudan	Asar	13.75	35.25[a]	10	1998	8	1999	-8.00	-8	-8.00	26.5	1.8	53.2	-8	-8	-8	-8	-8	-8	R2	R	-	[101]	-9999
Uganda	Kampala Mulago III parish	32.58	0.32[i]	11	2004	4	2005	-8.00	-8	-8.00	19.0	-8.0	-8.0	-8	-8	-8	-8	-8	-8	-8	U	*PR*: $\star$(1-10)	[102]	63608

References

1. Hay SI, Rogers DJ, Toomer JF, Snow RW: **Annual *Plasmodium falciparum* entomological inoculation rates (EIR) across Africa: literature survey, internet access and review.** *Trans R Soc Trop Med Hyg* 2000, **94**:113–127.
2. Hay SI, Guerra CA, Tatem AJ, Atkinson PM, Snow RW: **Urbanization, malaria transmission and disease burden in Africa.** *Nat Rev Microbiol* 2005, **3**:81–90.
3. Port GR, Boreham PFL, Bryan JH: **The relationship of host size to feeding by mosquitoes of the *Anopheles gambiae* Giles complex (Diptera: Culicidae).** *Bull Entomol Res* 1980, **70**:133–144.
4. Akogbéto M: **Le paludisme côtier lagunaire à Cotonou: données entomologiques.** *Cahiers Santé* 2000, **10**:267–27.
5. Akogbéto M, Chippaux JP, Coluzzi M: **Le paludisme urbain côtier à Cotonou (République du Bénin). Étude entomologique.** *Revue d'Epidémiologie et de Santé Publique* 1992, **40**:233–239.
6. Akogbéto M: **Étude entomologique sur la transmission du paludisme côtier lagunaire: cas d'un village construit sur un lac d'eau sâumatre.** *Ann Soc Belg Med Trop* 1995, **75**:219–227.
7. Akogbéto M, Nahum A: **Impact des moustiquaires imprégnées de deltaméthrine sur la transmission du paludisme dans un milieu côtier lagunaire, Bénin.** *Bull Soc Pathol Exot* 1996, **89**:291–298.
8. Carnevale P, Robert V: **Introduction of irrigation in Burkina Faso and its effect on malaria transmission.** In *Effects of Agricultural Development on Vector-borne Diseases*. Edited by FAO, Rome, Italy: FAO 1987:57–67.
9. Boudin C, Olivier M, Molez JF, Chiron JP, Ambroise-Thomas P: **High human malarial infectivity to laboratory-bred *Anopheles gambiae* in a village in Burkina Faso.** *Am J Trop Med Hyg* 1993, **48**:700–706.
10. Modiano D, Petrarca V, Sirima BS, Nebie I, Diallo D, Esposito F, Coluzzi M: **Different response to *Plasmodium falciparum* malaria in West African sympatric ethnic groups.** *Proc Natl Acad Sci U S A* 1996, **93**:13206–13211.
11. Gazin P, Robert V, Cot M, Simon J, Halna JM, Darriet F, Legrand D, Carnevale P, Ambroise-Thomas P: **Le paludisme dans l'Oudalan, région sahélienne du Burkina Faso.** *Ann Soc Belg Med Trop* 1988, **68**:255–264.
12. Lochouarn L, Gazin I: **La transmission du paludisme dans la ville de Bobo-Dioulasso (Burkina Faso).** *Ann Soc Belg Med Trop* 1993, **73**:287–296.
13. Robert V, Gazin I, Ouédraogo V, Carnevale I: **Le paludisme urbain à Bobo-Dioulasso (Burkina Faso). 1. Etude entomologique de la transmission.** *Cahiers O.R.S.T.O.M. Série Entomologie Médicale et Parasitologie* 1986, **24**:121–128.
14. Robert V, Gazin P, Benasseni R, Carnevale P: **Le paludisme urbain à Bobo-Dioulasso (Burkina Faso).** In *Urbanisation et santé dans le Tiers Monde: transition épidémiologique, changement social et soins de santé primaires*. Edited by Salem G, Emile J, Paris, France: ORSTROM 1989:181–185.
15. Gazin P, andand P Carnevale VR: **Le paludisme urbain à Bobo-Dioulasso (Burkina Faso). 2. Les indices paludologiques.** *Cahiers O.R.S.T.O.M. Série Entomologie Médicale et Parasitologie* 1987, **25**:27–31.
16. Gazin P, Goncalves K, Koné B, Lochouam L: **Incidence des accès palustres dans un quartier de la ville de Bobo-Dioulasso (Burkina Faso).** *Bull Soc Pathol Exot* 1996, **89**:200–203.
17. Robert V, Gazin P, Boudin C, Molez JF, Oueddraogo V, Carnevale P: **La transmission du paludisme en zone de savane arboreé et en zone rizicole des Bobo Dioulasso (Burkina Faso).** *Ann Soc Belg Med Trop* 1985, **65**:201–214.
18. Rossi P, Belli A, Mancini L, Sabatinelli G: **Enquête entomologique longitudinale sur la transmission du paludisme à Ouagadougou (Burkina Faso).** *Parassitologia* 1986, **28**:1–15.
19. Sabatinelli G, Bosman A, Lamizana L, Rossi P: **Prévalence du paludisme à Ouagadougou et dans le milieu rural limitrophé en periode de transmission maximale.** *Parassitologia* 1986, **28**:17–31.

20. Boudin C, Robert V, Verhave JP, Carnevale P, Ambroise-Thomas P: ***Plasmodium falciparum* and *P. malariae* epidemiology in a West African village.** *Bull World Health Org* 1991, **69**:199–205.
21. Boudin C, Robert V, Carnevale P, Ambroise-Thomas P: **Epidemiology of *Plasmodium falciparum* in a rice field and a savanna area in Burkina Faso. Comparative study on the acquired immunoprotection in native populations.** *Acta Trop* 1992, **51**:103–111.
22. Robert V, Carnevale P, Ouédraogo V, Petrarca V, Coluzzi M: **La transmission du paludisme humain dans un village de savane du sud-ouest du Burkina Faso.** *Ann Soc Belg Med Trop* 1988, **68**:107–121.
23. Boudin C, Robert V, Carnevale P, Ambroise TP: **Epidemiology of *Plasmodium falciparum* in a rice field and a savanna area in Burkina Faso: seasonal fluctuations of gametocytaemia and malaria infectivity.** *Ann Trop Med Parasitol* 1991, **85**:377–385.
24. Carnevale P, Robert V, Boudin C, Halna JM, Pazart L, Gazin P, Richard A, Mouchet J: **La lutte contre le paludisme par des moustiquaires imprégnées de pyréthrinoides au Burkina Faso.** *Bull Soc Pathol Exot* 1988, **81**:832–846.
25. Gazin P, Robert V, Cot M, Carnevale P: ***Plasmodium falciparum* incidence and patency in a high seasonal transmission area of Burkina Faso.** *Trans R Soc Trop Med Hyg* 1988, **82**:50–55.
26. Ouédraogo AL, de Vlas SJ, Nébié I, Ilboudo-Sanogo E, Bousema JT, Ouattara AS, Verhave JP, Cuzin-Ouattara N, Sauerwein RW: **Seasonal patterns of *Plasmodium falciparum* gametocyte prevalence and density in a rural population of Burkina Faso.** *Acta Trop* 2008, **105**:28–34.
27. Robert V, Carnevale P: **Influence of deltamethrin treatment of bed nets on malaria transmission in the Kou valley, Burkina Faso.** *Bull World Health Org* 1991, **69**:735–740.
28. Meunier JY, Safeukui I, Fontenille D, Boudin C: **Etude de la transmission du paludisme dans une future zone d'essai vaccinal en forêt équatoriale du sud Cameroun.** *Bull Soc Pathol Exot* 1999, **92**:309–312.
29. Bonnet S, Gouagna LC, Paul RE, Safeukui I, Meunier JY, Boudin C: **Estimation of malaria transmission from humans to mosquitoes in two neighbouring villages in south Cameroon: evaluation and comparison of several indices.** *Trans R Soc Trop Med Hyg* 2003, **97**:53–59.
30. Njan Nloga A, Robert V, Toto JC, Carnevale P: ***Anopheles moucheti*, vecteur principal du paludisme au sud Cameroun.** *Bulletin de Liaison et Documentation OCEAC* 1993, **26**:63–67.
31. Bonnet S, Paul RE, Gouagna C, Safeukui I, Meunier JY, Gounoue R, Boudin C: **Level and dynamics of malaria transmission and morbidity in an equatorial area of South Cameroon.** *Trop Med Int Health* 2002, **7**:249–256.
32. Robert V, Le Goff G, Toto JC, Mulder L, Fondjo E, Manga L, Carnevale P: **Anthropophilic mosquitoes and malaria transmission at Edea, Cameroon.** *Trop Med Parasitol* 1993, **44**:14–18.
33. Manga L, Fondjo E, Carnevale P, Robert V: **Importance of low dispersion of *Anopheles gambiae* (Diptera: Culicidae) on malaria transmission in hilly towns in South Cameroon.** *J Med Entomol* 1993, **30**:936–938.
34. Manga L, Toto JC, Le Goff G, Brunhes J: **The bionomics of *Anopheles funestus* and its role in malaria transmission in a forested area of southern Cameroon.** *Trans R Soc Trop Med Hyg* 1997, **91**:387–388.
35. Quakyi IA, Leke RGF, Befidi-Mengue R, Tsafack M, Bomba-Nkolo D, Manga L, Tchinda V, Njeungue E, Kouontchou S, Fogako J, Nyonglema P, Harun LT, Djokam R, Sama G, Eno A, Megnekou R, Metenou S, Ndountse L, Same-Ekobo A, Alake G, Meli J, Ngu J, Tietche F, Lohoue J, Mvondo JL, Wansi E, Leke R, Folefack A, Bigoga J, Bomba-Nkolo C, Titanji V, Walker-Abbey A, Hickey MA, Johnson AH, Taylor DW: **The epidemiology of *Plasmodium falciparum* malaria in two Cameroonian villages: Simbok and Etoa.** *Am J Trop Med Hyg* 2000, **63**:222–230.
36. Antonio-Nkondjio C, Simard F, Awono-Ambene P, Ngassam P, Toto JC, Tchuinkam T, Fontenille D: **Malaria vectors and urbanization in the equatorial forest region of south Cameroon.** *Trans R Soc Trop Med Hyg* 2005, **99**:347–354.

37. Le Goff G, Robert V, Fondjo E, Carnevale P: **Efficacy of insecticide impregnated bed-nets to control malaria in a rural forested area in southern Cameroon.** *Mem Inst Oswaldo Cruz* 1992, **87 (Suppl. 3)**:355–359.

38. Wanji S, Tanke T, Atanga SN, Ajonina C, Nicholas T, Fontenille D: ***Anopheles* species of the mount Cameroon region: biting habits, feeding behaviour and entomological inoculation rates.** *Trop Med Int Health* 2003, **8**:643–64.

39. Manga L, Bouchite B, Toto JC, Froment A: **La faune anophélienne et la transmission du paludisme dans une zone de transition forêt/savane au centre de Cameroun.** *Bull Soc Pathol Exot* 1997, **90**:128–130.

40. Manga L, Toto JC, Carnevale P: **Malaria vectors and transmission in an area deforested for a new international airport in southern Cameroon.** *Annales de la Société Belge de Medecine Tropicale* 1995, **75**:43–49.

41. Carnevale P, Le Goff G, Toto JC, Robert V: ***Anopheles nili* as the main vector of human malaria in villages of southern Cameroon.** *Med Vet Entomol* 1992, **6**:135–138.

42. Fontenille D, Meunier JY, Nkondjio CA, Tchuinkam T: **Use of circumsporozoite protein enzyme-linked immunosorbent assay compared with microscopic examination of salivary glands for calculation of malaria infectivity rates in mosquitoes (Diptera: Culicidae) from Cameroon.** *J Med Entomol* 2001, **38**:451–454.

43. Antonio-Nkondjio C, Awono-Ambene P, Toto JC, Meunier MY, Nyambam SZKR, Wondji CS, Tchuinkam T, Fontenille D: **High malaria transmission intensity in a village close to Yaounde, the capital city of Cameroon.** *J Med Entomol* 2002, **39**:350–355.

44. van der Kolk M, Tebo AE, Nimpaye H, Ndombol DN, Sauerwein RW, Eling WMC: **Transmission of *Plasmodium falciparum* in urban Yaoundé, Cameroon, is seasonal and age-dependent.** *Trans R Soc Trop Med Hyg* 2003, **97**:375–379.

45. Manga L, Traore O, Cot M, Mooh E, Carnevale P: **Le paludisme dans la ville de Yaoundé (Cameroon). 3. - Étude parasitologique dans deux quartiers centraux.** *Bull Soc Pathol Exot* 1993, **86**:56–61.

46. Tchuinkam T, Mulder B, Dechering K, Stoffels H, Verhave JP, Cot M, Carnevale P, Meuwissen JHET, Robert V: **Experimental infections of *Anopheles gambiae* with *Plasmodium falciparum* of naturally infected gametocyte carriers in Cameroon: factors influencing the infectivity to mosquitoes.** *Trop Med Parasitol* 1993, **44**:271–276.

47. Fondjo E, Robert V, Le Goff G, Toto JC, Carnevale P: **Le paludisme urbain à Yaoundé (Cameroun). 2. Etude entomologique dans deux quartiers peu urbanises.** *Bull Soc Pathol Exot* 1992, **85**:57–63.

48. Manga L, Robert V, Messi J, Desfontaine MA, Carnevale P: **Le paludisme urbain à Yaoundé (Cameroun). 1. É entomologique dans deux quartiers centraux.** *Mémoires de la Société Royale Belge d'Entomologie* 1992, **35**:155–162.

49. Trape JF, Zoulani A: **Études sur le paludisme dans une zone de mosaique forêt-savane d'Afrique centrale, la région de Brazzaville. I. Résultats des enquêtes entomologiques.** *Bull Soc Pathol Exot* 1987, **80**:84–99.

50. Trape JF: **Études sur le paludisme dans une zone de mosaique forêt-savane d'Afrique centrale, la région de Brazzaville. II. Densités parasitaires.** *Bull Soc Pathol Exot* 1987, **80**:520–531.

51. Dossou-Yovo J, Doannio JMC, Diarrassouba S, Chauvancy G: **Malaria in Côte d'Ivoire wet savannah region: the entomological input.** *Trop Med Parasitol* 1995, **46**:263–269.

52. Dossou-Yovo J, Doannio JMC, Diarrassouba S, Chauvancy G: **Impact d'aménagements de rizières sur la transmission du paludisme dans la ville de Bouaké, Côte d'Ivoire.** *Bulletin de la Société de pathologie exotique* 1998, **91**:327–333.

53. Dossou-Yovo J, Doannio J, Rivière F, Duval J: **Rice cultivation and malaria transmission in Bouaké city (Côte d'Ivoire).** *Acta Trop* 1994, **57**:91–94.

54. Henry MC, Rogier C, Nzeyimana I, Assi SB, Dossou-Yovo J, Audibert M, Mathonnat J, Keundjian A, Akodo E, Teuscher T, Carnevale P: **Inland valley rice production systems and malaria infection and disease in the savannah of Côte d'Ivoire.** *Trop Med Int Health* 2003, **8**:449–458.

55. Koudou BG, Tano Y, Doumbia M, Nsanzabana C, Cissé G, Girardin O, Dao D, N'Goran EK, Vounatsou P, Bordmann G, Keiser J, Tanner M, Utzinger J: **Malaria transmission dynamics in central Côte d'Ivoire: the influence of changing patterns of irrigated rice agriculture.** *Med Vet Entomol* 2005, **19**:27–37.
56. Mekuria YR, Granados R, Tidwell MA, Williams DC, Wirtz RA, Roberts DR: **Malaria transmission potential by *Anopheles* mosquitoes of Dajabon, Dominican Republic.** *J Am Mosq Control Assoc* 1991, **7**:456–461.
57. Nwakanma D, Kheir A, Sowa M, Dunyo S, Jawara M, Pinder M, Milligan P, Walliker D, Babiker HA: **High gametocyte complexity and mosquito infectivity of *Plasmodium falciparum* in The Gambia.** *Int J Parasitol* 2008, **38**:219–227.
58. Lindsay SW, Campbell H, Adiamah JH, Greenwood AM, Bangali JE, Greenwood BM: **Malaria in a peri-urban area of The Gambia.** *Ann Trop Med Parasitol* 1990, **84**:553–562.
59. Lindsay SW, Alonso PL, Schellenberg JRMA, Hemingway J, Thomas PJ, Shenton FC, Greenwood BM: **A malaria control trial using insecticide-treated bed nets and targeted chemoprophylaxis in a rural area of The Gambia, West Africa: 3. entomological characteristics of the study area.** *Trans R Soc Trop Med Hyg* 1993, **87 (Suppl. 2)**:19–23.
60. Lindsay SW, Alonso PL, Schellenberg JRMA, Hemingway J, Adiamah JH, Shenton FC, Jawara M, Greenwood BM: **A malaria control trial using insecticide-treated bed nets and targeted chemoprophylaxis in a rural area of The Gambia, West Africa: 7. impact of permethrin-impregnated bed nets on malaria vectors.** *Trans R Soc Trop Med Hyg* 1993, **87 (Suppl. 2)**:45–51.
61. Lindsay SW, Shenton FC, Snow RW, Greenwood BM: **Responses of *Anopheles gambiae* complex mosquitoes to the use of untreated bednets in The Gambia.** *Med Vet Entomol* 1989, **3**:253–262.
62. Thomson MC, D'Alessandro U, Bennett S, Connor SJ, Langerock P, Jawara M, Todd J, Greenwood BM: **Malaria prevalence is inversely related to vector density in The Gambia, West Africa.** *Trans R Soc Trop Med Hyg* 1994, **88**:638–643.
63. Thomson MC, Adiamah JH, Connor SJ, Jawara M, Bennett S, D'Alessandro U, ones MQ, Langerock P, Greenwood BM: **Entomological evaluation of The Gambia's national impregnated bednet programme.** *Ann Trop Med Parasitol* 1995, **89**:229–241.
64. Dunyo S, Milligan P, Edwards T, Sutherland C, Targett G, Pinder M: **Gametocytaemia after drug treatment of asymptomatic *Plasmodium falciparum*.** *PLoS Clin Trials* 2006, **1**:e20.
65. Koram KA, Owusu-Agyei S, Fryauff DJ, Anto F, Atuguba F, Hodgson A, Hoffmann SL, Nkrumah FK: **Seasonal profiles of malaria infection, anaemia, and bednet use among age groups and communities in northern Ghana.** *Trop Med Int Health* 2003, **8**:793–802.
66. Appawu M, Owusu-Agyei S, Dadzie S, Asoala V, Anto F, Koram K, Rogers W, Nkrumah F, Hoffman SL, Fryauff DJ: **Malaria transmission dynamics at a site in northern Ghana proposed for testing malaria vaccines.** *Tropical medicine and international health* 2004, **9**:164–170.
67. Binka FN, Morris SS, Ross DA, Arthur P, Aryeetey ME: **Patterns of malaria morbidity and mortality in children in northern Ghana.** *Trans R Soc Trop Med Hyg* 1994, **88**:381–385.
68. Shililu J, Maier WA, Seitz HM, Orago AS: **Seasonal density, sporozoite rates and entomological inoculation rates of *Anopheles gambiae* and *Anopheles funestus* in a high-altitude sugarcane growing zone in western Kenya.** *Trop Med Int Health* 1998, **3**:706–710.
69. Björkman A, Hedman P, Brohult J, Willcox M, Diamant I, Pehrsson PO, Rombo L, Bengtsson E: **Different malaria control activities in an area of Liberia - effects on malariometric parameters.** *Ann Trop Med Parasitol* 1985, **79**:239–246.
70. Sagara I, Sangaré D, Dolo G, Guindo A, Sissoko M, Sogoba M, Niambélé MB, Yalcoué D, Kaslow DC, Dicko A, Klion AD, Diallo D, Miller LH, Touré Y, Doumbo O: **A high malaria reinfection rate in children and young adults living under a low entomological inoculation rate in a periurban area of Bamako, Mali.** *Am J Trop Med Hyg* 2002, **66**:310–31.
71. Dolo G, Briët OJ, Dao A, Traoré SF, Bouaré M, Sogoba N, Niaré O, Bagayogo M, Sangaré D, Teuscher T, Touré YT: **Malaria transmission in relation to rice cultivation in the irrigated Sahel of Mali.** *Acta Trop* 2004, **89**:147–159.

72. Sissoko MS, Dicko A, Briët OJ, Sissoko M, Sagara I, Keita HD, Sogoba M, Rogier C, Touré YT, Doumbo OK: **Malaria incidence in relation to rice cultivation in the irrigated Sahel of Mali.** *Acta Trop* 2004, **89**:161–170.
73. Molineaux L, Storey J, Cohen JE, Thomas A: **A longitudinal study of human malaria in the West African savanna in the absence of control measures: relationship between different *Plasmodium* species, in particular *P,,falciparum* and *P. malariae*.** *Am J Trop Med Hyg* 1980, **29**:725–737.
74. Awolola TS, Okwa O, Hunt RH, Ogunrinade AF, Coetzee M: **Dynamics of the malaria-vector populations in coastal Lagos, south-western Nigeria.** *Ann Trop Med Parasitol* 2002, **96**:75–82.
75. Hagmann R, Charlwood JD, Gil V, Ferreira C, do Rosário V, Smith TA: **Malaria and its possible control on the island of Príncipe**. *Malar J* 2003, **2**:15.
76. Vercruysse J: **Étude entomologique sur la transmission du paludisme humain dans le bassin du fleuve Sénégal (Senegal).** *Ann Soc Belg Med Trop* 1985, **65 (Suppl. 2)**:171–179.
77. Faye O, Gaye O, Faye O, Diallo S: **La transmission du paludisme dans des villages éloignés ou situés en bordure de la mangrove au Sénégal.** *Bull Soc Pathol Exot* 1994, **87**:157–163.
78. Le Masson JJ, Fontenille D, Lochouam L, Dia I, Simard F, Ba K, Diop A, Diatta M, Molez JF: **Comparison of behavior and vector efficiency of *Anopheles gambiae* and *An. arabiensis* (Diptera: Culicidae) in Barkedji, a Sahelian area of Senegal.** *J Med Entomol* 1997, **34**:396–403.
79. Faye O, Fontenille D, Gaye O, Sy N, Molez JF, Konaté L, Hebrard G, Herve JP, Trouillet J, Diallo S, Mouchet J: **Paludisme et riziculture dans le delta du fleuve Sénégal (Sénégal).** *Ann Soc Belg Med Trop* 1995, **75**:179–189.
80. Diallo S, Konaté L, Ndir O, Dieng T, Dieng Y, Bah IB, Faye O, Gaye O: **Le paludisme dans le district sanitaire centre de Dakar (Sénégal). Données entomologiques, parasitologiques et cliniques.** *Cahiers Santé* 2000, **10**:221–229.
81. Trape JF, Lefebvre-Zante E, Legros F, Ndiaye G, Bouganali H, Druilhe P, Salem G: **Vector density gradients and the epidemiology of urban malaria in Dakar, Senegal.** *Am J Trop Med Hyg* 1992, **47**:181–189.
82. Trape JF, Lefebvre-Zante E, Legros F, Druilhe P, Rogier C, Bouganali H, Salem G: **Malaria morbidity among children exposed to low seasonal transmission in Dakar, Senegal and its implications for malaria control in tropical Africa.** *Am J Trop Med Hyg* 1993, **48**:748–756.
83. Diop A, Molez JF, Konate L, Fontenille D, Gaye O, Diouf M, Diagne M, Faye O: **Rôle d'*Anopheles melas* Theobald (1903) dans la transmission du paludisme dans la mangrove du Saloum au Sénégal.** *Parasite* 2002, **9**:239–246.
84. Diop A, Konaté L, Molez JF, Diouf M, Gaye O, Fontenille D, Diagne M, Faye O: **Le paludisme en zone de mangrove du delta du Saloum (Sénégal).** *Cahiers Santé* 2006, **16**:253–257.
85. Faye O, Gaye O, Fontenille D, Hebrard G, Konaté L, Sy N, Hervé JP, Touré Y, Diallo S, Molez JF, Mouchet J: **La sécheresse et la baisse du paludisme dans les Niayes du Sénégal.** *Cahiers Santé* 1995, **5**:299–305.
86. Rogier C, Trape JF: **Etude de l'aquisition de la prémunition en zone d'holo- et de méso-endémie palustre à Dielmo et à Nidiop (Senegal): résultats préliminaires, 1990-1994.** *Médecine Tropicale* 1995, **55**:71–76.
87. Konaté L, Diagne N, Brahimi K, Faye O, Legros F, Rogier C, Petrarca V, Trape JF: **Biologie des vecteurs et transmission de *Plasmodium falciparum, P. malariae* and *P. ovale* dans un village de savane d'Afrique de l'ouest (Dielmo, Sénégal)**. *Parasite* 1994, **1**:325–333.
88. Fontenille D, Lochouarn L, Diagne N, Sokhna C, Lemasson JJ, Diatta M, Konaté L, Faye F, Rogier C, Trape JF: **High annual and seasonal variations in malaria transmission by *anophelines* and vector species composition in Dielmo, a holoendemic area in Senegal.** *Am J Trop Med Hyg* 1997, **56**:247–253.
89. Trape JF, Rogier C, Konaté L, Diagne N, Bouganali H, Canque B, Legros F, Badji A, Ndiaye G, Ndiaye P, Brahimi K, Faye O, Druilhe P, Silva LPD: **The Dielmo project: a longitudinal study of natural malaria infection and the mechanisms of protective immunity in a community living in a holoendemic area of Senegal.** *Am J Trop Med Hyg* 1994, **51**:123–137.

90. Robert V, Dieng H, Lochouam L, Traoré SF, Trape JF, Simondon F, Fontenille D: **La transmission du paludisme dans la zone de Niakhar, Sénégal.** *Trop Med Int Health* 1998, **3**:667–677.

91. Faye O, Fontenille D, Herve JP, Diack PA, Mouchet SDJ: **Le paludisme en zone sahelienne du Sénégal. 1. Données entomologiques sur la transmission.** *Ann Soc Belg Med Trop* 1993, **73**:21–30.

92. Fontenille D, Lochouarn L, Diatta M, Sokhna C, Dia I, Diagne N, Lemasson JJ, Ba K, Tall A, Rogier C, Trape JF: **Four years' entomological study of the transmission of seasonal malaria in Senegal and the bionomics of *Anopheles gambiae* and *A. arabiensis*.** *Trans R Soc Trop Med Hyg* 1997, **91**:647–652.

93. Ndiaye O, Hesran JYL, Etard JF, Diallo A, Simondon F, Ward MN, Robert V: **Variations climatique et mortalité attribuée au paludisme dans la zone de Niakhar, Sénégal, de 1984 à 1996.** *Santé* 2001, **11**:25–33.

94. Vercruysse J, Jancloes M, van de Velden L: **Epidemiology of seasonal falciparum malaria in an urban area of Senegal.** *Bull World Health Org* 1983, **61**:821–831.

95. Vercruysse J, Jancloes M: **Etude entomologique sur la transmission du paludisme humain dans la zone urbaine de Pikine (Senegal).** *Cahiers O.R.S.T.O.M. Série Entomologie Médicale et Parasitologie* 1981, **19**:165–178.

96. Faye O, Gaye O, Fontenille D, Sy N, Konaté L, Hébrard G, Hervé JP, Trouillet J, Diallo S, Mouchet J: **Comparaison de la transmission du paludisme dans deux faciès épidémiologiques au Sénégal: la zone côtière sahêlienne et la zone méridionale soudanienne.** *Dakar Médical* 1995, **40**:201–207.

97. Boudin C, Diop A, Gaye A, Gadiaga L, Gouagna C, Safeukui I, Bonnet S: ***Plasmodium falciparum* transmission blocking immunity in three areas with perennial or seasonal endemicity and different levels of transmission.** *Am J Trop Med Hyg* 2005, **73**:1090–1095.

98. Faye O, Konaté L, Fontenille D, Gaye O, Sy N, Hébrard G, Hervé JP, Touré YT: **Variations saisonnières des populations d'*Anopheles gambiae s.l.* et transmission du paludisme dans un village de savane soudanienne du sud-est du Sénégal.** *Bulletin Institut Fondamental d'Afrique Noire: Série A* 1995, **48**:57–66.

99. Barnish G, Maude GH, Bockarie MJ, Erunkulu OA, Dumbuya MS, Greenwood BM: **Malaria in a rural area of Sierra Leone. II. Parasitological and related results from pre- and post-rains clinical surveys.** *Ann Trop Med Parasitol* 1993, **87**:137–148.

100. Bockarie MJ, Service MW, Barnish G, Maude GH, Greenwood BM: **Malaria in a rural area of Sierra Leone. III. Vector ecology and disease transmission.** *Ann Trop Med Parasitol* 1994, **88**:251–262.

101. Abdel-Wahab A, Abdel-Muhsin AMA, Ali E, Suleiman S, Ahmed S, Walliker D, Babiker HA: **Dynamics of gametocytes among *Plasmodium falciparum* clones in natural infections in an area of highly seasonal transmission.** *J Infect Dis* 2002, **185**:1838–1842.

102. Davis JC, Kemble TDCSK, Talemwa N, Njama-Meya D, Staedke SG, Dorsey G: **Longitudinal study of urban malaria in a cohort of Ugandan children: description of study site, census and recruitment.** *Malar J* 2006, **5**:18.
