# Supplementary material for: Development of a new version of the Liverpool Malaria Model. II. Calibration and validation for West Africa
Source: Malar J. 2011 Mar 16;10:62. doi: 10.1186/1475-2875-10-62 (PMC3070689; doi:10.1186/1475-2875-10-62)
Supplement: Additional file 3 — Skill scores in terms of the LMM validation. Ranks in terms of skill scores as computed for simulations of different LMM sets of parameter settings: (1) Top 10 and last 5 of 300 malaria runs from the first calibration step according to the skill score of the annual human biting and entomological inoculation rates (SC(HBRa;EIRa)). (2) Top 10 of 300 malaria runs from the first calibration step in terms of all eleven entomological and parasitological malaria variables (SC(all)). (3) Top 10 of 455 malaria runs from the second calibration step relative to SC(HBRa,EIRa). (4) Top 10 of 455 malaria runs from the second calibration step regarding SC(all). [file 1475-2875-10-62-S3.PDF]

# 3 Skill scores in terms of the LMM validation

Ranks in terms of skill scores as computed for simulations of different LMM sets of parameter settings.

## 3.1 Top 10 and last 5 of 375 LMM versions from the first calibration step according to $SC(HBR_a, EIR_a)$

Top 10 and last 5 of 375 LMM versions from the first calibration step according to the skill score relative to the annual human biting and entomological inoculation rates ($SC(HBR_a, EIR_a)$). The numbers in brackets refer to points that could be theoretically achieved. Note that the dry season mosquito survival probability shift ($p_{d\downarrow}$) was set to zero in the initial runs.

Columns: rank: ranking with regard to $SC(HBR_a, EIR_a)$; $CAP$: setting of the cap on the number of fertile females ($CAP$); $\#E_p$: setting of the number of produced eggs per female mosquito ($\#E_p$); $S$: setting of the most suitable rainfall condition ($S$) regarding the fuzzy distribution model (in mm); $U_2$: setting of the upper threshold of unsuitable rainfall conditions ($U_2$) in terms of the fuzzy distribution model (in mm). $SC(x)$ denotes the skill score with regard to variable $x$.

| **rank** | $CAP$ | $\#E_p$ | $S$ | $U_2$ | $SC(HBR_a)$ | $SC(EIR_a)$ | $SC(HBR_a, EIR_a)$ | $SC(\text{all})$ |
|---|---|---|---|---|---|---|---|---|
| 1 | 750 | 50 | 5 | 500 | 34 (52) | 39 (54) | 73 (106) | 251 (440) |
| 2 | 750 | 50 | 10 | 500 | 31 (52) | 41 (54) | 72 (106) | 256 (440) |
| 3 | 500 | 75 | 15 | 500 | 31 (52) | 38 (54) | 69 (106) | 256 (440) |
| 4 | 500 | 100 | 15 | 500 | 36 (52) | 32 (54) | 68 (106) | 246 (440) |
| 5 | 500 | 75 | 10 | 500 | 34 (52) | 33 (54) | 67 (106) | 245 (440) |
| 6 | 250 | 150 | 15 | 500 | 32 (52) | 34 (54) | 66 (106) | 240 (440) |
|  | 250 | 125 | 15 | 500 | 29 (52) | 37 (54) | 66 (106) | 243 (440) |
| 8 | 750 | 75 | 20 | 500 | 31 (52) | 34 (54) | 65 (106) | 255 (440) |
|  | 1000 | 50 | 10 | 500 | 35 (52) | 30 (54) | 65 (106) | 251 (440) |
| 10 | 500 | 100 | 20 | 500 | 30 (52) | 34 (54) | 64 (106) | 255 (440) |
| 371 | 2000 | 150 | 5 | 500 | -15 (52) | -57 (54) | -72 (106) | 74 (440) |
| 372 | 2000 | 125 | 5 | 1000 | -17 (52) | -56 (54) | -73 (106) | 64 (440) |
| 373 | 2000 | 150 | 10 | 1000 | -21 (52) | -53 (54) | -74 (106) | 78 (440) |
| 374 | 2000 | 150 | 5 | 750 | -22 (52) | -61 (54) | -83 (106) | 54 (440) |
| 375 | 2000 | 150 | 5 | 1000 | -23 (52) | -62 (54) | -85 (106) | 50 (440) |

### 3.2 Top 10 of 375 LMM versions from the first calibration step in terms of $SC(all)$

Top 10 of 375 LMM versions from the first calibration step according to the skill score of all eleven entomological and parasitological malaria variables.

Columns and brackets: as above.

| **rank** | *CAP* | $\#E_p$ | $S$ | $U_2$ | $SC(HBR_a)$ | $SC(CSPR_a)$ | $SC(EIR_a)$ | $SC(Seas)$ | $SC(XSeas)$ | $SC(MSeas)$ | $SC(SSeas)$ | $SC(ESeas)$ | $SC(PR_a)$ | $SC(PR_{a,max})$ | $SC(PR_{a,min})$ | $SC(\text{all})$ |
|---|---|---|---|---|---|---|---|---|---|---|---|---|---|---|---|---|
| 1 | 750 | 75 | 30 | 500 | 27 (52) | 27 (55) | 34 (54) | 33 (42) | 19 (41) | 33 (37) | 26 (41) | 24 (37) | 13 (29) | 16 (25) | 7 (27) | 259 (440) |
| 2 | 1000 | 50 | 15 | 500 | 29 (52) | 32 (55) | 32 (54) | 32 (42) | 18 (41) | 29 (37) | 28 (41) | 21 (37) | 14 (29) | 15 (25) | 8 (27) | 258 (440) |
| 3 | 250 | 150 | 30 | 500 | 23 (52) | 32 (55) | 35 (54) | 30 (42) | 21 (41) | 30 (37) | 29 (41) | 21 (37) | 13 (29) | 15 (25) | 8 (27) | 257 (440) |
| 4 | 750 | 50 | 10 | 500 | 31 (52) | 28 (55) | 41 (54) | 31 (42) | 19 (41) | 29 (37) | 26 (41) | 17 (37) | 13 (29) | 13 (25) | 8 (27) | 256 (440) |
| | 500 | 75 | 15 | 500 | 31 (52) | 31 (55) | 38 (54) | 25 (42) | 20 (41) | 30 (37) | 27 (41) | 17 (37) | 15 (29) | 14 (25) | 8 (27) | 256 (440) |
| 6 | 750 | 75 | 20 | 500 | 31 (52) | 30 (55) | 34 (54) | 29 (42) | 20 (41) | 30 (37) | 28 (41) | 19 (37) | 13 (29) | 13 (25) | 8 (27) | 255 (440) |
| | 750 | 50 | 15 | 500 | 24 (52) | 30 (55) | 36 (54) | 32 (42) | 18 (41) | 29 (37) | 28 (41) | 21 (37) | 14 (29) | 15 (25) | 8 (27) | 255 (440) |
| | 500 | 150 | 30 | 500 | 32 (52) | 33 (55) | 29 (54) | 29 (42) | 21 (41) | 27 (37) | 29 (41) | 21 (37) | 12 (29) | 14 (25) | 8 (27) | 255 (440) |
| | 500 | 100 | 20 | 500 | 30 (52) | 31 (55) | 34 (54) | 29 (42) | 20 (41) | 30 (37) | 29 (41) | 18 (37) | 13 (29) | 13 (25) | 8 (27) | 255 (440) |
| 10 | 1000 | 75 | 30 | 500 | 29 (52) | 23 (55) | 29 (54) | 33 (42) | 19 (41) | 33 (37) | 26 (41) | 24 (37) | 14 (29) | 17 (25) | 7 (27) | 254 (440) |

### 3.3 Top 10 of 455 LMM versions from the second calibration step relative to $SC(HBR_a, EIR_a)$

Top 10 of 455 LMM versions from the second calibration step in terms of $SC(HBR_a, EIR_a)$. Note that in the second set of runs $S$ and $U_2$ are set to 10 and 500 mm, respectively.

Columns and brackets: as above; $p_{d\downarrow}$: setting of $p_{d\downarrow}$ (in %).

| **rank** | *CAP* | $p_{d\downarrow}$ | $\#E_p$ | $SC(HBR_a)$ | $SC(EIR_a)$ | $SC(HBR_a, EIR_a)$ | $SC(\text{all})$ |
|---|---|---|---|---|---|---|---|
| 1 | 400 | -10 | 120 | 37 (52) | 41 (54) | 78 (106) | 279 (440) |
| 2 | 700 | -7.5 | 70 | 34 (52) | 41 (54) | 75 (106) | 270 (440) |
| | 650 | -10 | 80 | 34 (52) | 41 (54) | 75 (106) | 268 (440) |
| | 550 | -10 | 90 | 35 (52) | 40 (54) | 75 (106) | 274 (440) |
| | 500 | -10 | 90 | 35 (52) | 40 (54) | 75 (106) | 272 (440) |
| | 500 | -10 | 100 | 35 (52) | 40 (54) | 75 (106) | 272 (440) |
| | 500 | -7.5 | 90 | 35 (52) | 40 (54) | 75 (106) | 277 (440) |
| | 350 | -10 | 130 | 35 (52) | 40 (54) | 75 (106) | 281 (440) |
| 9 | 750 | -10 | 70 | 34 (52) | 40 (54) | 74 (106) | 266 (440) |
| | 700 | -10 | 70 | 34 (52) | 40 (54) | 74 (106) | 266 (440) |

### 3.4 Top 10 of 455 LMM versions from the second calibration step with regard to $SC(all)$

Top 10 of 455 LMM versions from the second calibration step regarding $SC(all)$. Note that in the second set of runs $S$ and $U_2$ are set to 10 and 500 mm, respectively.

Columns and brackets: as the former table.

| **rank** | *CAP* | $p_{d\downarrow}$ | $\#E_p$ | $SC(HBR_a)$ | $SC(CSPR_a)$ | $SC(EIR_a)$ | $SC(Seas)$ | $SC(XSeas)$ | $SC(MSeas)$ | $SC(SSeas)$ | $SC(ESeas)$ | $SC(PR_a)$ | $SC(PR_{a,max})$ | $SC(PR_{a,min})$ | $SC(\text{all})$ |
|---|---|---|---|---|---|---|---|---|---|---|---|---|---|---|---|
| 1 | 350 | -7.5 | 120 | 34 (52) | 32 (55) | 40 (54) | 32 (42) | 22 (41) | 30 (37) | 30 (41) | 22 (37) | 16 (29) | 16 (25) | 8 (27) | 282 (440) |
| | 300 | -7.5 | 130 | 33 (52) | 33 (55) | 40 (54) | 32 (42) | 23 (41) | 30 (37) | 30 (41) | 22 (37) | 16 (29) | 15 (25) | 8 (27) | 282 (440) |
| 3 | 350 | -10 | 130 | 35 (52) | 33 (55) | 40 (54) | 31 (42) | 23 (41) | 29 (37) | 30 (41) | 20 (37) | 16 (29) | 16 (25) | 8 (27) | 281 (440) |
| 4 | 450 | -7.5 | 100 | 34 (52) | 32 (55) | 40 (54) | 32 (42) | 21 (41) | 28 (37) | 31 (41) | 22 (37) | 16 (29) | 15 (25) | 8 (27) | 279 (440) |
| | 400 | -10 | 120 | 37 (52) | 33 (55) | 41 (54) | 31 (42) | 23 (41) | 28 (37) | 28 (41) | 18 (37) | 16 (29) | 16 (25) | 8 (27) | 279 (440) |
| | 400 | -7.5 | 110 | 34 (52) | 30 (55) | 40 (54) | 32 (42) | 22 (41) | 29 (37) | 30 (41) | 22 (37) | 16 (29) | 16 (25) | 8 (27) | 279 (440) |
| 7 | 800 | -7.5 | 80 | 35 (52) | 32 (55) | 36 (54) | 34 (42) | 20 (41) | 27 (37) | 31 (41) | 22 (37) | 17 (29) | 16 (25) | 8 (27) | 278 (440) |
| | 400 | -7.5 | 120 | 34 (52) | 32 (55) | 35 (54) | 33 (42) | 22 (41) | 29 (37) | 30 (41) | 23 (37) | 16 (29) | 16 (25) | 8 (27) | 278 (440) |
| | 350 | -7.5 | 130 | 34 (52) | 33 (55) | 35 (54) | 33 (42) | 22 (41) | 30 (37) | 30 (41) | 23 (37) | 16 (29) | 14 (25) | 8 (27) | 278 (440) |
| | 350 | -5 | 110 | 32 (52) | 34 (55) | 40 (54) | 28 (42) | 23 (41) | 31 (37) | 31 (41) | 21 (37) | 16 (29) | 14 (25) | 8 (27) | 278 (440) |
