# Supplementary material for: Development of a new version of the Liverpool Malaria Model. II. Calibration and validation for West Africa
Source: Malar J. 2011 Mar 16;10:62. doi: 10.1186/1475-2875-10-62 (PMC3070689; doi:10.1186/1475-2875-10-62)
Supplement: Additional file 4 — Observed and simulated entomological and parasitological values of the LMM2010. Validation of LMM2010 simulations in terms of HBRa, CSPRa, Seas, MSeas, XSeas, SSeas, PRa, and PRmax, a in the area of the 34 synoptic stations in West Africa as ordered in Figure 2. [file 1475-2875-10-62-S4.PDF]

4 Observed and simulated entomological and parasitological values of the LMM_{2010}

Validation of LMM_{2010} simulations in terms of HBR_a, $CSPR_a$, *Seas*, *MSeas*, PR_a, and $PR_{max,a}$ as well as *XSeas* and *SSeas* in the area of the 34 synoptic stations in West Africa as ordered in Figure 2.

Regarding HBR_a, $CSPR_a$, *Seas*, *MSeas*, PR_a, and $PR_{max,a}$ the simulated 34 annual values between 1973 and 2006 are illustrated as grey box-and-whisker plots (the numeric values of maxima beyond the scale of the ordinate are plotted on the upper abscissa). Field observations (green lines and box plots) are either displayed as a vertical line (two available measurements), a vertical line with the median (three or four values), or as a box-and-whisker plot ($\geq$ five data points). Each observation is furthermore inserted as a red circle and the number of observations is given above the entered observations (red digits).

With regard to *XSeas*, *SSeas*, and *ESeas* each month is given a colour-coded rectangle representing the occasions for years between 1973 and 2006, when the malaria season finished in the model simulations. The simulated data (colour-filled rectangles) are compared to observed values (inserted as a digit). The frequency distribution (in numbers) regarding the simulated 34 values for 1973-2006 is given for each month. The frequencies of years with no ('no') and year-around ('C') transmission are also illustrated in the lowermost and topmost rows, respectively.

The skill score in terms of the particular variable ($SC(x)$), a measure of the performance of the simulations with regard to observed data, is denoted for every station as a blue digit.

Annual human biting rate (HBR_a)

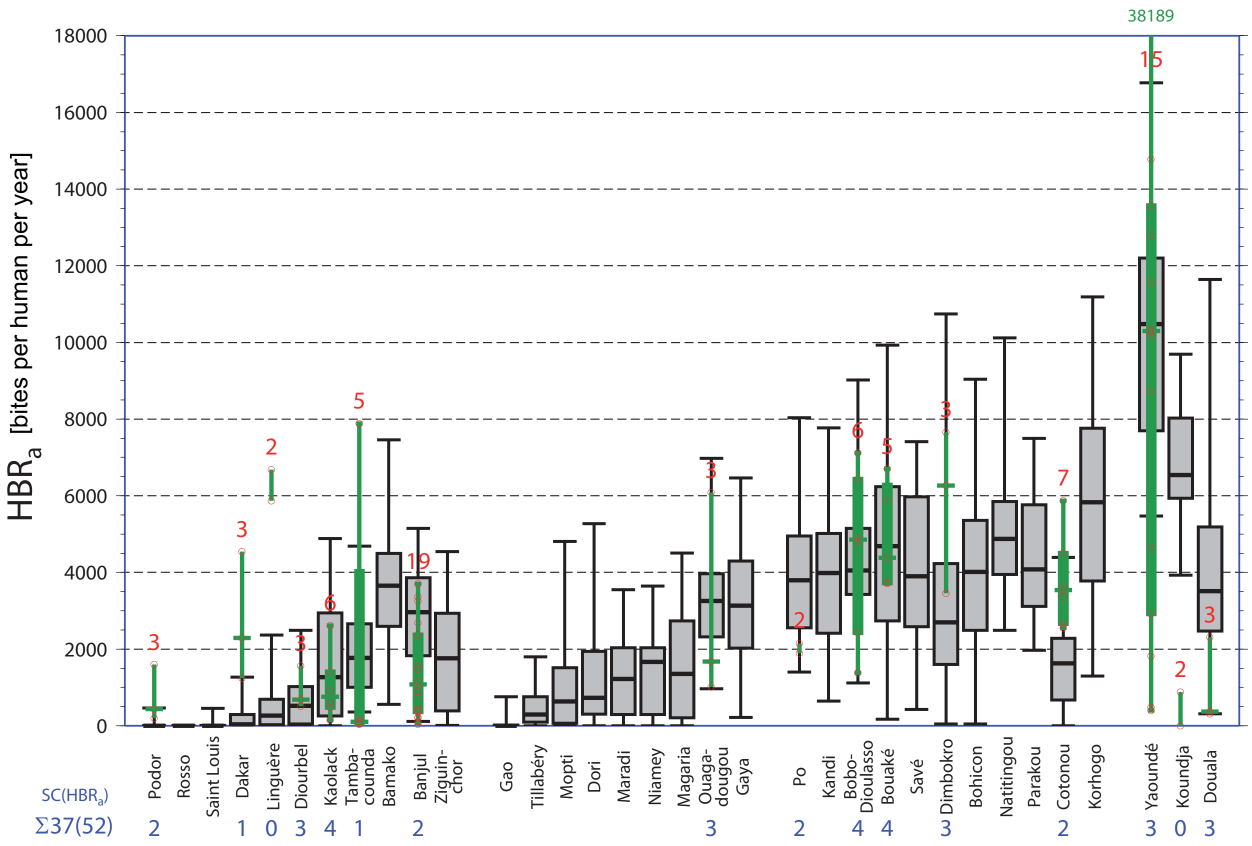

Supplementary Figure 1: Same as Figure 3 but for HBR_a.

Annual mean circumsporozoite rate ($CSPR_a$)

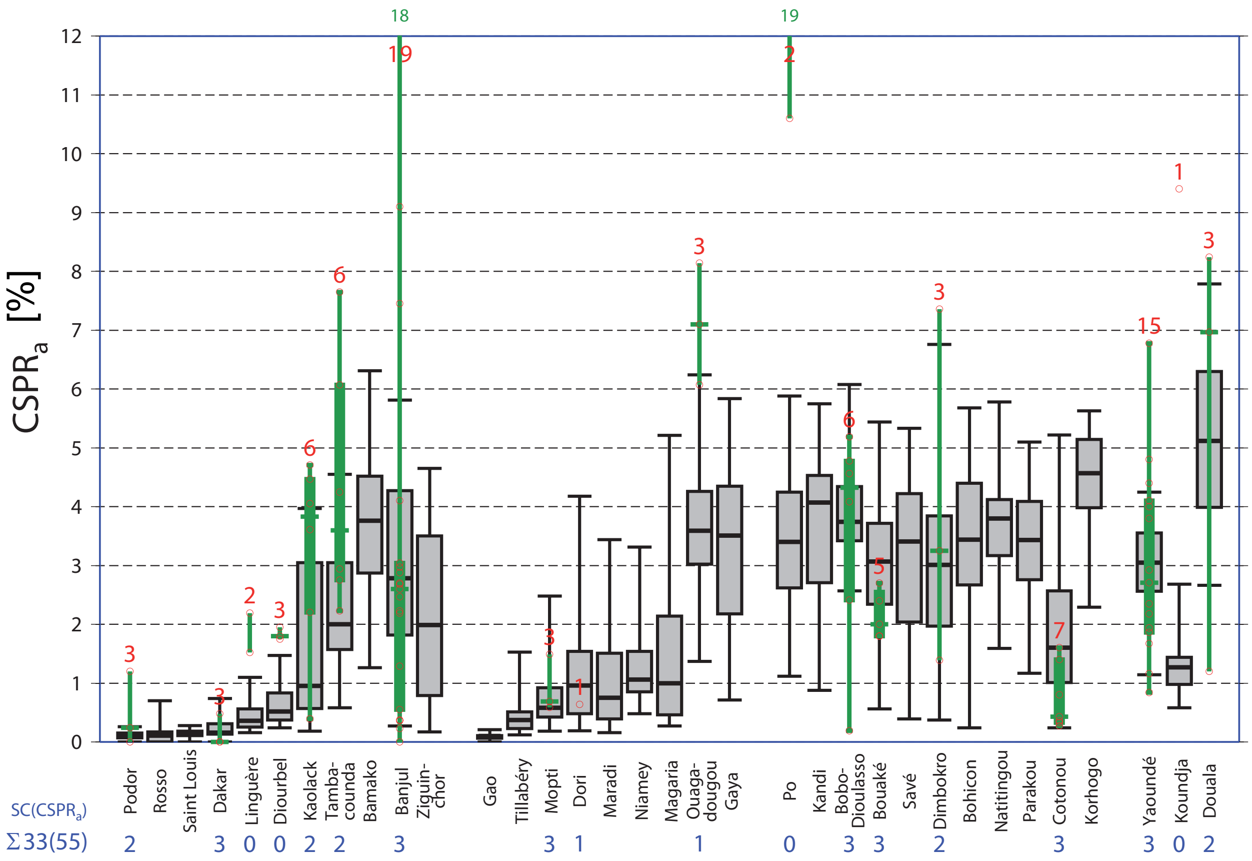

Supplementary Figure 2: Same as Figure 3 but for $CSPR_a$.

Length of the malaria season (*Seas*)

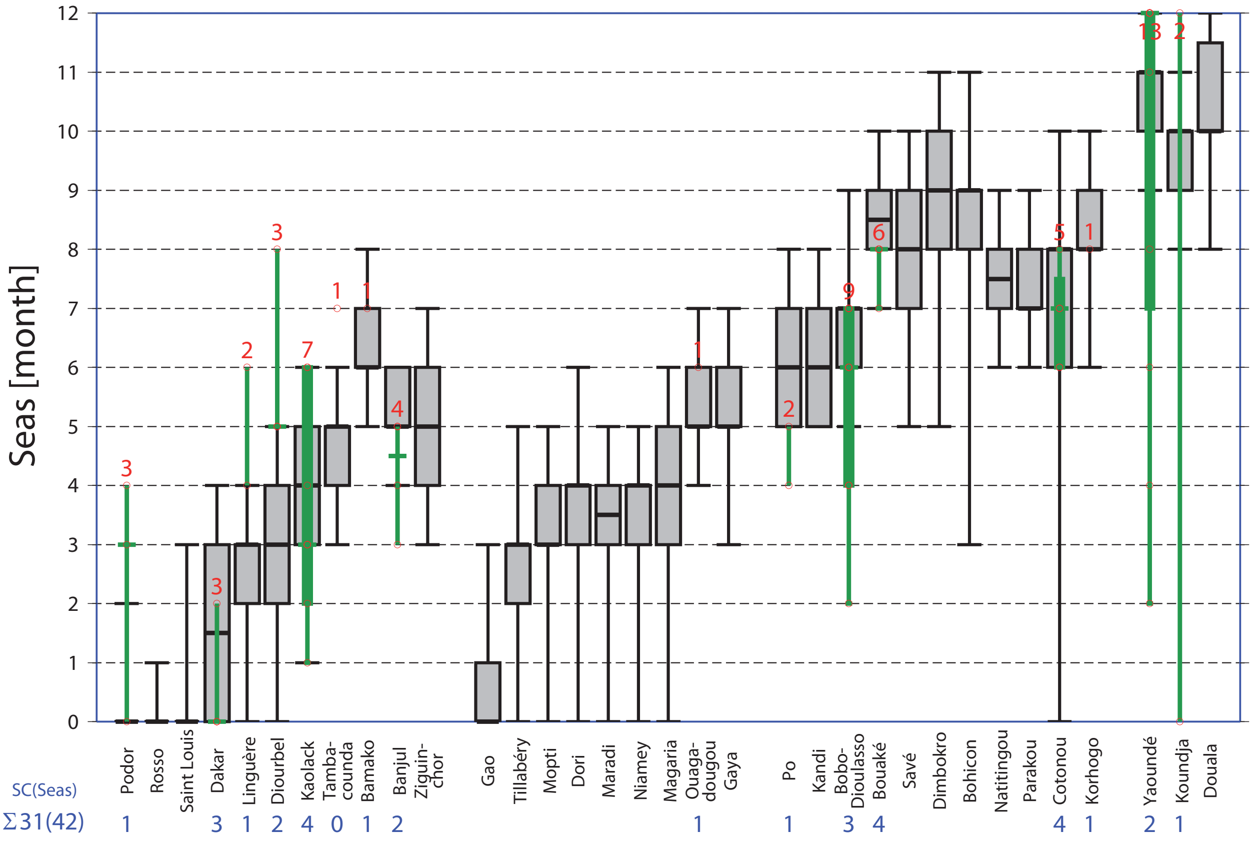

Supplementary Figure 3: Same as Figure 3 but for *Seas*.

Length of the main malaria season (*MSeas*)

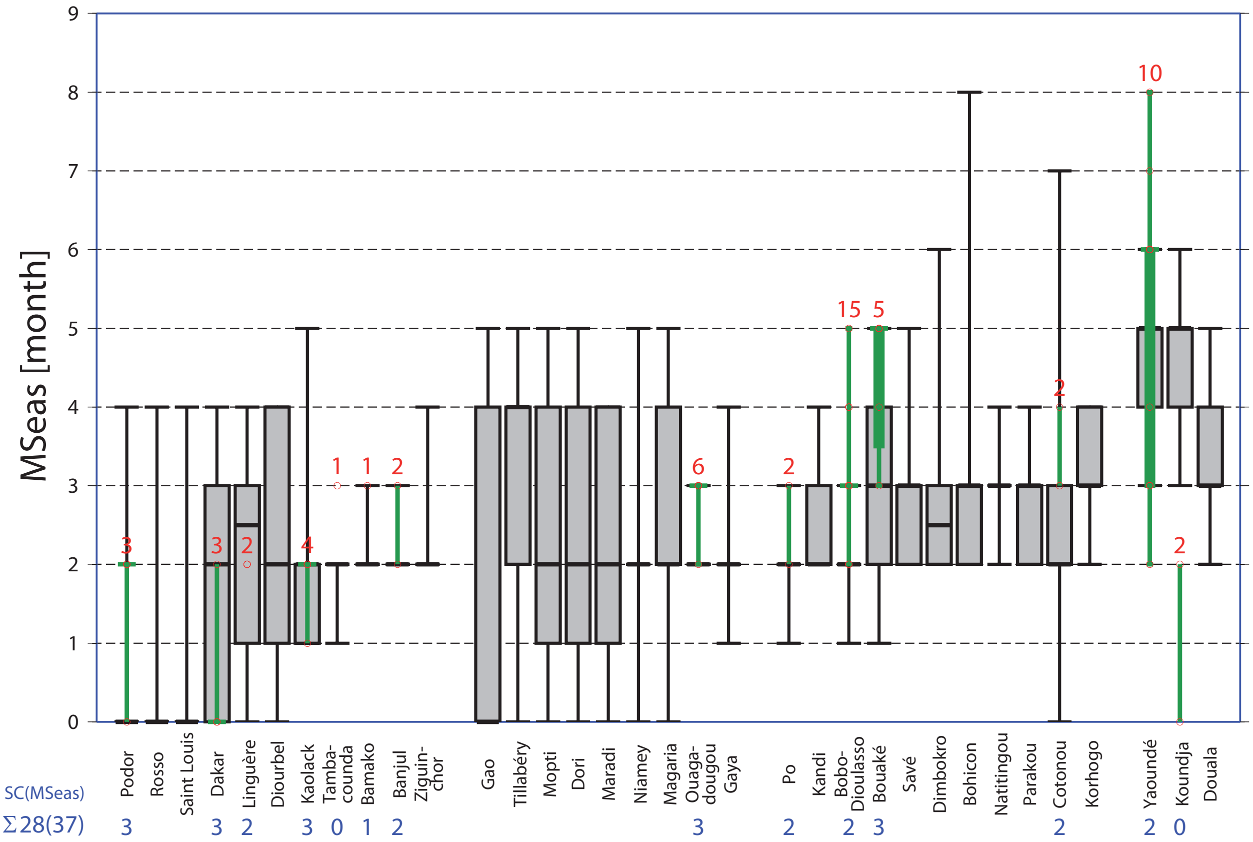

Supplementary Figure 4: Same as Figure 3 but for *MSeas*.

Annual mean asexual parasite ratio (PR_a)

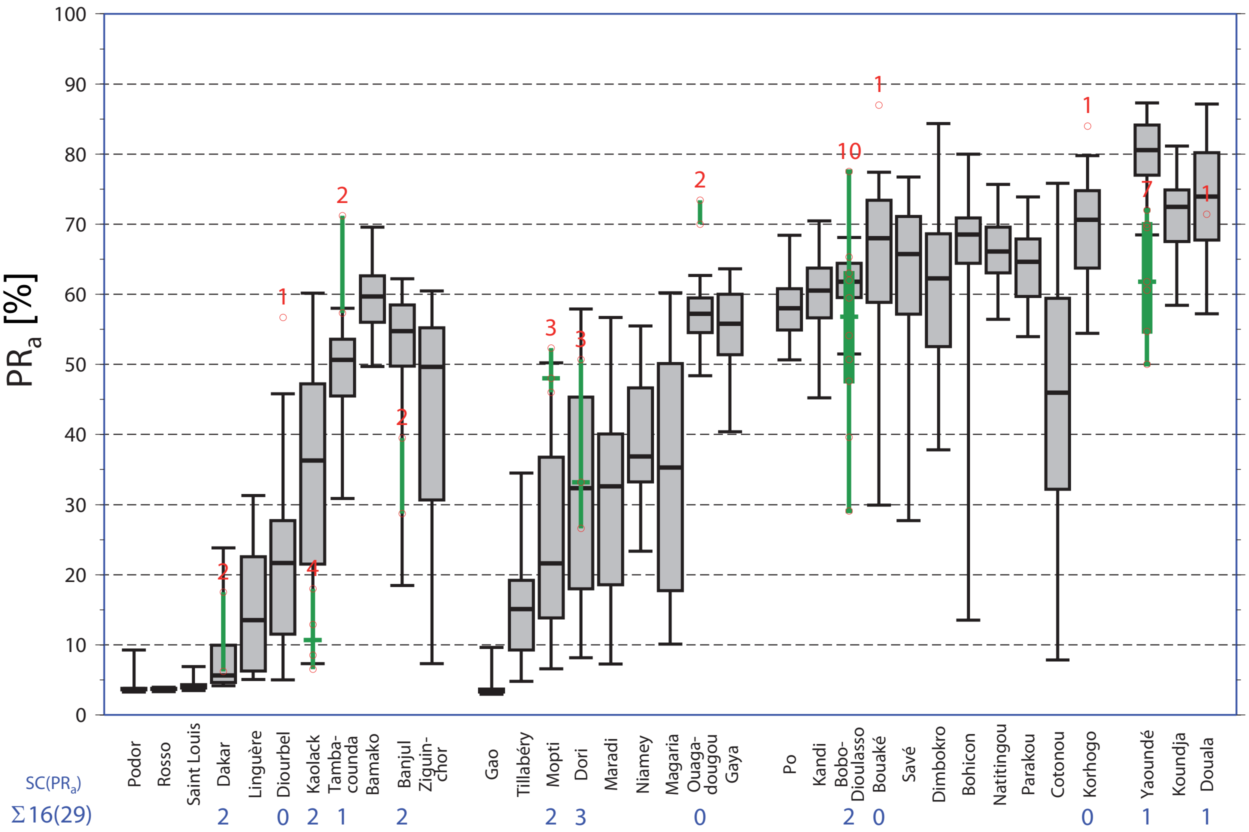

Supplementary Figure 5: Same as Figure 3 but for PR_a.

Annual maximum of the asexual parasite ratio ($PR_{max,a}$)

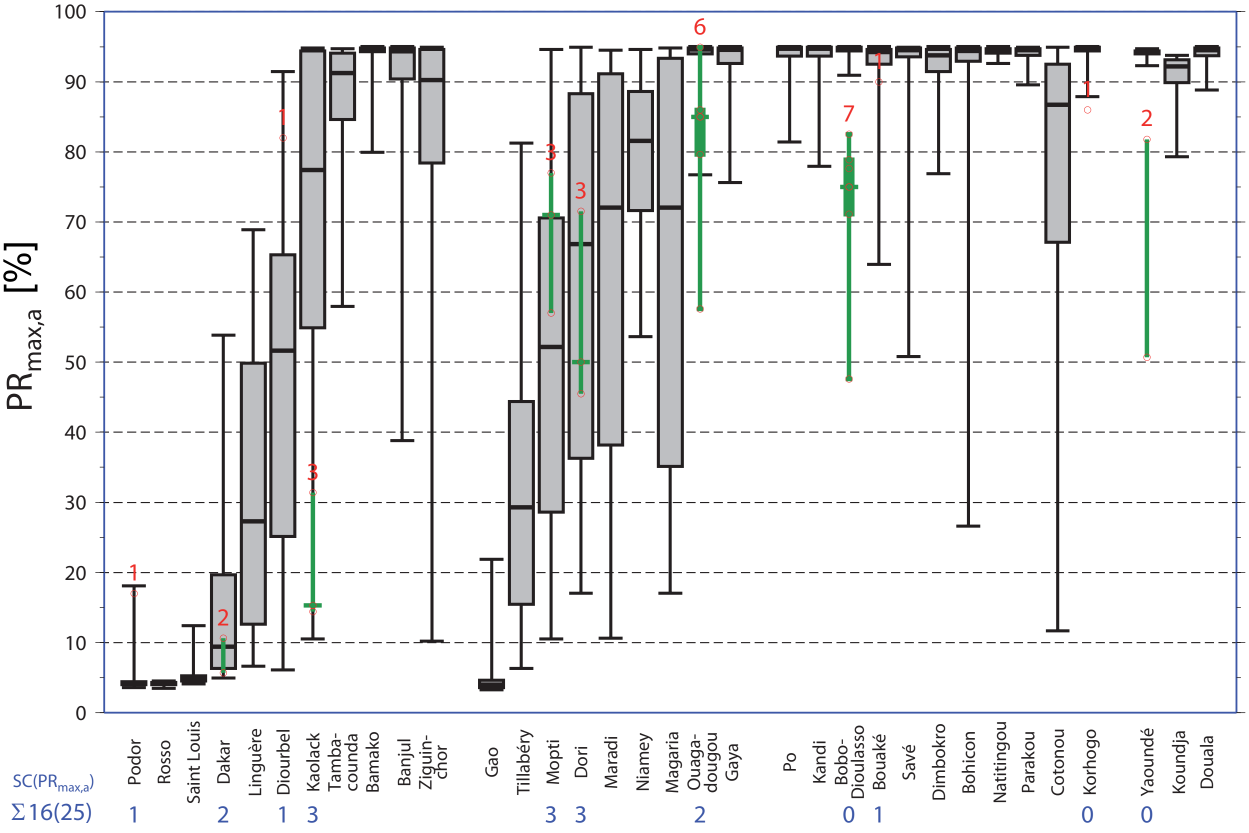

Supplementary Figure 6: Same as Figure 3 but for $PR_{max,a}$.

Month of maximum malaria transmission (*XSeas*)

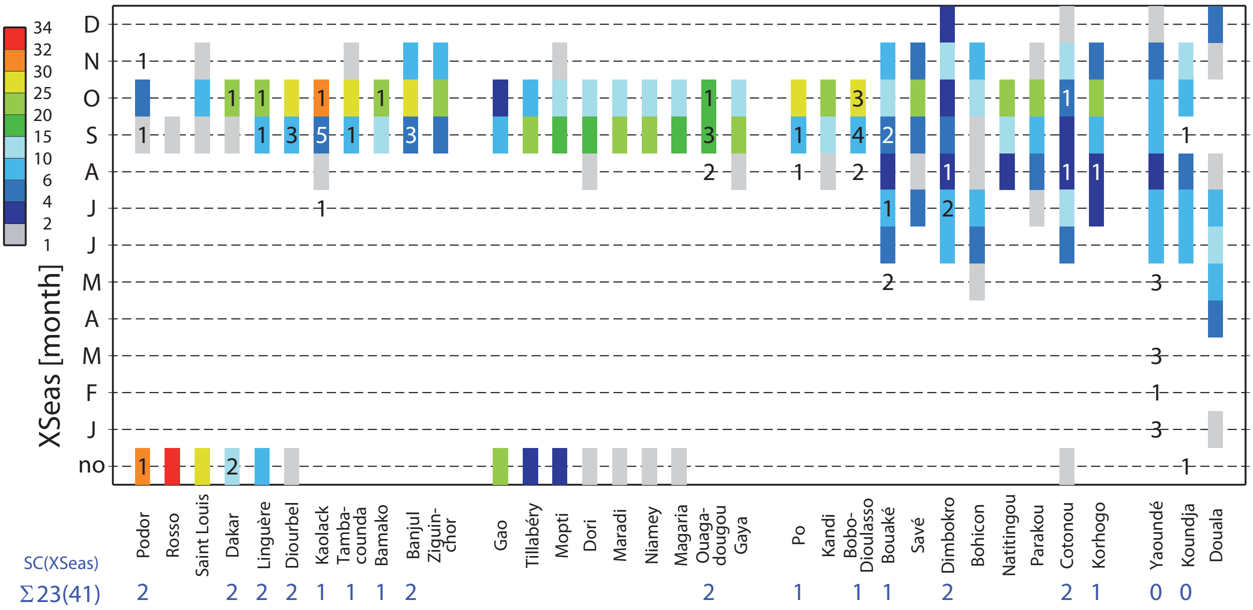

Supplementary Figure 7: Same as Figure 4 but for *XSeas*.

Start of the malaria season (*SSeas*)

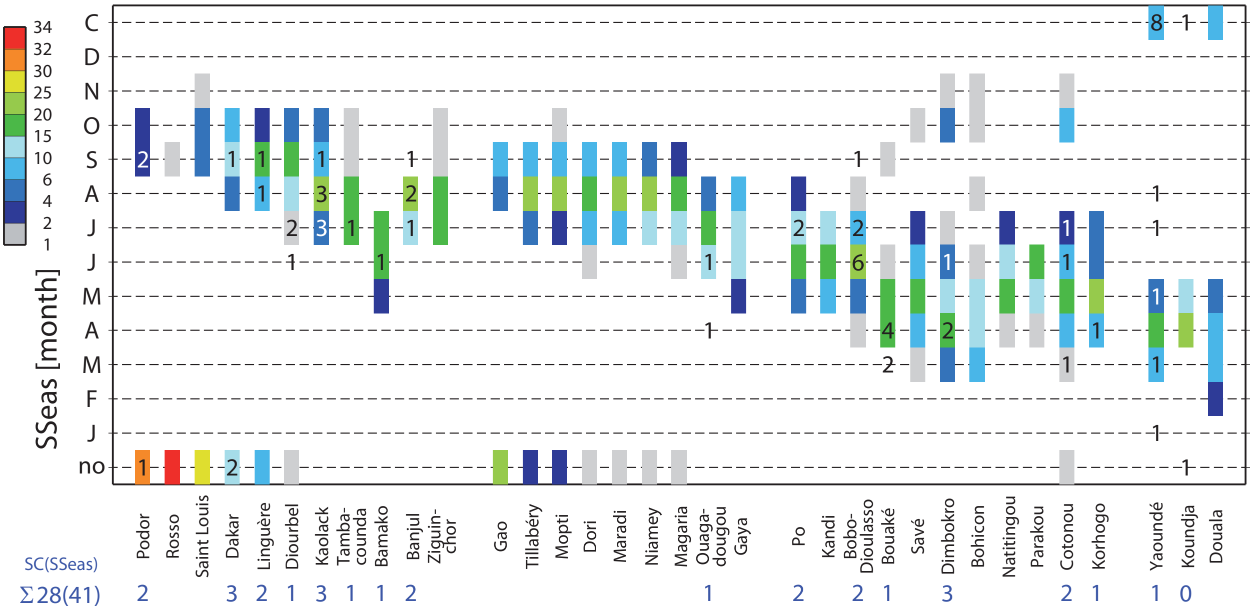

Supplementary Figure 8: Same as Figure 4 but for *SSeas*.
